# Identification of a Novel Calcium Binding Motif Based on the Detection of Sequence Insertions in the Animal Peroxidase Domain of Bacterial Proteins

**DOI:** 10.1371/journal.pone.0040698

**Published:** 2012-07-13

**Authors:** Saray Santamaría-Hernando, Tino Krell, María-Isabel Ramos-González

**Affiliations:** Department of Environmental Protection, Estación Experimental de Zaidín-Consejo Superior de Investigaciones Científicas (CSIC), Granada, Spain; University of Oldenburg, Germany

## Abstract

Proteins of the animal heme peroxidase (ANP) superfamily differ greatly in size since they have either one or two catalytic domains that match profile PS50292. The orf PP_2561 of *Pseudomonas putida* KT2440 that we have called PepA encodes a two-domain ANP. The alignment of these domains with those of PepA homologues revealed a variable number of insertions with the consensus G-x-D-G-x-x-[GN]-[TN]-x-D-D. This motif has also been detected in the structure of pseudopilin (pdb 3G20), where it was found to be involved in Ca^2+^ coordination although a sequence analysis did not reveal the presence of any known calcium binding motifs in this protein. Isothermal titration calorimetry revealed that a peptide containing this consensus motif bound specifically calcium ions with affinities ranging between 33–79 µM depending on the pH. Microcalorimetric titrations of the purified N-terminal ANP-like domain of PepA revealed Ca^2+^ binding with a *K_D_* of 12 µM and stoichiometry of 1.25 calcium ions per protein monomer. This domain exhibited peroxidase activity after its reconstitution with heme. These data led to the definition of a novel calcium binding motif that we have termed PERCAL and which was abundantly present in animal peroxidase-like domains of bacterial proteins. Bacterial heme peroxidases thus possess two different types of calcium binding motifs, namely PERCAL and the related hemolysin type calcium binding motif, with the latter being located outside the catalytic domains and in their C-terminal end. A phylogenetic tree of ANP-like catalytic domains of bacterial proteins with PERCAL motifs, including single domain peroxidases, was divided into two major clusters, representing domains with and without PERCAL motif containing insertions. We have verified that the recently reported classification of bacterial heme peroxidases in two families (cd09819 and cd09821) is unrelated to these insertions. Sequences matching PERCAL were detected in all kingdoms of life.

## Introduction

Bacterial as well as eukaryotic proteins have evolved to recognize calcium ions by a number of structural motifs which can be identified by specific consensus sequences. In eukaryotic cells Ca^2+^ is a common intracellular second-messenger molecule and impacts nearly every aspect of cellular life [1]. In bacteria, it is known that calcium has an important structural role in guaranteeing the integrity of the outer lipopolysaccharide layer and the cell wall [2]. The increasing number of proteins containing Ca^2+^-binding motifs supports the importance of calcium in protein stability, enzymatic activity or signal transduction [3]. Several types of Ca^2+^ binding motifs have been identified in bacterial proteins. These include hemolysin-type calcium-binding (HTCaB) region [4], EF-hand [3] and EF-hand like domains [5], [6], [7].

HTCaB containing proteins are extracellular, exported by type I secretion systems [8] and are often determinants of pathogenesis like the hemolysins of several gram negative pathogenic bacteria or symbiosis. Examples include the alkaline protease of *Pseudomonas aeruginosa* and the nodulation protein NodO. The alkaline protease adopts a beta-roll structure and calcium ions are bound in the loop regions connecting the strands of the roll. Calcium coordination is primarily achieved by interaction with aspartate side chains and glycin oxo-groups [4]. NodO from nitrogen fixing bacteria presents in its C-terminal end an HTCaB-related calcium binding signature, which consists of a multiple tandem repeat of a nonapeptide [9].

Multicellular behavior of the beneficial bacterium *Pseudomonas putida* KT2440 is sustained by two large extracellular bacterial adhesins, termed LapA and LapF [10]. LapA presents four tandem repeats of HTCaB and LapF three tandem repeats of the NodO calcium binding signature in their C-terminal ends [11]. In the same bacterium there is a third large extracellular protein, encoded by PP_2561, which also has a C-terminal fragment rich in HTCaB repeats. This protein is an important bacterial determinant of plant root colonization and induced systemic resistance against phytopathogens [12]. Remarkably this protein also presents HTCaB sites located on C-terminal extensions of two animal peroxidase-like (ANP-like) domains.

In spite of its designation, proteins of the animal peroxidase_like superfamily are not limited to metazoans and are also found in fungi or plants. This superfamily includes animal heme peroxidases (ANHEMP) and related proteins. The mammalian goat lactoperoxidase [13] and human myeloperoxidase [14] are among the best characterized members of this superfamily. More recently two so far uncharacterized novel families (cd09819 and cd09821) of bacterial heme peroxidases (BACHEMP) have been defined within this superfamily [15]. Members of these families have been found in Proteobacteria, Cyanobacteria, Actinobacteria and Chlamydiae; however their distribution is not ubiquitous within these taxons and often only a small number of strains encode homologues in their genomes. Enzymes of this class had previously been shown to oxidize Mn (II) in fungi [16] and α-proteobacteria [17]. However, an in-frame deletion of a PP_2561 homologue in *Pseudomonas putida* GB-1 did not compromise Mn (II) oxidation in this strain [18] as an indication that this activity may not be mediated by BACHEMP in *P. putida*. Even though the biological relevance of the HTCaB sites present in PP_2561 is unknown, it has been suggested that it may be related to the Ca^2+^-mediated modulation of the Mn (II) oxidation activity as observed for a related ANHEMP in *Aurantimonas* [17].

In this work we have examined in detail the sequence of the ANP-like domains of the protein encoded by PP_2561, renamed here as PepA (*Pseudomonas* extracellular peroxidase) and those of its homologues in other species. We were able to identify multiple short inserts of conserved sequence within these catalytic domains. Microcalorimetric titration of a peptide containing this inserts consensus sequence and of the purified N-terminal ANP-like domain of PepA revealed that these inserts bind specifically Ca^2+^ ions. Since this consensus did not match any of the previously defined sequences of calcium binding motifs (Table 1), we were able to define a novel motif, which was termed PERCAL (peroxidase calcium binding site). Sequence database searches strongly suggested that the PERCAL motif is abundantly present in pro- and eukaryotes.

**Table 1:** Summary of Ca-binding patterns.

Pattern name^a^	Motif signature	Source/Reference
**Cadherin domain**		PROSITE
PS00232 (signature)	[LIV]-x-[LIV]-x-D-x-N-D-[NH]-x-P	
PS50268 (profile)	106 residues	
**Calcium-binding EGF-like domain**		PROSITE
PS01187 (signature)	[DEQN]-x-[DEQN](2)-C-x(3,14)-C-x(3,7)-C-x-[DN]-x(4)-[FY]-x-C	
**Cysteine proteinase, calpain-type, catalytic domain**		PROSITE
PS50203 (profile)	301 residues	
**EF-hand calcium binding domain**		PROSITE
PS00018 (signature)	D-{W}-[DNS]-{ILVFYW}-[DENSTG]-[DNQGHRK]-{GP}-[LIVMC]-[DENQSTAGC]-x(2)-[DE]-[LIVMFYW]	
**Excalibur** b	D-x-D-x-D-G-x(2)-C-E	[4]
**Fibrinogen C-terminal domain**		PROSITE
PS00514 (signature)	W-W-[LIVMFYW]-x(2)-C-x(2)-[GSA]-x(2)-N-G	
PS51406 (profile)	220 residues	
**Hemolysin-type calcium binding region (HTCaB)**		PROSITE
PS00330 (signature)	D-x-[LI]-x(4)-G-x-D-x-[LI]-x-G-G-x(3)-D	
**NodO calcium binding motif**		[9]
PR00313 (signature)	Tandem repeat of a nonapeptide	
**Osteonectin domain**		PROSITE
PS00612 (signature 1)	C-x-[DNS]-x(2)-C-x(2)-G-[KRH]-x-C-x(6,7)-P-x-C-x-C-x(3,5)-C-P	
PS00613 (signature 2)	F-P-x-R-[IM]-x-D-W-L-x-[NQ]	
**S-100/ICaBP type calcium binding protein**		PROSITE
PS00303 (signature)	[LIVMFYW](2)-x(2)-[LKQ]-D-x(3)-[DN]-x(3)-[DNSG]-[FY]-x-[ES]-[FYVC]-x(2)-[LIVMFS]-[LIVMF]	
**Peroxidase Calcium binding site (PERCAL)**	**G-x-D-G-x(2)- [GN]-[TN]-x-D-D**	**This work**

a Signatures and profile PROSITE designation (at www.PROSITE.expasy.org/) are included.

b An extracellular variant of EF-hand in bacteria.

## Results

### Identification of Sequence Inserts that are Rich in Glycine and Aspartic Acid within Animal Peroxidase-like Domains of Bacterial Proteins

**Figure 1:**
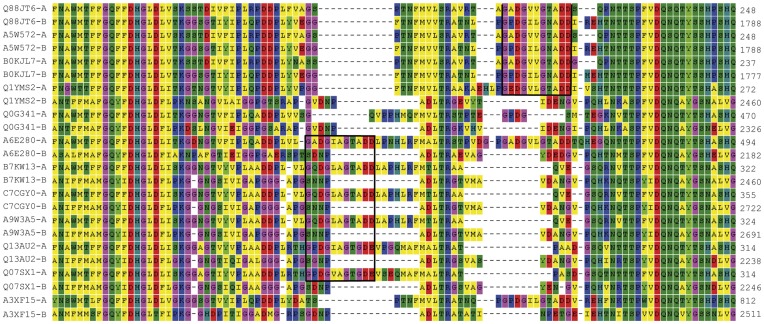
Section of the sequence alignment of ANP-like domains from 12 two-domain bacterial proteins. ANP-like domains as defined by PS50292 were aligned with MEGA 4.0. The numbers indicate the protein residues. The complete alignment is shown in figure S1. A region with two insertions (in boxes) matching the G-x-D-G-x(5)-D-D/E consensus and interrupting the major alignment are shown. The individual ANP-like domains are of *Fulvimarina pelagi* HTCC2506 (Q0G341), Manganese-oxidizing bacterium (strain SI85-9A1) (Q1YMS2), *Methylobacterium chloromethanicum* CM4 / NCIMB 13688 (B7KW13), *M. extorquens* DSM 5838 / DM4 (A9W3A5), *M. extorquens* PA1 (C7CGY0), *Pseudomonas putida* F1 (A5W572), *P. putida* GB1 (B0KJL7), *P. putida* KT2440 (Q88JT6), *Rhodopseudomonas palustris* BisA53 (Q07SX1), *Rhodopseudomonas palustris* BisB5 (Q13AU2), *Roseobacter* sp. MED193 (A3XF15) and *Roseovarius* sp. TM1035 (A6E280). Letters -A and -B refer to N- and C-terminal ANP-like domains, respectively.

**Figure 2:**
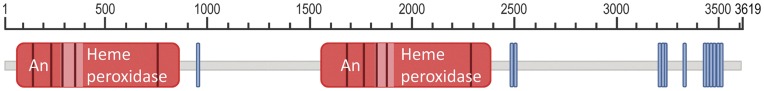
Domain architecture of PepA. Two animal peroxidase-like domains (according to PROSITE profile PS50292) are shown in red, with an internal region of low homology marked in fair red; sequences with the consensus G-X-D-G-X(5)-D-D interrupting the domains are shown in brown bars and hemolysin calcium binding motifs (according to PS0033) are in blue bars. Numbers indicate amino acid residues.

**Figure 3:**
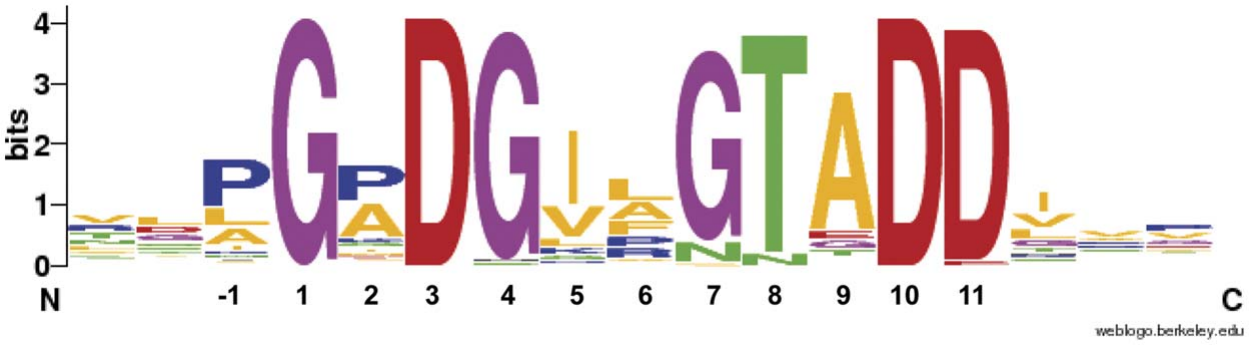
Sequence logo of insertions interrupting the ANP-like domains of bacterial proteins. The figure was generated at http://weblogo.berkeley.edu based on an alignment of 74 domain insertions matching the motif x-x-x-G-x-D-x(6)-D/E-D/E-x-x-x from 24 bacterial proteins. See Table S1 and Text S2 for more details.

A scan of the protein encoded by PP_2561 (PepA) at MyHits (http://myhits.isb-sib.ch/), which includes PeroxiBase, PROSITE and Pfam databases, categorized this protein as a member of the animal heme peroxidase superfamily. To retrieve sequences of bacterial members of this superfamily, UniProtKB (Swiss-Prot and TrEMBL, release July 13^rd^ 2010) was scanned using the PROSITE profile PS50292, which defines the animal heme peroxidase superfamily, and by applying a taxonomic filter for bacteria. One hundred and twelve sequences containing 125 hits in total were retrieved. Eight of these hits in seven sequences were excluded from further analysis due to an unusually short size of less than 200 amino acids. Interestingly twelve sequences, including three from *P. putida* (strains KT2440, F1 and GB-1) and nine from different α-proteobacteria, presented two animal peroxidase-like (ANP-like) domains within the same protein. Since these 12 protein sequences differed widely in length (between 2650 and 3619 amino acids), we decided to compare their 24 catalytic domains (matching profile PS50292) instead of the complete protein sequences. A sequence alignment of these ANP-like domains showed for some sequences multiple insertions (Fig. S1). In the case of the three *P. putida* homologues, 3 insertions were initially detected in each of the 6 domains. Most interestingly, an alignment of these 18 insert sequences revealed that they share significant sequence similarities, which are defined by the consensus G-x-D-G-x(2)-G-T/N-A-D-D (Text S1). An example of these insertions is shown in figure 1. Visual inspection of the sequences indicated that there are further sequence segments, which show a high degree of similarity to the above consensus. These additional fragments were recognized when a less stringent motif G-x-D-x(6)-D-D was used for the sequence scan. Analysis of the three *P. putida* proteins with this less stringent motif resulted in the detection of 10 inserts per sequence. The consensus obtained from these 30 inserts identified for the *P. putida* sequences differed only slightly from the initial one as Thr appeared besides Ala in the ninth position of the consensus (Table 2; Text S1). Interestingly, all these insertions were located within the two ANP-like domains of the proteins (Table S1). A scheme with the architecture of PP_2561 containing these insertions is shown in figure 2. A total of 119 hits within 27 protein sequences presenting the motif G-x-D-x(6)-D/E-D/E were retrieved from all 105 bacterial heme-dependent peroxidase sequences (Table 2; Table S1). Among these were the 12 sequences that contain two ANP-like domains and, in addition, 15 sequences that harbor a single ANP-like domain. Interestingly, 71 of these hits presented a Gly in the forth position and, with the exception of one hit, all were located within the peroxidase domain (Table 2). In these 70 hits in 23 sequences the tenth residue was always Asp. The negative charge of the side chain was also fully conserved for position 11 of the motif, since aspartate and in two cases glutamate residues were found at this position. In contrast, from the remaining 48 inserts, which do not present Gly in the forth residue, only 4 are found within the ANP-like domain and the rest are in other parts of the protein. Thus in total 74 inserts within the ANP-like domains of 24 protein sequences match G-x-D-x(6)-D/E-D/E (Table 2; Table S1). A subsequent alignment of such inserts (Text S2) resulted in the web logo of figure 3. Ninety one percent of these insertions match G-x-D-G-x(5)-D-D, of which all except one present G-x-D-G-x-x-G/N-T/N-x-D-D and are contained in 23 protein sequences (Table 2; Table S1). Of these, 12 possess two ANP-like domains and 11 others only one domain (Table 3; Table S1).

**Table 2:** Sequence variation of the eleven residues inserts found in the animal heme dependent peroxidases of bacteria.

Bacteria	Sequence^a^	Intra ANP-like domain hits	Extradomain hits	Number of ANP-like domains	Number of sequences
*P. putida* (3)					
	G-x-D-x(6)-D/E-D/E	30	3	6	3
	G-x-D-x(6)-D-D	30	0	6	3
	G-x-D-G-x(5)-D-D	30	0	6	3
	G-x-D-G-x(2)-G-T/N-A/T-D-D	30	0	6	3
	G-x-D-G-x(2)-G-T-A-D-D	24	0	6	3
	G-x-D-{G}-x(5)-D-D	0	3	6	6
All (105)					
	G-x-D-x(6)-D/E-D/E	74	45	39	27
	G-x-D-G-x(5)-D/E-D/E	70	1	35	23
	G-x-D-G-x(5)-D-D/E	70	1	35	23
	G-x-D-G-x(5)-D-D	68	1	35	23
	G-x-D-G-x(2)-G/N-T/N-x-D-D^b^	67	1	35	23
	G-x-D-G-x(2)-G/N-T/N-A/T-D-D	58	1	35	23
	G-x-D-G-x(2)-G-T-A-D-D	42	1	35	23
	G-x-D-{G}-x(5)-D/E-D/E	4	44	4	4

a {G} any amino acid except Gly.

b This sequence is PERCAL motif (see the last section of the Results).

Those sequences with Gly in the fourth position are shaded.

**Table 3:** Bacterial heme peroxidases containing G-x-D-G-x(5)-D-D insertions within the ANP-like domain as defined by the PS50292 profile.

Bacteria	Uniprot code	Length (aa)	Number of ANP-like domains	Number of insertions^a^
**α-proteobacteria**				
*Erythrobacter* sp. SD-21	A5PER4	2138	1	3
*Fulvimarina pelagi* HTCC2506	Q0G341	2650	2	2+0^b^
*Aurantimonas manganoxydans* SI85-9A1	Q1YMS2	3297	2	3+0
**β-proteobacteria**				
*Nitrosomonas* sp. AL212	C6MFN2	1639	1	1
**γ-proteobacteria**				
*Pseudomonas putida*				
F1	A5W572	3619	2	5+5
GB-1	B0KJL7	3618	2	5+5
KT2440	Q88JT6	3619	2	5+5
**δ-proteobacteria**				
*Leptothrix cholodnii*				
ATCC 51168 / LMG 8142 / SP-6	B1Y442	1650	1	2
*Mesorhizobium* sp. BNC1	Q11K84	2950	1	3
*Methylobacterium chloromethanicum*				
CM4 / NCIMB 13688	B7KRS2	2342	1	2
CM4 / NCIMB 13688	B7KW13	3587	2	2+0
CM4 / NCIMB 13688	B7L1G6	2342	1	1
*Methylobacterium extorquens*				
DSM 5838 / DM4	C7CGY0	3618	2	2+0
PA1	A9W3A5	3587	2	2+0
*Rhodopseudomonas palustris*				
BisA53	Q07SX1	3113	2	2+0
BisB5	Q13AU2	3094	2	2+0
*Roseobacter* sp.				
AzwK-3b	A6FKA5	1706	1	1
AzwK-3b	A6FV45	2197	1	1
MED193	A3XF15	3377	2	1+0
*Roseovarius* sp. TM1035	A6E280	3045	2	4+0
**Actinobacteria**				
*Arthrobacter chlorophenolicus*				
A6 / ATCC 700700	B8HDW8	1712	1	2
*Arthrobacter* sp. FB24	A0JUB7	1625	1	1
**Cyanobacteria**				
*Cyanobium* sp. PCC 7001	B5IMQ8	2168	1	1

a The number of insertions per domain is shown.

b The second domain is incomplete.

Interestingly, in all bacterial proteins with two ANP-like domains, this motif was present in the N-terminal domain, with the exception of *P. putida* sequences, which presented insertions also in the C-terminal domain (Table 3; Table S1).

### Phylogenetic Analysis of ANP-like Domains of Bacterial Proteins with at Least One Intradomain Hit with the Motif G-x-D-G-x(5)-D-D

**Figure 4:**
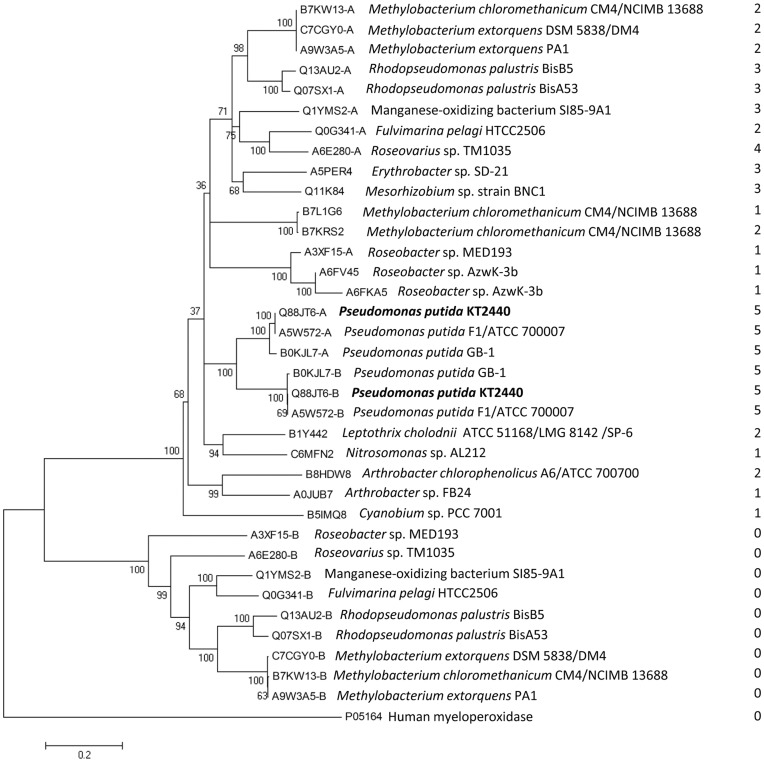
Neighbor-joining phylogenetic tree of bacterial ANP-like domains contained in animal heme peroxidases with G-x-D-G-x(5)-D-D. The domain of human myeloperoxidase, which does not contain any hit, is included as an out-group member. The alignment shown in figure S2 was cut at the position which corresponds to the sequence boundaries of the human myeloperoxidase domain. The tree is based on this reduced alignment. The bootstrap consensus tree inferred from 500 replicates is shown. Letters –A and -B refer to N- and C-terminal ANP-like domains, respectively. All the 23 sequences here considered presented at least one insert with the consensus of PERCAL (G-x-D-G-x(2)-G/N-T/N-x-D-D) (Table S1). The column on the right indicates the number of insertions in each of the domains. Bar: 0.2 substitutions per amino acid position.

The 23 full-length protein sequences that contain at least one G-x-D-G-x(5)-D-D motif harbor in total 35 ANP-like domains. A significant number of ANP-like domains contained one of these motifs inserted close to the C-terminal end (Table S1) and the sequences flanking the C-terminal extension of the domains were included to optimize the alignment of this region (Text S3). The phylogenetic tree derived from this alignment (Fig. S2) shows two major branches (Fig. 4). One of them groups the ANP-like domains contained in single domain ANHEMPs with the N-terminal domain of two-domain ANHEMPs, and the other branch groups all the C-terminal ANP-like domains that are free of inserts. N- and C-terminal ANP-like domains clustered in the same branch only in the three *P. putida* strains (Fig. 4). This suggests that there may have been two evolutionary mechanisms leading to the formation of two-domain ANHEMPs, the duplication of the same domain (applies for *P. putida*) and the recruitment of two unrelated ANP-like domains in a single protein (the rest).

### Search of the Protein Data Bank for Structures which Contain the Consensus Motif Identified

**Figure 5:**
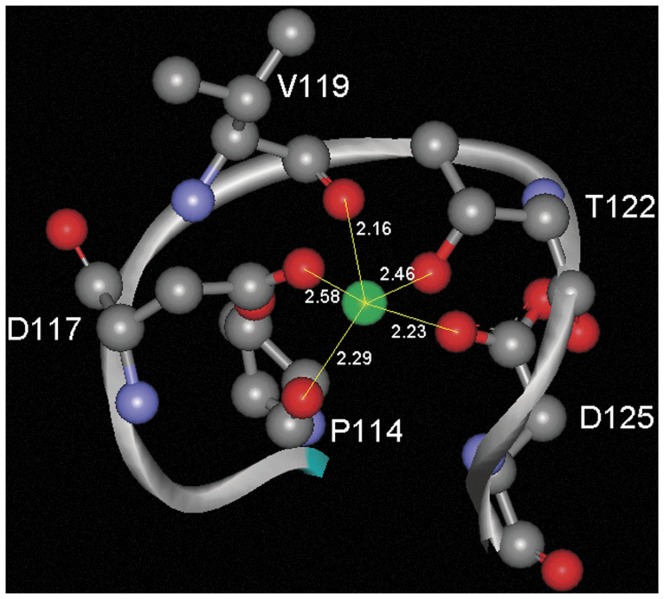
Zoom at the calcium-binding region of the pseudopilin structure. The amino acids which establish contacts with the calcium ion are shown in ball-and-stick mode and the corresponding distances are indicated (in Å). The structure is deposited at the protein data bank under the code 3G20. The figure was produced using the program WebLabViewer (http://www.marcsaric.de/index.php/WebLab_Viewer_Lite).

The sequences of the protein data bank (pdb) were searched with the motif G-x-D-G-x(5)-D-D. At the time of the search there were 71138 entries in the pdb. In total 6 protein structures were identified which contain this sequence motif. These structures were a lignin peroxidase (pdb 1B80), an acetate kinase (pdb 1G99), an amine dehydrogenase (pdb 1JJU), a domain of unknown function (pdb 2I6E) and two different pseudopilin structures (pdb 1T92 and 3G20). Figure S3 highlights the segments corresponding to the sequence motifs in these 6 three dimensional structures. In 5 of these structures the motif is surface exposed and forms a loop structure. In 4 of these structures the motif is not involved in the recognition of any ligands. However, in the structures of the lignin peroxidase and pseudopilin (pdb 3G20), this motif was involved in the coordination of a calcium ion and the molecular details of these interactions in the case of pseudopilin is shown in figure 5.

In the pseudopilin structure [19] the sequence motif is present on the fragment comprising amino acids 114 to 125 with the sequence P**G**P**DG**VPNTE**DD** (amino acids of the motif are in bold). As shown in figure 5 the bound calcium ion is pentacoordinated by two main chain interactions (P114 and V119) as well as by three side chain interactions (D117, T122 and D125). The aspartate residues 117 and 125 are part of the sequence motif. No calcium ions were present in buffers used for the purification and crystallization of the protein, which suggests that bound calcium has been co-purified with the protein indicative of a high binding affinity. In the case of the lignin peroxidase structure [20] the bound calcium is coordinated by two interactions with main chain oxy groups, three side chain interactions and two water molecules. From the motif identified only the first glycine (main chain interaction) and the first aspartate residue (side chain) are involved in the coordination of the calcium ion. The C-terminal pairs of aspartate residues are not involved in calcium coordination.

Structural data strongly suggest that calcium is bound at the consensus motifs present in the lignin peroxidase (pdb ID 1B80) and the pseudopilin (pdb ID 3G20) structures. Both structures [19], [20] have been solved at resolution inferior to 1.8 Å and were refined to R values of 0.17 and R_free_ values of 0. 21–0.22 %. These parameters minimize the probability of a misinterpretation of the final electron density maps. In addition there is functional evidence for a role of calcium in the activity of both proteins. Korotkov and colleagues [19] demonstrated that the activity of pseudopilin, key features of type II secretion systems, depends on the presence of bound calcium. Nie and Aust [21] have shown that calcium ions are released from lignin peroxidase following its thermal denaturation. The same authors were able to correlate the loss of calcium with a loss of enzymatic activity [22].

### Design of the BACHEMP-Cons Peptide Which Harbors the Consensus Sequence of the Motif Identified

Based on the above observation, we hypothesized that the sequence motif identified might correspond to another, yet unidentified binding motif for calcium ions. To verify this hypothesis experimentally and to determine precisely the ligand specificity of this motif we designed a peptide for subsequent microcalorimetric titrations with different cations. Due to the flexible nature of the peptide main chain, peptides can adopt many different conformations. However, the inspection of the pseudopilin structure reveals that the amino acids of the motif need to be present in a defined loop structure in order to establish the contacts detailed in figure 5. This structure also shows that the segments before and after the motif, two beta strands, interact with each other in an antiparallel manner. For that reason we hypothesized, that this antiparallel interaction between the strands flanking the motif is important for the adaption of the correct conformation of the motif.

**Figure 6:**
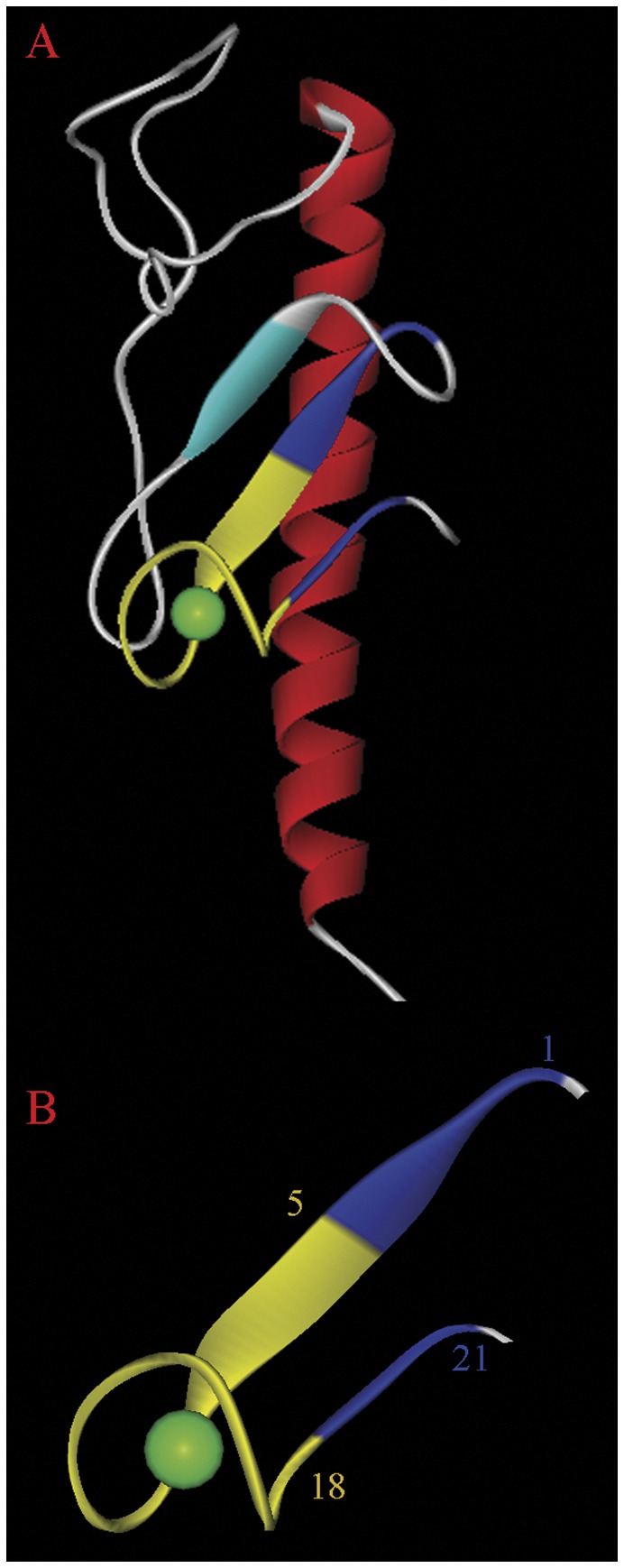
Structural basis for the design of the BACHEMP-Cons peptide. (A) Three dimensional structure of *E. coli* pseudopilin (pdb 3G20). Bound calcium is shown in green. The fragment which forms the basis for the design of peptide BACHEMP-Cons is shown in blue and gold. The BACHEMP-Cons peptide is composed of amino acids from this structure (shown in blue) and amino acids that correspond to the consensus defined (shown in gold). (B) The fragment of the structure that corresponds to the BACHEMP-Cons peptide. The sequence of the peptide is colored according to the color coding explained above. The figure was produced using the program WebLabViewer (http://www.marcsaric.de/index.php/WebLab_Viewer_Lite).

We therefore designed a 21-mer peptide, termed BACHEMP-Cons, in which the first 4 (DIDI) and the last 3 amino acids (IGN) correspond to the flanking beta strands in the pseudopilin structure. These sections are colored in blue in figure 6. The sequence of the fragment of the pseudopilin structure that is involved in Ca^2+^ binding was replaced by the consensus sequence derived from the inserts within the ANP-like domains of 24 bacterial proteins (Fig. 3). This consensus sequence is shown in gold in figure 6. The final sequence of the BACHEMP-Cons peptide is thus DIDIVLPGPDGILGTADDIGN. This peptide was synthesized and submitted to microcalorimetric titrations.

### Experimental Proof for the Binding of Calcium Ions to the Motif Identified

**Figure 7:**
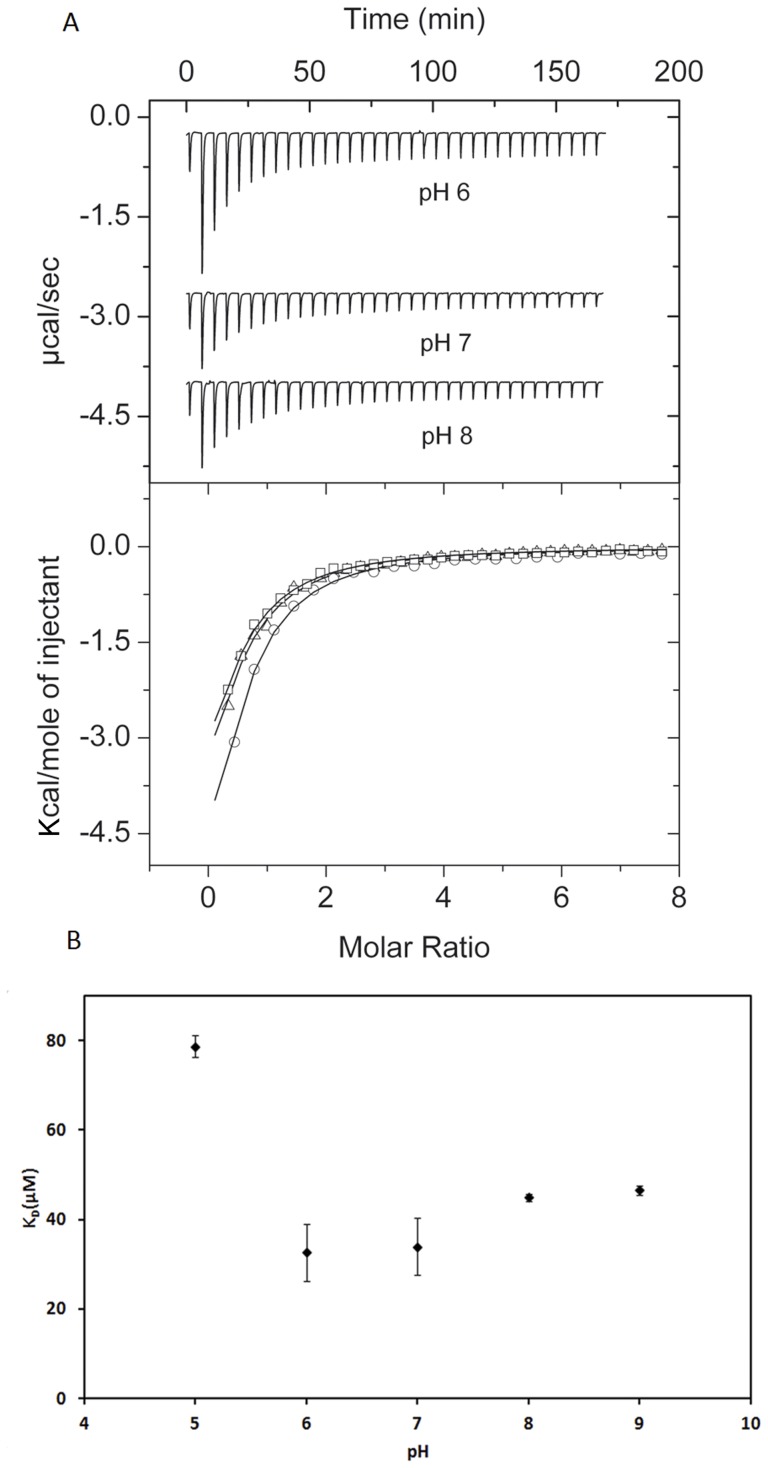
Isothermal titration calorimetry studies of the interaction of the BACHEMP-Cons peptide with CaCl_2_. (A) Upper panel: raw data for the titration of 50 µM peptide with 3.2 µl aliquots of 5 mM CaCl_2_. Experiments were conducted in polybuffer at the pH values indicated. Lower panel: Integrated, dilution-corrected and concentration-normalized raw data. Data were fitted with the “One binding site model” of the MicroCal (Northampton, MA) version of ORIGIN. pH 6.0 (○), pH 7.0 (□), pH 8.0 (Δ). The derived thermodynamic parameters are given in Table 4. (B) Dependence of *K*_D_ on the pH. Experiments were conducted in polybuffer which was adjusted to the pH indicated by the addition of concentrated HCl or NaOH. Shown are means and standard errors derived from three individual experiments.

Isothermal titration calorimetry (ITC) [23] was used to study the interaction between the peptide BACHEMP-Cons and various ligands. Based on the observation that there are various protein structures in which calcium ions are bound to the sequence motif identified, initial experiments were aimed at investigating whether the peptide designed was able to bind Ca^2+^. Initially polybuffer (pH 6) was titrated with 5 mM CaCl_2_ to assess the dilution heat effects. Peaks resulting from this titration were small and uniform (not shown), indicative of weak dilution heats. Subsequently 50 µM peptide were titrated with CaCl_2_. Significant exothermic heat changes were observed which diminished as the titration proceeded (Fig. 7A), upper trace. These heat changes are due to binding events at the peptide. Data analysis revealed that binding was driven by favorable enthalpy (−16.3±8.3 kcal/mol) and counterbalanced by unfavorable entropy changes (*TΔS* = −10.2±8.4 kcal/mol). An association constant of 30600±10000 M^−1^ was determined which corresponds to a dissociation constant *K*_D_ of 33±11 µM.

This binding event could have been caused by the interaction of the Ca^2+^ cation or the Cl^−^ anion with the peptide. To verify this issue the titration was repeated using 5 mM NaCl as ligand. However, no binding heats were measured (not shown), which indicates, firstly, that the above binding parameters represent the interaction of the Ca^2+^ cation with the peptide and, secondly, that the Na^+^ cation does not bind to the peptide. Subsequently the interaction of the peptide with Ca^2+^ was studied at other pH values (Fig. 7, Table 4). At all pH values binding was observed and a plot of the *K*_D_ values determined as a function of pH is shown in figure 7B. Since the pH optimum for binding was found to be at pH 6.0, all subsequent studies were conducted at this pH.

**Table 4:** Thermodynamic parameters derived from the microcalorimetric titrations of peptide BACHEMP-Cons in different buffer systems.

Ligand	Buffer^a^	Δ*H*	*T*Δ*S*	*K_D_* (µM*)*	*K_A_* (M^−1^)
		(kcal/mol)	(kcal/mol)		
CaCl_2_	PB, pH 5	−12.6±3.2	−7.01±3.2	79±4	(1.27±0.1)×10^4^
CaCl_2_	PB, pH 6	−16.3±8.3	−10.2±8.4	33±11	(3.06±1)×10^4^
CaCl_2_	PB, pH 7	−13.7±2.6	−7.59±2.6	34±11	(2.94±0.9)×10^4^
CaCl_2_	PB, pH 8	−11.2±1.6	−5.25±1.6	45±1	(2.22±0.1)×10^4^
CaCl_2_	PB, pH 9	−8.46±0.9	−2.55±0.9	46±1	(2.15±0.1)×10^4^
CaCl_2_	PB, pH 6, 100 mM NaCl	−4.53±1.5	0.92±1.2	101±11	(0.99±0.1)×10^4^
NaCl	PB, pH 6, 100 mM NaCl	No binding^b^			
MgCl_2_	PB, pH 6, 100 mM NaCl	No binding^b^			
CuCl_2_	PB, pH 6, 100 mM NaCl	No binding^b^			
FeCl_2_	PB, pH 6, 100 mM NaCl	No binding^b^			
CrCl_2_	PB, pH 6, 100 mM NaCl	No binding^b^			
MnCl_2_	PB, pH 6, 100 mM NaCl	No binding^b^			

The values shown are means and standard deviations from three individual experiments.

a PB, Polybuffer (5 mM Tris, 5 mM MES, 5 mM PIPES).

b Under the experimental conditions used (titration of 50 µM peptide with 5 mM salt solution), which corresponds to a final ligand concentration in the sample cell of 900 µM.

**Figure 8:**
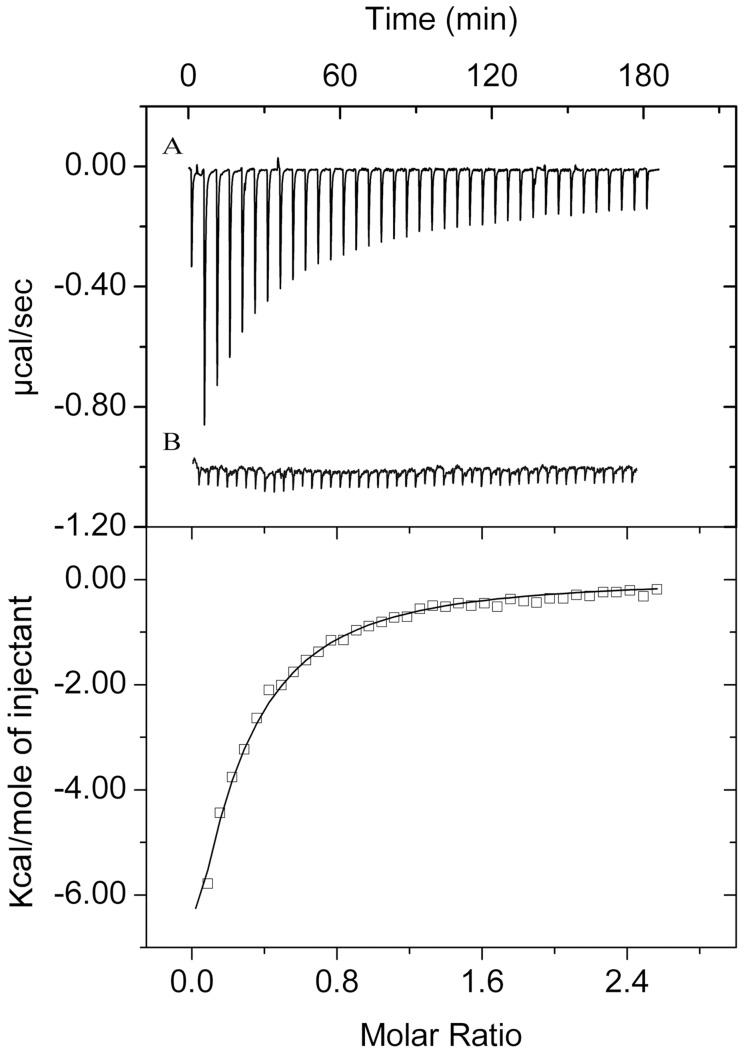
Isothermal titration calorimetry studies to evaluate the specificity of molecular recognition between the peptide BACHEMP-Cons and cations. Upper panel: Raw data for the titration of 50 µM peptide with 3.2 µl aliquots of CaCl_2_ (A) and MgCl_2_ (B) Experiments were conducted in polybuffer pH 6.0 supplemented with 100 mM NaCl. Lower panel: Integrated, dilution-corrected and concentration normalized titration data of the peptide with CaCl_2_. Data were fitted with the “One binding site model” of the MicroCal (Northampton, MA) version of ORIGIN. The derived thermodynamic parameters are given in Table 4.

The next experiment was aimed at verifying whether or to what degree the presence of monovalent cations interferes with the Ca^2+^ recognition by the BACHEMP-Cons peptide. To this end the titration of the peptide with CaCl_2_ was repeated in polybuffer supplemented with 100 mM NaCl (Fig. 8, Table 4). Data analysis revealed a reduction of affinity by a factor of around 3 (*K*_D_ = 102±11 µM) and a change in the mode of binding, since binding was driven by favorable enthalpy and entropy changes. However, data illustrate that this interaction also occurs in the presence of physiologic concentrations of monovalent cations like Na^+^.

### Experimental Evidence that the Motif Identified is Specific for Calcium Ions

A central question in the study of the motif described concerns the specificity of ligand recognition. To evaluate the specificity of binding, microcalorimetric titrations of the peptide in polybuffer, pH 6.0, supplemented with 100 mM NaCl were conducted using chloride salts of Mg^2+^, Cu^2+^, Fe^2+^, Cr^2+^ and Mn^2+^. For none of these metal ions was a binding heat detected. This finding is exemplified by the titration of the BACHEMP-Cons peptide with MgCl_2_ as shown in figure 8B. The observed heats were small and uniform, matching those observed for the titration of buffer with this ligand.

In summary, the microcalorimetric titrations reveal, firstly, that Ca^2+^ binds with a physiologically relevant affinity to BACHEMP-Cons, secondly, that this binding is specific and, thirdly, that binding also occurs in the presence of physiological concentrations of monovalent anions and cations.

### Experimental Proof for the Binding of Calcium Ions to the N-terminal ANP-like of the PP_2561 Encoded Protein

To study the interaction of Ca^2+^ with the protein encoded by PP_2561, the DNA fragment coding for the N-terminal ANP-like domain was cloned in the expression vector pET28 and the resulting his-tagged fusion protein was expressed in *E. coli*. No hemolysin type calcium binding sites are present in this domain (Fig. 2). Spectral analysis of the purified protein revealed the absence of bound heme, the co-factor essential for catalytic activity. Therefore protein was reconstituted with heme (see materials and methods for details) and spectra of the reconstituted protein were recorded (Fig. S4). From the absorbance at 280 nm and 410 nm a molar ratio heme/protein of 1∶0.9 was determined. Subsequently, protein was submitted to spectrophotometric assays to measure its peroxidase activity. Using the conditions described under Materials and Methods a *k*_cat_ of 29±9 min^−1^ and *K*_M_ of 13±1 mM for peroxide were calculated. These results confirm the annotation of this domain as peroxidase. Since we were able to detect the protein on the bacterial surface (unpublished), the protein encoded by PP_2561 was renamed as PepA (*Pseudomonas* extracellular peroxidase).

**Figure 9:**
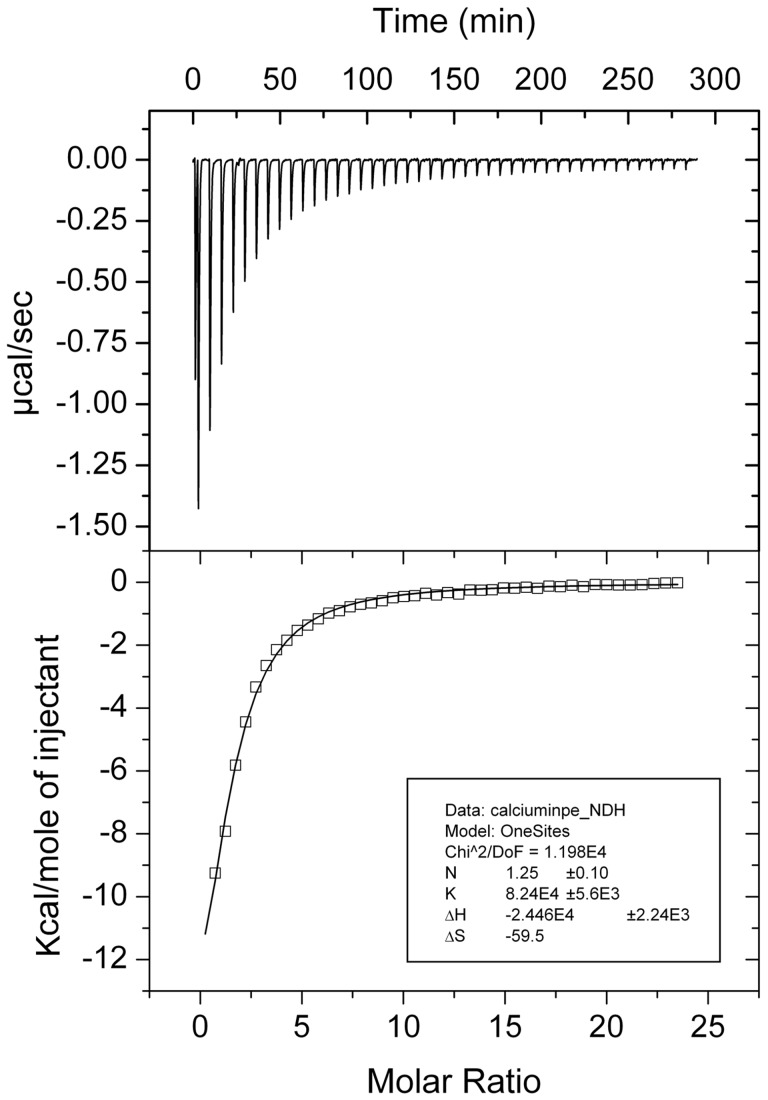
ITC analysis of Ca binding to the N-terminal ANP-like domain of PepA. Upper panel: Raw titration data for the injection of 6.4 µl aliquots of 1 mM CaCl_2_ into 11.2 µl of recombinant protein. Ligand and protein were in buffer Tris-HCl 10 mM, NaCl 50 mM, Glycerol 10%, pH 7.5. Experiments were carried out at 25°C. Lower panel: Integrated, dilution-corrected and concentration-normalized peak areas of titration raw data. Shown is the fit with the “one binding site model” of the MicroCal version of ORIGIN.

Subsequently, the binding of Ca^2+^ to the purified domain of PepA was analyzed using ITC. To avoid that the protein sample contains bound Ca^2+^, the protein was purified under denaturing conditions and then refolded (see Materials and Methods for details). The microcalorimetric titration of the resulting protein with CaCl_2_ is shown in figure 9. The titration of the protein resulted in large exothermic signals, indicative of enthalpy driven binding. Data analysis gave rise to a dissociation constant of 12±1 µM and an enthalpy change of −24.5±2 kcal/mol. These data show that calcium binding also occurs to the PERCAL motif containing catalytic domain. To assess a potential influence of calcium binding on the catalytic activity, protein at the same concentration as used for the ITC experiments (11 µM) was exposed to calcium concentration in the range between 3.6–30 µM. Samples should correspond thus to protein differentially saturated with calcium. However, the catalytic activities of these samples were almost indistinguishable, indicating that calcium binding has no modulatory effect on the catalytic activities.

### Frequency of the Consensus Sequence G-x-D-G-x(5)-D-D and Four of its Variants in the Databases

The totality of sequences present in the UniProtKB/TrEMBL database were screened using PROSITE for the presence of the identified Ca^2+^ binding motif and derivatives thereof (Table 5). To this end the sequences from the individual kingdoms of life were analyzed separately. The observed number of hits was then compared to the estimated number of expected random matches for the corresponding motif, which was calculated using the algorithm described by Nicodème [24]. The ratio of the observed number of hits over its calculated random occurrence can provide first information on the phylogenetic distribution of these motifs (Table 5, Table S2). Motifs 1 and 2 (Table 5) covered more than 90% of the insertions within the ANP-like domains, although the ratio to the random occurrence of motif 1was only slightly above 1. This in addition to the results shown in table 2 suggests that this particular motif is little specific. The ratio raised significantly for motif 2, in which the stringency of positions seven and eight was increased. This was more noteworthy in the bacterial kingdom, for which the ratio increased to around ten. It should be mentioned that the core of the BACHEMP-Cons peptide matches motif 2. Thus we propose G-x-D-G-x-x-[GN]-[TN]-x-D-D as a new Ca^2+^ binding motif named PERCAL (of peroxidase and Ca binding). The database was also scanned against motif 3, which presents glutamic acid in the ninth position and covers 82% of the ANP-like intradomain insertions plus the sequence found in 122 pseudopilin sequences including that of the structure with pdb 3G20. In this case the ratio to the random occurrence increased to 26, as a consequence of the elevated value obtained for bacteria (41) though the ratio in eukaryotes was lower than 1. The same search was performed with motif 4, which presents aspartic acid in this ninth position since it was observed in 15 pseudopilin sequences. Interestingly the ratio of hits observed with motif 4 in comparison to the estimated random occurrence increased to 34 in eukaryotes. Motif 5 is the result of restricting the ninth residue to A, T, D or E in motif 2. The ratio calculated for this motif, which includes more than 80% of insertions in peroxidases and the sequences found in pseudopilins, was in all kingdoms of life significantly superior to 1 (Table 5, Table S2). The core of BACHEMP-Cons peptide also matches this motif. The fact that the ratios of hits using the motif and its derivatives is significantly above the number of random hits expected suggests that a significant number of proteins in bacteria as well as in eukaryotes have evolved to posses PERCAL motifs. The different ratios of specific over random hits in bacteria and eukaryotes might suggest that the motif may further be present in different sub-families. However, experimental data are essential to verify the functional role of PERCAL motifs in other proteins.

**Table 5:** Presence of the different versions of the PERCAL calcium binding motif in bacteria and eukaryotes.

Motif Number	Motif sequence	Coverage of insertions^(a)^	Ratio of observed sequence motifs over estimated random occurrence		
			All^(b)^	Bacteria^(c)^	Eukaryota^(d)^
1	G-x-D-G-x(5)-D-D	91	1.54	1.14	2.67
2	G-x-D-G-x-x-[GN]-[TN]-x-D-D (PERCAL)	90	8.12	10.59	3.51
3	G-x-D-G-x-x-[GN]-[TN]-[AET]-D-D	82.43	26.58	41.35	0.91
4	G-x-D-G-x-x-[GN]-[TN]-D-D-D	1.35	17.68	11.73	34.48
5	G-x-D-G-x-x-[GN]-[TN]-[ADET]-D-D	83.78	24.70	35.15	7.93

An expanded table with more motifs and the details required for the generation of this table are reported in Table S2.

a Percentage of insertions (of a total of 74) within ANP-like domains recognized by the corresponding motif.

b Number of total entries in the database 18215214

c Entries from Bacteria (63%)

d Entries from Eukaryota (28%)

## Discussion

Previous work in our laboratory revealed the importance of the bacterial determinant PP_2561 in the beneficial interaction that the bacteria *P. putida* KT2440 establish with the model plant Arabidopsis [12]. The initial annotation of the PP_2561 encoded protein was a putative secreted hemolysin-type calcium-binding bacteriocin (EMBL AAN68170.1). In the course of our investigation it was renamed as a heme peroxidase that we have called PepA. Inspection of the PepA entry at the Conserved Domains Database [25] revealed that it consists of two ANP-like domains (as defined by profile PS50292 of PROSITE) with an internal region of low homology. In addition PepA contains at its C-terminal end a region that shares sequence similarities with the peptidase M10 serralysin. This fragment contains multiple calcium binding motifs defined by Pfam00353. PepA not only presents Ca-binding sites concentrated at its C-terminal end as part of the serralysin-like C-terminal domain but also the hemolysin-type calcium-binding (HTCaB) motifs (signature PS00330) on the segment flanking the C-terminus of both ANP-like domains (Fig. 2). Given that the sequence fragments recognized by profiles PS50292 and PS00330 did not overlap the region of low homology observed within the ANP-like domains, it can be concluded that they were not due to these well characterized HTCaB signatures. In addition, a distinctive feature of the ANP-like domains of PepA was the presence of short insertions of eleven residues, which were conserved in the domains of their *P. putida* homologues (strains F1 and GB1) and in other homologous proteins from diverse bacterial taxons (Table 3). The sequence analysis of these inserts in the ANP-like domains resulted in the definition of an initial motif G-x-D-G-x(5)-D-D, which was used for searches in the pdb database. Interestingly, one of the 6 entries identified (pseudopilin) contained a hit with the sequence G-p-D-G-v-p-N-T-e-D-D involved in the coordination of a calcium ion (Fig. 5), which also matches the more specific consensus sequence G-x-D-G-x-x-[GN]-[TN]-x-D-D proposed in this work as PERCAL. Given that pseudopilin is not predicted by PROSITE, Pfam and SPRINT to possess a calcium binding motif, experiments were conducted to determine whether the consensus defined in the figure 3 binds calcium ions. Microcalorimetry was employed to study ligand binding to the BACHEMP-Cons peptide that contained a motif sequence matching this consensus. Binding occurred over the pH range tested (pH 5–9) with dissociation constants ranging from 33–79 µM. This value is well above the intracellular calcium concentration, which in *E. coli* is approximately 90 nM [26]. However, the affinity of calcium binding proteins for Ca^2+^ ranges from submicromolar to millimolar dissociation constants [6], [27], [28]. These differences in affinity may reflect the differences in the calcium levels present in the protein environment. Due to the relatively low cellular Ca^2+^ concentration, cytosolic proteins may have evolved to recognize calcium more tightly than extracellular proteins. It should be mentioned that the niche for *P. putida* is garden soil and the plant rhizosphere [29]. The exchangeable Ca^2+^ concentration (ECC) in soil varies considerably but even for a soil containing a low ECC of 20 mmol/L [30], this value is significantly higher than the above mentioned cytosolic concentration. In this context a dissociation constant of 12 µM, as obtained for the N-terminal peroxidase domain of PepA, would result in a calcium saturation of the protein.

Ca^2+^ binding to the BACHEMP-Cons peptide also occurred, although with reduced affinity, in the presence of physiologic concentrations of monovalent ions. Most importantly, no binding of other bivalent cations was observed, which demonstrates that the PERCAL motif is specific for calcium. Calcium binding occurred also to the recombinant purified N-terminal peroxidase domain of PepA with an affinity superior to the values observed for the peptide. Similarly to our results, an increase in Ca^+2^ binding affinity to the protein with respect to different peptides have also been observed [31]. It should be noted that the stoichiometry of calcium binding was found to vary considerably between the individual protein lots. This may be due to technical difficulties to generate protein entirely devoid of calcium, in spite of having used reagents with the lowest calcium traces to prepare solutions for protein refolding. We therefore have to consider the protein used for ITC binding studies in figure 9 as partially Ca^2+^ saturated. It is tempting to speculate that, high affinity PERCAL sites are already calcium saturated in the samples used, which consequently implies that ITC permitted only the observation of the lower affinity calcium binding events.

The consensus proposed for PERCAL corresponds to motif 2 in table 5. This motif recognizes 90 % of the inserts in the ANP-like domains. In addition, its observed frequency is for bacteria as well as for eukaryotes above the expected value for a random distribution. The ratio of the observed hits over its estimated random quantity was particularly elevated in prokaryota, which suggested that the Ca^2+^ binding motif here identified is particularly frequent in this kingdom (Table 5; Table S2). A similar result was obtained for several sequences included in versions of the PERCAL motif, i.e motif 5 of Table 5, which has the ninth position of the consensus restricted to the four amino acids Ala, Asp, Glu and Thr. In this case the ratios increased up to about 8 in eukaryotes and 35 in prokaryotes, which is consistent with the proposition that PERCAL is an omnipresent new Ca^2+^ binding motif. The ninth position of PERCAL deserves an additional attention since residues at this position appears to be specific for individual protein families: 1) in ANP-like domains of bacterial proteins PERCAL habitually presents A/T in this position (86%); 2) in the general secretion pathway protein G (or pseudopilin) [32], this position is in all cases occupied by D/E and 3) when this position is occupied by aspartate, as for motif 4 of Table 5, the resulting motif, identified in uncharacterized proteins and proteins containing a Zn finger domain elsewhere, is 34 times more frequent than randomly expected in eukaryotes. However, we have maintained an ambiguity in the ninth position of this consensus sequence although motif 5 (G-x-D-G-x-x-[GN]-[TN]-[ATDE]-D-D) was over represented in the databank compared to PERCAL. This decision was taken since the introduction of a restriction at this position would have prevented the detection from more than half of the eukaryotic hits identified with PERCAL (Table S2). In addition, a restriction of position 9 would also have resulted in the detection of fewer insertions in the ANP-like domains. We conclude that each of these insertions, five in every ANP-like domain of PepA, would be able to bind calcium. Further knowledge on the structures of proteins containing PERCAL, especially eukaryotic proteins, is needed in order to identify residues that are critical for binding and that consequently should be playing a central role in the precise definition of the PERCAL motif.

The analysis of the pseudopilin structure provides insight into the molecular basis of the recognition of Ca^2+^ by the PERCAL motif. There are five direct interactions of which three involve main chain oxygen atoms (amino acids 1, 5 and 8). Of these, only amino acid at position 8 shows a high degree of conservation. Most interestingly, there are only two side-chain interactions (aspartates at positions 3 and 11). This may suggest that the remaining conserved amino acids of the motif have a structural role and may contribute to the formation of the loop structure. Frequently, ligand recognition is mediated by an indirect interaction with protein bound water. The pseudopilin structure has been solved at a resolution of 1.78 Å and contains 265 water molecules [19]. However, the inspection of the calcium binding site reveals the absence of bound water suggesting that water may not be involved in Ca^2+^ recognition. Striking parallels exist to other calcium binding motifs, as exemplified by the HTCaB region of the alkaline protease [4]. In analogy to PERCAL motif, Ca^2+^ establishes only two interactions with amino acid side chains. As in the case of PERCAL these side chains are aspartic acid residues. In the structure of the alkaline protease the remaining 4 interactions involve main chain oxygens, of which three are from conserved glycine residues.

We have investigated the catalytic activity of the N-terminal ANP-like domains of PepA, which contains 5 PERCAL insertions. The *K*_M_ for H_2_O_2_ of the His-tagged N-terminal domain (PepA-Nter) after its reconstitution with heme was estimated to be 13±1 mM. *K*_M_ values higher than 10 mM have been reported previously for the oxidation of 4-aminoantipyrine (same electron donor than the one used in this study) by H_2_O_2_ in the presence of cytochrome c [33]. There is a wide range in the kinetic constants of peroxidases. As an example *k*_cat_ values for myoglobin and horseradish perodixase vary as much as three orders of magnitude (21 and 60000 min^−1^, respectively) [34]. The work performed here with PepA constitutes to our knowledge the first testimony in the literature reporting kinetic parameters on bacterial heme peroxidases that are included in the animal peroxidase_like superfamily. It should be mentioned that no peroxidase activity was observed for PepA using an alternative assay that employs a different electron donor [35]. This suggests that the nature of the electron donor is decisive to measure its catalytic activities. However, the physiologically relevant electron donor of PepA remains to be identified. Another possible hypothesis to explain the low peroxidase activity of PepA-Nter may be the reduction of enzymatic activity caused by the insertion of PERCAL motifs although further work with homolog proteins is required to assess whether these catalytic properties are conserved.

The binding of calcium to pseudopilin was found to be essential for protein activity [19]. To verify whether Ca^2+^ binding modules PepA activity, the peroxidase assays were conducted using heme-reconstituted PepA-Nter differentially saturated with Ca^2+^. The catalytic properties of these differentially Ca^2+^ saturated proteins did not reveal any major variations, but considering the low magnitude of response a more subtle Ca-mediated modulation of peroxidase activity cannot be excluded. Hence it is plausible that the protein was partially saturated with its ligand previous to the incubation with Ca^2+^, given the difficulties to generate protein entirely devoid of calcium.

The analysis of the consensus sequences of known calcium binding motifs (Table 1) shows that the HTCaB motif is the closest relative of the PERCAL motif. In total PepA is predicted to contain 13 Ca-binding sites of the HTCaB type and 10 of the PERCAL type. As stated above the PERCAL sites are exclusively present on the ANP-like domains whereas the HTCaB sites are solely present on the segment located C-terminal to both ANP-like domains. PERCAL mediated plant root surface binding of PepA and a detoxification of reactive oxygen species caused by the peroxidase activity of PepA may be key mechanisms for the apparent role of PepA in rhizosphere colonization and the triggering of induced resistance against phytopathogens [12]. Current work in our laboratory is being performed to elucidate whether this protein is involved in Ca^2+^-mediated cell-cell adhesion, adhesion to biotic surfaces and in addition if its mechanism of action in the interkingdom communication is related with oxidative stress resistance.

The two ANP-like domains of PepA share around 70 % of sequence identity. Thus certain similarities in their catalytic properties are expected. Due to this elevated degree of sequence identity these domains clustered together in a phylogenetic tree of ANP-like domains of bacterial heme peroxidases containing PERCAL (Fig. 4). This was unique for PepA and their *P. putida* homologues and suggests that PepA is the result of a gene duplication event followed by divergent evolution. However, for the PepA homologues with two ANP-like domains from other species, their N- and C- terminal domains did not cluster together and remarkably in all cases only the N-terminal domains presented PERCAL motives. Therefore, two types of ANP-like domains can be distinguished: those with and those without PERCAL motif. This hence raises the question as to the functional differences between both types of domain. In this context the study of a PepA homologue from *Aurantimonas* [17]) can offer a work hypothesis. This protein, which has one ANP-like domain free of PERCAL inserts, was found of being able to oxidize Mn (II). Since *P. putida* GB1, which has a PepA homologue with PERCAL insertions in both ANP-like domains, lacks the Mn (II) oxidizing activity [18], it is tempting to speculate that the PERCAL-free ANP-domain of the *Aurantimonas* PepA homologue may be the cause of the catalytic activity. However it should be pointed out that in *Erythrobacter* sp. strain SD-21 the Mn-oxidizing protein contains only one ANP-like domain [17] with three PERCAL insertions (Fig. 4), which indicates that at present it is not possible to predict the catalytic properties of animal heme peroxidases of bacterial proteins. In addition, the PERCAL motif could account for the Ca^2+^-mediated modulation of the Mn (II) oxidation activity, as observed for the PepA homologue in *Aurantimonas*, besides the proposition that points to the HTCaB sites [17].

The recent work of Marchler-Bauer and coworkers [15] has led to the differentiation of two subtypes of bacterial ANP-like domains, namely An_peroxidase_bacterial_1 (cd09819) and An_peroxidase_bacterial_2 (cd09821). We could confirm that the presence or absence of PERCAL motif did not determine whether a given domain belongs to one or the other family. This is exemplified by the fact that the C-terminal domains of two heme peroxidases, Q1YMS2 and A3XF15 (both free of PERCAL), were included into the same cd09821 sequence cluster together with the N- and C-terminal domains of PepA, whereas in the tree generated here (Fig. 4) they were grouped apart in a different branch from the PepA domains. We thus propose the presence of PERCAL as a significant phylogentic factor in the so far uncharacterized bacterial family of heme peroxidases.

## Materials and Methods

### Database Searches

Initial searches were performed against UniProtKB/TrEMBL (release July 13^rd^ 2010, 11397958 entries) using the PROSITE profile PS50292 to identify proteins containing the animal peroxidase-like (ANP-like) domain. A taxonomy filter for bacteria was used. Segments recognized by profile PS50292, corresponding to the ANP-like domains, were extracted manually. This was followed by a search in a later release of UniProtKB/TrEMBL (release 16-Nov-11, 18215214 entries) using the pattern G-x-D-G-x(5)-D-D and a variety of more stringent derivatives, to determine the ratio of hits in the database compared to the estimated random occurrence. This analysis was initially performed without taxonomy filter and then repeated with taxonomy filters for Bacteria, Eukaryota, Viruses and Archaea.

### Alignment and Tree Construction

The ClustalW2 algorithm at http://www.ebi.ac.uk/Tools/msa/clustalw2/ and *MEGA* version 4 [36] were used for phylogenetic and molecular evolutionary analyses using default parameters. The joining neighbor method [37] was run 500 times to obtain the average bootstrap tree.

### Cloning, Overexpression and Purification of the N-terminal Animal Peroxidase-Like Domain of PepA

A 2625 bp-fragment encoding the N-terminal 875 amino acids of PepA (PepA-Nter) was obtained via PCR from cosmid pCSSH1 that contains the wild type *pepA* gene of *P. putida* KT2440 using primers 2561FNheI1 fw (5′-GGGCAAGCTAGCATGGCCAATTTC -3′) and FPxHinR rev (5′-AAGCTTCAGTTGATCTCGATGCCGC-3′). The DNA fragment was cloned into pGEM-T (Promega) and the resulting plasmid pSSH14 was introduced into *E. coli* DH5α. The restriction sites incorporated by the PCR primers (NheI-HindIII) were used for subsequent digestion with the same enzymes followed by cloning of the insert into pET28a (Novagen). The resulting plasmid was called pSSH15 and transformed in *E. coli* BL21 (DE3) (Stratagene). The insert and flanking region of this plasmid were verified by DNA sequencing.

*E. coli* BL21 (DE3) containing pSSH15 was grown in LB culture medium supplied with kanamycin (25 µg/mL) at 30° and 200 rpm. The induction of the protein expression took place by addition of 0.1 mM IPTG at an OD_600_ of 0.5. Cultures were maintained overnight at the same temperature and harvested by centrifugation. Since SDS-PAGE gels showed that the majority of recombinant protein was present in the insoluble fraction, protein was purified from inclusion bodies. Cells from 50 mL cultures were resuspended in 40 ml of buffer A (50 mM Tris-HCl, 150 mM NaCl, 10 mM Imidazol, 6 M of guanidine hydrochloride, pH 7.5), disrupted by French Press treatment and centrifugated for 1 h at 20000 × g.

The supernatant was filtered using a 0.45 µm syringe filter (Millipore), loaded onto a HisTrapHP column (GE Healthcare) previously equilibrated in buffer A and eluted with a 10 mM to 45 mM imidazol gradient in buffer A. The sample was then diluted to a final concentration of 0.1 mg/ml. Protein renaturation was performed by dialysis against 10 mM Tris-HCl, 50 mM NaCl, 2 M urea, 0.1 mM EGTA, 10% glycerol, pH 7.5 followed by multiple cycles of dilution with buffer 10 mM Tris-HCl, 50 mM NaCl, 10% glycerol, pH 7.5 and subsequent concentration using Amicon Ultra-4 30 K centrifugal filter (Millipore). The protein concentration was determined spectrophotometrically using an extinction coefficient of 59820 M^−1^ cm^−1^.

### Heme Binding to the N-terminal ANP-like Domain of PepA

Spectral analysis of purified PepA-Nter revealed an absence of bound heme. To reconstitute the protein with heme, PepA-Nter at 11 µM was incubated in reconstitution buffer ( 10 mM Tris-HCl, 50 mM NaCl, 10% DMSO, 10% glycerol, pH 7.5) containing 80 µM hemin (Fluka) at 5°C for 30 min. The excess of heme was removed by gel filtration using PD-10 columns (GE Healthcare), previously equilibrated with reconstitution buffer. Fractions were collected and analyzed spectrophotometrically at 280 (indicative for protein) and 410 nm (indicative for heme). The molar ratio of heme to PepA-Nterm was calculated using a molar extinction coefficient of 1×10^5^ M^−1^ cm^−1^ [38]. The fractions with maxima at both wavelengths, indicative for heme-containing protein, were pooled and submitted to enzymatic assays.

### Peroxidase Assay

The procedure described by Maehly and Chance [39] was used. An aqueous solution of phenol (0.17 M) and 4-aminoantipyrine (2.5 mM) was prepared. The assays mixture contained 450 µl of this solution and 500 µl of hydrogen peroxide solution in potassium phosphate buffer 0.2 M, pH 7.0. Then 50 µl of the purified protein at 4 µM or 50 µl of buffer 10 mM Tris-HCl, 50 mM NaCl, 10% glycerol, pH 7.5 (control) were added to the assay mixture. The samples were incubated at 25°C for 4 min and the increase in absorbance was recorded at 510 nm for 3 min using a Shimadzu UV-2401PC spectrophotometer. The measurements obtained with the protein were corrected with the buffer control. To determine *k*_cat_ and *K*_M_ the assays were carried out using hydrogen peroxide concentrations in the range of 0.25–45 mM. Catalytic constants were calculated using the extinction coefficient for the reaction product quinoneimine, which is 6.58 mM^−1 ^cm^−1^ [40].

### Isothermal Titration Calorimetry (ITC)

Measurements were done on a VP-microcalorimeter (MicroCal) at 25°C.

Calcium binding to the BACHEMP-Cons peptide: The peptide, synthesized by Biomedal S.L. (Sevilla, Spain), was dissolved in polybuffer (5 mM Tris, 5 mM MES, 5 mM PIPES, pH 6.0) to a concentration of 50 µM and placed into the ITC sample cell. A freshly prepared CaCl_2_ solution (5 mM) was placed into the injector syringe. A typical experiment involved the injection of 3.2 µl aliquots of ligand into the peptide solution. The study of the pH optimum was performed in polybuffer adjusted to pH 5.0 to 9.0 with HCl. To determine the binding specificity, experiments were repeated in polybuffer, pH 6.0, containing 100 mM NaCl and ligands CaCl_2_, MgCl_2_, CuCl_2_, FeCl_2_, CrCl_2_ or MnCl_2_, each at a concentration of 5 mM.

Calcium binding to the N-terminal ANP-like domain of PepA: The purified protein dialyzed and concentrated as above reported at a final concentration of 11.2 µM in buffer 10 mM Tris-HCl, 50 mM NaCl, 10% glycerol, pH 7.5 was placed into the sample cell. A 1 mM CaCl_2_ solution was prepared using the dialysis buffer and placed into the injector syringe. A typical experiment involved the injection of 6.4 µl aliquots of ligand into the protein solution. Raw data were corrected for dilution effects and concentration was normalized prior to data analysis using the “one binding site” model of the MicroCal version of ORIGIN. During the fitting process *K*_A_, Δ*H* and n were left floating.

## Supporting Information

Figure S1 **Alignment of ANP-like domains of bacterial proteins containing two of these domains.** Numbering starts from the amino terminal end of the ANP-like domain according to PS50292. Bold text in boxes corresponds to five insertions found in *P. putida* sequences matching the consensus G-x-D-x(6)-D-D. Insertions A (2), B (3) and C (5) were firstly identified directly from the alignment whereas insertions 1 and 4 were identified after a second search with the consensus. In the alignment animal heme peroxidases of *Fulvimarina pelagi* HTCC2506 (Q0G341), Manganese-oxidizing bacterium (strain SI85-9A1) (Q1YMS2), *Methylobacterium chloromethanicum* CM4 / NCIMB 13688 (B7KW13), *M. extorquens* DSM 5838 / DM4 (A9W3A5), *M. extorquens* PA1 (C7CGY0), *Pseudomonas putida* F1 (A5W572), *P. putida* GB1 (B0KJL7), *P. putida* KT2440 (Q88JT6), *Rhodopseudomonas palustris* BisA53 (Q07SX1), *Rhodopseudomonas palustris* BisB5 (Q13AU2), *Roseobacter* sp. MED193 (A3XF15) and *Roseovarius* sp. TM1035 (A6E280). Letters -A and -B refer to N- and C-terminal ANP-like domains, respectively.
(DOCX)

Figure S2 **Alignment of bacterial ANP-like domains with G-x-D-G-x(5)-D-D insertions.** Numbering starts from the terminal end of the ANP-like domain according to PS50292. Sequences in the alignment are in Text S3. In the alignment ANP-like domains of *Arthrobacter* sp. FB24 (A0JUB7), *A. chlorophenolicus* A6 / ATCC 700700 (B8HDW8), *Cyanobium* sp. PCC 7001 (B5IMQ8), *Erythrobacter* sp. SD-21 (A5PER4), *Fulvimarina pelagi* HTCC2506 (Q0G341), *Leptothrix cholodnii* ATCC 51168 / LMG 8142 / SP-6 (B1Y442), Manganese-oxidizing bacterium (strain SI85-9A1) (Q1YMS2), *Mesorhizobium* sp. BNC1 (Q11K84), *Methylobacterium chloromethanicum* CM4 /NCIMB 13688 (B7KW13, B7L1G6, B7KRS2), *M. extorquens* DSM 5838 / DM4 (C7CGY0), *M. extorquens* PA1 (A9W3A5), *Nitrosomonas* sp. AL212 (C6MFN2), *Pseudomonas putida* F1 (A5W572), *P. putida* GB1 (B0KJL7), *P. putida* KT2440 (Q88JT6), *Rhodopseudomonas palustris* BisA53 (Q07SX1), *R. palustris* BisB5 (Q13AU2), *Roseovarius* sp. TM1035 (A6E280), *Roseobacter* sp. AzwK-3b (A6FKA5, A6FV45), and *Roseobacter* sp. MED193 (A3XF15). Letters –A and -B refer to as N- and C-terminal ANP-like domains, respectively. The domain of human myeloperoxidase (P05164) does not present the insertion and is included in this alignment as an out-group member. The alignment was performed with CLUSTAL 2.1. In bold and underlined residues contacting catalytic Ca in human myeloperoxidase.
(DOCX)

Figure S3 **Structure of the fragment that contains the G-x-D-G-x(5)-D-D consensus motif in 6 different three dimensional structures (shown in gold).** Bound calcium is shown in green. Motif sequence used in the initial search of PDB was G-x-D-G-x(5)-D-D (see the text in the main body of this article). Amino acids that correspond to the consensus defined are shown. pdb 1JJU and pdb 3G20 match PERCAL (G-x-D-G-x-x-[GN]-[TN]-x-D-D) and their motifs are highlighted in yellow. The figure was produced using the program WebLabViewer (http://www.marcsaric.de/index.php/WebLab_Viewer_Lite).
(DOCX)

Figure S4 **Preparation of recombinant PepA-Nter.** I, SDS-PAGE gel of the purified His-tagged Nter-PepA and II) spectrum of the heme-reconstituted PepA N-terminal domain. The purified N-terminal domain was incubated with hemin and exceeding heme was removed with a Sephadex G-25 column equilibrated with 10 mM Tris-HCl, 50 mM NaCl, 10% DMSO and 10% glycerol buffer (pH = 7.5). Displayed is the optical absorption spectrum of the heme reconstituted protein where (A) is the absorbance exhibited by the protein at 280 nm and (B) is the absorbance of bound heme at 410 nm.
(DOCX)

Table S1 **Detailed sequence analysis of the insertions interrupting the ANP-like domains of bacterial proteins.**
(XLSX)

Table S2 **Random occurrence and hits number of motif G-x-D-G-x(5)-D-D and some of its variants in the databank.**
(XLSX)

Text S1 **Consensus sequences of the insertions interrupting six** ***P. putida*** **ANP-like domains.**
(DOCX)

Text S2 **Sequences matching X-X-X-G-X-D-X(6)-[DE]-[DE]-X-X-X used to generate the consensus of** **figure 3** **.**
(DOC)

Text S3 **Sequences of the ANP-like domains of bacterial proteins used in this work according to Prosite profile PS50292.**
(DOCX)
