# Supplementary material for: Identification of a Novel Calcium Binding Motif Based on the Detection of Sequence Insertions in the Animal Peroxidase Domain of Bacterial Proteins
Source: PLoS One. 2012 Jul 13;7(7):e40698. doi: 10.1371/journal.pone.0040698 (PMC3396595; doi:10.1371/journal.pone.0040698)
Supplement: Figure S1 — Alignment of ANP-like domains of bacterial proteins containing two of these domains. Numbering starts from the amino terminal end of the ANP-like domain according to PS50292. Bold text in boxes corresponds to five insertions found in P. putida sequences matching the consensus G-x-D-x(6)-D-D. Insertions A (2), B (3) and C (5) were firstly identified directly from the alignment whereas insertions 1 and 4 were identified after a second search with the consensus. In the alignment animal heme peroxidases of Fulvimarina pelagi HTCC2506 (Q0G341), Manganese-oxidizing bacterium (strain SI85-9A1) (Q1YMS2), Methylobacterium chloromethanicum CM4 / NCIMB 13688 (B7KW13), M. extorquens DSM 5838 / DM4 (A9W3A5), M. extorquens PA1 (C7CGY0), Pseudomonas putida F1 (A5W572), P. putida GB1 (B0KJL7), P. putida KT2440 (Q88JT6), Rhodopseudomonas palustris BisA53 (Q07SX1), Rhodopseudomonas palustris BisB5 (Q13AU2), Roseobacter sp. MED193 (A3XF15) and Roseovarius sp. TM1035 (A6E280). Letters -A and -B refer to N- and C-terminal ANP-like domains, respectively. (DOCX) [file pone.0040698.s001.docx]

**Q88JT6-A** ---LPNSQVPFGLRTVDGSYNNLVTGQ----SEFGAADNSFLRLLEASYR------ANYV 47

**A5W572-A** ---LPNSQVPFGLRTVDGSYNNLVTGQ----SEFGAADNSFLRLLEASYR------ANYV 47

**B0KJL7-A** ---LPNSQVPFGLRTVDGSYNNLVAGQ----SEFGAADNSFLRLLDASYR------ANYV 47

**Q88JT6-B** ---IPNVRAAMGLRAVDGSNNNLMNLNGNNNTQYGAADNVFPRVTDPVFNPAEGAPAGFF 57

**A5W572-B** ---IPNVRAAMGLRAVDGSNNNLMNLNGNNNTQYGAADNVFPRVTDPVFNPAEGAPAGFF 57

**B0KJL7-B** ---IPNIRAPLGLRAVDGSNNNLMNLNGHNNTQFGAADNVFPRLTDPVFNPAEGAPAGFF 57

C7CGY0-A --AIADPHVPYGLRTVDGTYNNLVPGR----ETWGSSGQPMPQLFEPTYLNDADGDTMAL 54

A9W3A5-A --AIADPHVPYGLRTVDGTYNNLVPGR----ETWGSSGQPMPQLFEPTYLNDADGDTMAL 54

B7KW13-A --AIADPHVPYGLRTVDGTYNNLVPGR----ETWGSSGQPMPQLFEPTYLNDADGDTMAL 54

Q13AU2-A --AVADPKVPVGLRTVDGQDNNIVPGR----EQWGAADQSMPRLLTASYTTGAG--SIDL 52

Q07SX1-A --AIADPKVPAGLRTVNGEDNNIVPGR----EEWGAADQSMPRLLTSSYTTGAG--TLDL 52

Q0G341-A ------------------------------------------------------------

A6E280-A ------------------------------------------------------------

Q1YMS2-A NDLVPDPHVPWGLRTVDGTYNNLVDGR----EQWGAADTVMPRYLDGSFVTDTNSGAFFG 56

A3XF15-A ------------------------------------------------------------

Q1YMS2-B ------------------------------------------------------------

Q0G341-B ------------------------------------------------------------

A6E280-B ------------------------------------------------------------

C7CGY0-B ------------------------------------------------------------

A9W3A5-B ------------------------------------------------------------

B7KW13-B ------------------------------------------------------------

Q13AU2-B ------------------------------------------------------------

Q07SX1-B ------------------------------------------------------------

A3XF15-B ------------------------------------------------------------

**Q88JT6-A** GTGN---------------VVDSQPRTISNLIVDQTANNPAA-------------VEAN- 78

**A5W572-A** GTGN---------------VVDSQPRTISNLIVDQTANNPAA-------------VEAN- 78

**B0KJL7-A** GTGN---------------VVDSQPRTISNLIVDQTANNPAA-------------VEAN- 78

**Q88JT6-B** GPGSPAIPGSSYQQTS-GPVFDSQPRTISNLIVDQTSNNPAA-------------YATAY 103

**A5W572-B** GPGSPAIPGSSYQQTS-GPVFDSQPRTISNLIVDQTSNNPAA-------------YATAY 103

**B0KJL7-B** GPGSPAIPGSSYQQTS-GPVFDSQPRTISNLIVDQTSNNPAA-------------YATAY 103

C7CGY0-A GPGAP--VITNTNYGLPGSVADADPRIISNLVVDATLDNPAAIAAALRIAGSENVIADQR 112

A9W3A5-A GPGAP--VITNTNYGLPGSVADADPRIISNLVVDATLDNPAAIAAALRIAGSENVIADQR 112

B7KW13-A GPGAP--VITNTNYGLPGSVADADPRIISNLVVDATLDNPAAIAAALRIAGSENVIADQR 112

Q13AU2-A NGPAPGGVVTGGNYAAPGTIVDTAPRTVSNLIVDMSLNNPAAIIAALTFAGSEDVLADQS 112

Q07SX1-A NGPAPGGAVTGGNYAGPGTIVDTAPRTVSNLIVDMSLNNPAAIIAALTFAGSGDVLGDQG 112

Q0G341-A ------------------------------------------------------QLAAHV 6

A6E280-A ----------------------------TTVISSVTLPEPADGQWADLTLNSGPISAGLA 32

Q1YMS2-A --------VTNNNYAAPGSVVDTDPRIISNLIVDMSVDNPAA------------VLAFLN 96

A3XF15-A ------------------------------AAQDIETTKQSAFDT---------ATTNLD 21

Q1YMS2-B --------------------DDDTAGAGGVRELLGRNNNESH-----------PEYG-AA 28

Q0G341-B --------------------DDDTEGAAGVRELLGRNNNENN-----------PEFG-SA 28

A6E280-B --------------------DDDTEEATGVRTLSGEGNNEAN-----------PAYG-AA 28

C7CGY0-B -----------------PDNAGDDDVPTGYRELSGHGNNLDH-----------PTWG-SA 31

A9W3A5-B -----------------PDNAGDDDVPTGYRELSGHGNNLDH-----------PTWG-SA 31

B7KW13-B -----------------PDNAGDDDVPTGYRELSGHGNNLDH-----------PTWG-SA 31

Q13AU2-B --------------------GDDDDGATGVRDLSGHNNNVAN-----------PNWG-SA 28

Q07SX1-B -----------------PANGDDDGGPTGVRELSGHNNNQAN-----------PNWG-AA 31

A3XF15-B --------------------NEPADLIVGTRDLEGLTNNLLN-----------PEISGGA 29

Insertion 1

**Q88JT6-A** --------------------GGAAPVMSP**GIDGVFGTADD**KPVFFIPNVSPDAGLTAGFN 118

**A5W572-A** --------------------GGAAPVMSP**GIDGVFGTADD**KPVFFIPNVSPDAGLTAGFN 118

**B0KJL7-A** --------------------GGAAPVMSP**GIDGVFGTADD**KPVFFIPNVSPDVGLTAGFN 118

**Q88JT6-B** DPGADGVLNFGAAGNDDVLKDGVRIVASP**GMDGQFGTTDD**HDVYLFENTAADAGLSAPFN 163

**A5W572-B** DPGADGVLNFGAAGNDDVLKDGVRIVASP**GMDGQFGTTDD**HDVYLFENTAADAGLSAPFN 163

**B0KJL7-B** DPGADGVLNFGAPGNDDVLKDGVRIVASP**GMDGQFGTTDD**HDVYLFENTAADAGLSAPFN 163

C7CGY0-A AITAAHEALKAAQAANPAGDHAVLQSNLDALLEQTGVTVTNGSIDVLNVSPDEGLSKPFN 172

A9W3A5-A AITAAHEALKAAQAASPAGDHAVLQSNLDALLEQTGVTVTNGSIDVLNVSPDEGLSKPFN 172

B7KW13-A AITAAHEALKAAQAANPAGDHAVLQSNLDALLEQTGVTVTNGSIDVLNVSPDEGLSKPFN 172

Q13AU2-A EITAAFLALKAARDADPLGDHAALQLELDTILEQKGVTVTNGSIDVPNVAPDEGLSAPFN 172

Q07SX1-A EITAAFLALKAARDADPLGDHATLQQALDDVLEQKGVTVTNGSIEVPNVAPDEGLSAPFN 172

Q0G341-A GSELR-VEISHTGGGQALIDNVELSASSGNRIEITDEDLAT----LPNIAPDDGISAPFN 61

A6E280-A GQTLR-VEIQQTGGSQVLVDNVALSTSNGNEIEIDNIDLAT----IPNIAPDDGISAPFN 87

Q1YMS2-A NELAVETFKELHGGLEPVAPGTVVNS--ATQLAVTDADLAL----IPNIAPDEGISAPFN 150

A3XF15-A ALNAPGAADAALAAAQAIAT--EAQTTLDTLLSTHAITMDGNNVMLPDVTPDEGLSAPYN 79

Q1YMS2-B DEVFIRLTEARYGEYDGTTNNRAINPIFAGLDARTISNVLG-----HQEADLSPAASGAN 83

Q0G341-B DEVFIRLTEARYGEYDATINNRAVNPIFAGLDPREISNILG-----VQEADLAPAKSGAN 83

A6E280-B GEPFIRLTEARYGDPDENGN-RQINPIFDGLDPRAISNILG-----PHDDTTAPNAMNAS 82

C7CGY0-B DQAFIRLTQARYGETDANGN-RAINPIFDGLDARTISNILG-----TQEAGLPKAGNDAN 85

A9W3A5-B DQAFIRLTQARYGEADANGN-RAINPIFDGLDARTISNILG-----TQEAGLPKAGNDAN 85

B7KW13-B DQAFIRLTQARYGEADANGN-RAINPIFDGLDARTISNILG-----TQEAGLPKAGNDAN 85

Q13AU2-B DQPFIRITNPHYGEADANGN-LAINPVFDGLDPRTISNVLG-----SQEAGLPSAGNDAN 82

Q07SX1-B DQPFIRITNAHYGDEDANGN-RAINPVFDGLDPRTISNILG-----TQEANLPHAGNDAN 85

A3XF15-B TLPFSRVTEARYAGIGEDGA-GIVNPVFDDLDARAISNALG-----AQDADAAKAAS-AN 82

. .

**Q88JT6-A** AWMTFFGQFFDHGLDLVSKSSTDIVFIPLRPDDPLFVAGS------------PTNFMVLS 166

**A5W572-A** AWMTFFGQFFDHGLDLVSKSSTDIVFIPLRPDDPLFVAGS------------PTNFMVLS 166

**B0KJL7-A** AWMTFFGQFFDHGLDLVTKSSTDIVFIPLRPDDPLYNASS------------PTNFMVLS 166

**Q88JT6-B** AWMTFFGQFFDHGLDLVTKGGSGTIYIPLQPDDPLYVEGG------------FTNFMVVT 211

**A5W572-B** AWMTFFGQFFDHGLDLVTKGGSGTIYIPLQPDDPLYVEGG------------FTNFMVVT 211

**B0KJL7-B** AWMTFFGQFFDHGLDLVTKGGSGTIYIPLQPDDPLYVEGG------------FTNFMVVT 211

C7CGY0-A AWMTFFGQFFDHGLDLISKGGNGTVYVPLAADDPL-VLGQDGLAGTADDLAPHLRFMTLT 231

A9W3A5-A AWMTFFGQFFDHGLDLISKGGNGTVYVPLAADDPL-VLGQDGLAGTADDLAPHLRFMTLT 231

B7KW13-A AWMTFFGQFFDHGLDLISKGGNGTVYVPLAADDPL-VLGQDGLAGTADDLAPHLRFMTLT 231

Q13AU2-A AWMTFFGQFFDHGLDLISKGGAGTVYVPLAADDPLRTHGPDGIAGTGDEVPGQMAFMALT 232

Q07SX1-A AWMTFFGQFFDHGLDLISKGGAGTIYVPLAADDPLRTHGPDGVAGTGDEVSEQMAFMALT 232

Q0G341-A AWMTFFGQFFDHGLDLITKGGNGTVFIPLQADDPL-VVSG--------QVPPHMQFMVLT 112

A6E280-A AWMTFFGQFFDHGLDLITKGDNGTVFIPLQADDPL-VLGADGIAGTADDLPNHLRFMALT 146

Q1YMS2-A GWTTFFGQFFDHGLDLITKGTNGTVYIPLQPDDPLYVPGG------------FTNFMVLT 198

A3XF15-A SWMTLFGQFFDHGLDLVGKGGSGTVYIPLQPDDPLYDATS------------PTNFMVLT 127

Q1YMS2-B TFFMAFGQYFDHGLDFLPKNSANGVLAIGGPGTSRAPGVD--------------NPADLT 129

Q0G341-B TFFMAFGQYFDHGLDFLPKDSLNGVIEIGGPGSARAPGVD--------------NPADLT 129

A6E280-B ALFMAFGQYFDHGLDFIAKNPAFGTIEIGGPGAERSPTSD--------------NPADLT 128

C7CGY0-B IFFMAMGQYIDHGLDFLPKG-GNGSIVIGAPGG-GAPGSN--------------NPADLT 129

A9W3A5-B IFFMAMGQYIDHGLDFLPKG-GNGSIVIGAPGG-GAPGSN--------------NPADLT 129

B7KW13-B IFFMAMGQYIDHGLDFLPKG-GNGSIVIGAPGG-GAPGSN--------------NPADLT 129

Q13AU2-B IFFMAMGQYIDHGLDFLGKG-GNGTIQIGALGG-GAPGSG--------------NPADLT 126

Q07SX1-B IFFMAMGQYIDHGLDFLGKG-GNGSIQIGAAGG-GAPGSD--------------NPADLT 129

A3XF15-B MFMMSFGQYFDHGLTFIPKG-GHDPITIGGADM-GRPSGD--------------NPADLT 126

:**::**** :: *. . ::

Insertion 2(=A)

**Q88JT6-A** RAVRTA**GA---DGVVGTADDS**--QPNTTSPFVDQSQTYSSHPSHQVFLREYTVNAAGDPV 221

**A5W572-A** RAVRTA**GA---DGVVGTADDS**--QPNTTSPFVDQSQTYSSHPSHQVFLREYTVNAAGEPV 221

**B0KJL7-A** RAVRTA**GA---DGVVGTADDG**--QPNTTSPFVDQSQTYSSHPSHQVFLREYMLDAAGDPV 221

**Q88JT6-B** RATNLP**GP---DGILGNADDI**REHTNTTTPFVDQNQTYSSHPSHQVFLRAYVMTDDG-PV 267

**A5W572-B** RATNLP**GP---DGILGNADDI**REHTNTTTPFVDQNQTYSSHPSHQVFLRAYVMTDDG-PV 267

**B0KJL7-B** RATNLP**GP---DGILGNADDI**HEHTNTTTPFVDQNQTYSSHPSHQVFLRAYVMTDDG-PV 267

C7CGY0-A RAAQVE---------------GSQRNVTTPFVDQNQTYTSNASHQVFLREYALVD-GRPV 275

A9W3A5-A RAAQVE---------------GSQRNVTTPFVDQNQTYTSNASHQVFLREYALVD-GRPV 275

B7KW13-A RAAQVE---------------GSQRNVTTPFVDQNQTYTSNASHQVFLREYALVD-GRPV 275

Q13AU2-A RATPAAD--------------GSQVNTTTPFVDQNQTYTSHASHQVFLREYKMVG-GVPM 277

Q07SX1-A RATPASD--------------GSQTNTTTPFVDQNQTYTSHASHQVFLREYSMAS-GVPM 277

Q0G341-A RSTPTEGP---------DGSMTEGKNVTTPFVDQNQTYTSHASHQVFVREYEMVD-GRPI 162

A6E280-A RSTPVDGPGADGVLGTADDTQHEGQNTTTPFVDQNQTYTSHASHQVFLREYAFDTNGNPV 206

Q1YMS2-A RAAKAEHLPGEDGVLGTADDIVSHTNTTTPFVDQNQTYTSHASHQVFLREYKFNADGEPV 258

A3XF15-A RATNQPGP---DGILGTADDVREHFNKTTPWVDQNQTYTSHPSHQVFLREYDLDANGSPV 184

Q1YMS2-B RGEVYTIDEN---------GVPQHLNKASPFVDQNQAYGSNALVGQFLRE----SDGDQG 176

Q0G341-B RGKVHVIDEN---------GIPQHLNKASPFVDQNQAYGSNELVGQFLRE----SDGAQG 176

A6E280-B RAEVAGYDED---------GVPQHTNMTSPFVDQNQAYGSHELVGQFLRE----SDGAHG 175

C7CGY0-B RGTVMAVDAN---------GVPQHKNQTSPYIDQNQAYGSNALVGQFLRE----SDGAQG 176

A9W3A5-B RGTVMAVDAN---------GVPQHKNQTSPYIDQNQAYGSNALVGQFLRE----SDGAQG 176

B7KW13-B RGTVMAVDAN---------GVPQHKNQTSPYIDQNQAYGSNALVGQFLRE----SDGAQG 176

Q13AU2-B RGSVASYDAN---------GVPQHINRTSPYVDQNQAYGSNDLVGQFLRA----GDDNGG 173

Q07SX1-B RGSVAGYEN----------GVPQHVNRTSPYVDQNQAYGSNDLVGQFLRE----GDGNGG 175

A3XF15-B RATATINPET---------GEIEHTNITSPVVDQNQVYGSSNLVGQLLRE----SGSNGG 173

*. * ::* :**.*.* * ::* .

Insertion 3(=B)

**Q88JT6-A** ATGRLITNRDL**GADGKFGTADD**GNGESGGMATWAVVKAQARDLLGINLTDADVHSVPLLA 281

**A5W572-A** ATGRLITNRDL**GADGKFGTADD**GNGESGGMATWAVVKAQARDLLGINLTDADVHSVPLLA 281

**B0KJL7-A** ATGRLITNRDL**GADGKFGTADD**GNSENGGMATWAVVKAQARDLLGINLTDADVHSVPLLA 281

**Q88JT6-B** ATGRLITNRDL**GADGRFGTADD**—-TEIGGMATWKVVKAQARDLLGINLTDADVDNVPLLA 325

**A5W572-B** ATGRLITNRDL**GADGRFGTADD**—-TEIGGMATWKVVKAQARDLLGINLTDADVDNVPLLA 325

**B0KJL7-B** ATGRLITNRDL**GADGKFGTADD**—-TEIGGMATWKVVKAQARDLLGINLTDADVDNVPLLA 325

C7CGY0-A ATGRLLG----GAD-------------GGLATWADVKFQARTILGIELTDADVSAVPQLL 318

A9W3A5-A ATGRLLG----GAD-------------GGLATWADVKFQARTILGIELTDADVSAVPQLL 318

B7KW13-A ATGRLLG----GAD-------------GGLATWADVKFQARTILGIELTDADVSAVPQLL 318

Q13AU2-A ATGKLLG----GAE-------------GGLATWADVKAQAQNMLGIVLSDLDVLNVPLLR 320

Q07SX1-A ATGKLLG----GAD-------------GGLATWADVKAQARDALGIELSDLDVFNVPLLR 320

Q0G341-A STGHLLD----GAN-------------GGLATWAEVKVQAAEKLGIALADGDAVSIPLIL 205

A6E280-A STGKLLD----GAN-------------GGIPTWAEVKAQARDLLGIELTDGDVLNIPLLR 249

Q1YMS2-A STGRLLD----GLE-------------GGLATWGQIKAEAAAKLGIALDDQDALNIPLLR 301

A3XF15-A SNGYLLH----GQS-------------GGMSTWGDVKAQAAAKLGIQLNDSDVLDGPLLA 227

Q1YMS2-B VGMRLLS---------------------GATDPSTPDFNLLPTLRELIAHHWENDTIFVD 215

Q0G341-B FGMRLLA---------------------GADDPSNPEFRLLPTLRELIEHHWEANTIFRD 215

A6E280-B FGMRLLG---------------------GEADPSDPAFTLLPTLRDLILHHWEAETYFED 214

C7CGY0-B VGMRLLA---------------------GAPDPSNPAFNLLPTLRELVNHHWQADTIFAG 215

A9W3A5-B VGMRLLA---------------------GAPDPSNPAFNLLPTLRELVNHHWQADTIFAG 215

B7KW13-B VGMRLLA---------------------GAPDPSNPAFNLLPTLRELVNHHWQADTIFAG 215

Q13AU2-B LGAHLFA---------------------GAPDPSNPEFALLPTLRELITEHWQNNTVFHS 212

Q07SX1-B VGAHLFA---------------------GGPDPSNPQFSLLPTLRELIEEHWSNNTVFHS 214

A3XF15-B FGAHVLM---------------------GQEDPSASGFQLMATLRELLDHHTQAGTVFTD 212

:: * * : .

Insertion 4

**Q88JT6-A** TDAYGNFLRGPNGMPQVVMRVNN**GADGI-AGTADD**VTQLVEGDRNAPISLANA------- 333

**A5W572-A** TDAYGNFLRGPNGMPQVVVRVNN**GADGI-AGTADD**VTQLVEGDRNAPISLANA------- 333

**B0KJL7-A** TDAYGNFLRGPNGMPQVVMRVNN**GADGI-AGTADD**VTTLVEGNRAAPISLANA------- 333

**Q88JT6-B** TDAYGNFIKGPNGYPMVVM---K**GLDGI-AGTADD**--QEVEGNPLAPLDLTNA------- 372

**A5W572-B** TDAYGNFIKGPNGYPMVVM---K**GLDGI-AGTADD**--QEVEGNPLAPLDLTNA------- 372

**B0KJL7-B** TDAYGNFIKGPNGYPMVVM---K**GVDGI-AGTADD**--QQVEGNPLAPISLTNA------- 372

C7CGY0-A VDAYGEFVRSANGLPQVMVG--VGPGGQ-AVYASG--SLAEPLKLSAIQLPVGTVLVGPN 373

A9W3A5-A VDAYGEFVRSVNGLPQVMVG--VGPGGQ-AVYASG--SLAEPLKLSAIQLPVGTVLVGPN 373

B7KW13-A VDAYGEFVRSANGLPQVMVG--VGPGGQ-AVYASG--SLAEPLKLSAIQLPVGTVLVGPN 373

Q13AU2-A TDAYGEFIRDANGFAQVIIG--LGDDGI-PNTADD--LVASGTNLAPLNLATLNNGAGP- 374

Q07SX1-A TDPYGEFIRDANGFPQVIVG--IGADGI-PNTADD--IVASGTNLAPFDLASLNGGLGP- 374

Q0G341-A TDAYGEFVRGPNGFPQVVLG--VGPDGI-PNTADD--IVVEGDPENPINTFTVQSEDGS- 259

A6E280-A TDDYGEFIRGPNGLPQIVVG--IGADGI-PNTADD--DVVEGNLAAPVNTFTA------- 297

Q1YMS2-A TDPYGEFIRGDNGLPMIVTG--LGPDGI-PNTADD--IVVQGNLTTPVNTMAIG------ 350

A3XF15-A TDAYGNFIPGANGLPQLVVA--NPAYVEGGTEPLN--ILIEGDLANPVDASQA------- 276

Q1YMS2-B PSLPGGSVSFRDYFTDYPISDNATGSIFDEATGAYDPDVVASMVSNFMGGGYP------- 268

Q0G341-B PSLPNGAISFREYFTDFPISEGVTGNLFDEATGAYDPDVVNHLVSDFMGGGYP------- 268

A6E280-B PGLPGGAARLQDVFPD----------LVDEITGEIDPDMVQALASDFLGSGQP------- 257

C7CGY0-B ---PDGPISFRTYYTNFALSEGVTGTLFNTETGAFDPQVLTKLVGNFMGSGHP------- 265

A9W3A5-B ---PDGPISFRTYYTNFALSEGVTGTLFNTETGAFDPQVLTKLVGNFMGSGHP------- 265

B7KW13-B ---PDGPISFRTYYTNFALSEGVTGTLFNTETGAFDPQVLTKLVGNFMGSGHP------- 265

Q13AU2-B SSLQGGSVAFRDYFAGLVG-----------QNGVINQALLPSMISNFMGTNHA------- 254

Q07SX1-B ESLPDGAVAFRDYFPGLV------------QGGVINTAMLPGMISNFMGTSHA------- 255

A3XF15-B TD--KGDVTLEGYYPDLFN-----------EDGTYNAATIKDLSDDFMGEGWP------- 252

. .

**Q88JT6-A** --------------VSTGHGFLDDIAHNAAP--------VVVGGALQADADTLVGNAQPV 371

**A5W572-A** --------------VSTGHGFLDDIAHNAAP--------VVVGGVLQADADTLVGNAQPV 371

**B0KJL7-A** --------------VSTGHGFLDDIAHNAEP--------VKVGGVLQADADSAVGNVQPV 371

**Q88JT6-B** --------------VRTGHQFLADIAHNAVP--------VFSGGVLAPDADNVAGNAVPV 410

**A5W572-B** --------------VRTGHQFLADIAHNAVP--------VFSGGVLAPDADNVAGNAVPV 410

**B0KJL7-B** --------------VRTGHQFLADIAHNAVP--------VFSGGVLAPDADNAVGNAVPV 410

C7CGY0-A GAQNVIEAGETVAAARTFNAFLDDIAHNAVP--------VAVNGVLRPDADALTGNAVQM 425

A9W3A5-A GAQNVIEAGETVAAARTFNAFLDDIAHNAVP--------VAVNGVLRPDADALTGNAVQM 425

B7KW13-A GAQNVIEAGETVAAARTFNAFLDDIAHNAVP--------VAVNGVLRPDADALTGNAVQM 425

Q13AU2-A --------------VRTSHAFLDDIAHNAAP--------VVVDGVLTPDTDSATGNDVAI 412

Q07SX1-A --------------VRTSHAFLDDIAHNAAP--------VVVGGVLAPDADSATGNDVAI 412

Q0G341-A ----------LTGAVRIGHAFLDDIAHAANP--------VDSQTGLLK------------ 289

A6E280-A ------------GAIRIGHAFLDDIAHAANP--------FDSQSGMLKTADDDT--AVGL 335

Q1YMS2-A -------------AIRIGHAFLDDIAHNAAP--------VINGGVLQPDADVLTGNTVAS 389

A3XF15-A --------------VRNGHAFLEDIAHNAVPGTYVVDRFTGETATKQADADTDTGNAIIP 322

Q1YMS2-B ------------------------LLLDTNP----------------------------- 275

Q0G341-B ------------------------LLLDTNP----------------------------- 275

A6E280-B ------------------------LLLDANP----------------------------- 264

C7CGY0-B ------------------------LLLDTNP----------------------------- 272

A9W3A5-B ------------------------LLLDTNP----------------------------- 272

B7KW13-B ------------------------LLLDTNP----------------------------- 272

Q13AU2-B ------------------------LLLDANP----------------------------- 261

Q07SX1-B ------------------------LVLDANP----------------------------- 262

A3XF15-B ------------------------LLIDTNP----------------------------- 259

: : *

**Q88JT6-A** G-QGGNNLTYDNELLDAHYIAGDGRVNENIGLTAVHHVFHSEHNRLVQQTKDTLLAAGDL 430

**A5W572-A** G-QGGNNLTYDNELLDAHYIAGDGRVNENIGLTAVHHVFHSEHNRLVQQTKDTLLAAGDL 430

**B0KJL7-A** G-PGGNNLTYDNELLDAHYIAGDGRVNENIGLTAVHHVFHSEHNRLVQQTKDTLLAAGDL 430

**Q88JT6-B** NPQTGANLAYDNELLDAHYIAGDGRVNENIGLTAVHAIFHSEHNRLVAQTMDTVLDSGDL 470

**A5W572-B** NPQTGANLAYDNELLDAHYIAGDGRVNENIGLTAVHAIFHSEHNRLVAQTMDTVLDSGDL 470

**B0KJL7-B** NPQTGANLAYDNELLDAHYIAGDGRVNENIGLTAVHAIFHAEHNRLVAQTMDTVLDSHDL 470

C7CGY0-A NPQTGRNLEYDNELLDRHFVTGDGRGNENIGLTAVHHIFHSEHNRQIDAHKLTILQSGDL 485

A9W3A5-A NPQTGRNLEYDNELLDRHFVTGDGRGNENIGLTAVHHIFHSEHNRQIDAHKLTILQSGDL 485

B7KW13-A NPQTGRNLEYDNELLDRHFVTGDGRGNENIGLTAVHHIFHSEHNRQIDAHKLTILQSGDL 485

Q13AU2-A NPLTGQRLEYDDELLDRHYITGDGRGNENIGLTAVHHIFHSEHNRQVDSQKLTILNSGDI 472

Q07SX1-A NPLTGQRLEYDNELLDRHYITGDGRGNENIGLTAVHHIFHSEHNRQVDSQKLTILRSGDT 472

Q0G341-A -----AEGTYDSELLGRHFITGDGRGNENIALTSVHHVFHSEHNRQVEDQKKTILETGDL 344

A6E280-A SDSVSTAGTYDNELLDRHFVTGDGRGNENIGLTAVHHVFHSEHNRQVVAQKKTILESGDI 395

Q1YMS2-A --QQGQNTEYDNELLDRHYITGDGRGNENIALTAVHHVFHSEHNRLVDATRMEVLKSGDL 447

A3XF15-A N-QFGQNETYDNELLDRHFIAGDGRGNENFGLTAVHHVFHSEHNRQTTEMKQTILDSGEL 381

Q1YMS2-B -----------YINLLDHYVAGDGRANENFALTSMHTIWARNHNFHVEMLLEAGFEG--- 321

Q0G341-B -----------FINLLDHYIAGDGRANENFALTSMHTVWARNHNFHVETLMEAGFEG--- 321

A6E280-B -----------FIDLLDHRMAGDGRANENFALTSVHTVWARNHNFHVENMLAQGFEG--- 310

C7CGY0-B -----------FISVLDHFVAGDGRANENFALTSIHTVWARNHNYHVEKLLESGFEG--- 318

A9W3A5-B -----------FISVLDHFVAGDGRANENFALTSIHTVWARNHNYHVEKLLESGFEG--- 318

B7KW13-B -----------FISVLDHFVAGDGRANENFALTSIHTVWARNHNYHVEKLLESGFEG--- 318

Q13AU2-B -----------FINVLDHYVSGDGRTNENFALTSIHTIWARNHNHHVEGLAAAGFQG--- 307

Q07SX1-B -----------FINVLDHYVAGDGRANENFALTSIHTIWARNHNHHVEGLEAAGFQG--- 308

A3XF15-B -----------FMNLLDHFVGGDGRANENVGLTSMHTVWARNHNYHVDQLLASGYDAD-- 306

: * : **** ***..**::* :: :**

**Q88JT6-A** AFLNEWLIDDVTAIPTT---------PADIAALVWDGERLFQAAKFGTEMQYQHLVFEEF 481

**A5W572-A** AFLNEWLIDDVTAIPTA---------PADIAALVWDGERLFQAAKFGTEMQYQHLVFEEF 481

**B0KJL7-A** AFLNEWLIDDVIAIPTT---------PAGIAALVWDGERLFQAAKFGTEMQYQHLVFEEF 481

**Q88JT6-B** AFLNEWLLNPVSALPVT---------PAEIGALVWNGERLFQAAKFGTEMQYQHLVFEEF 521

**A5W572-B** AFLNEWLLNPVSALPVT---------PAEIDALVWNGERLFQAAKFGTEMQYQHLVFEEF 521

**B0KJL7-B** AFLNEWLLNPVTALPVT---------PAEIDALVWNGERLFQAAKFGTEMQYQHLVFEEF 521

C7CGY0-A AFINDWLATDIAALPGNFAQMTALGQLAYANTLSWDGERLFQAARFATEMQYQHLVFEEF 545

A9W3A5-A AFINDWLATDIAALPGNFAQMTALGQLAYANTLSWDGERLFQAARFATEMQYQHLVFEEF 545

B7KW13-A AFINDWLATDIAALPGNFAQMTPLGQLAYANTLSWDGERLFQAARFATEMQYQHLVFEEF 545

Q13AU2-A AFINEWLATDIGALDPGFGTMTALQQLDYANSLNWDGERLFQGARFATEMQYQHLVFEEF 532

Q07SX1-A AFINEWLATDIGGLPSGFASLSGLDQLAYANSLNWDGERLFQAARFATEMQYQHLVFEEF 532

Q0G341-A EMLNEWLAVDVSEVPT---------DPAVIATLSWDGERLFQAARFATEMQYQHLVFEEF 395

A6E280-A DFINEWLLVDLAAGDP---------IPTDPTALTWDGERLFQAGRFATEMQYQHLVFEEF 446

Q1YMS2-A AFINEWLATDIATLEG--IPADGLPLLNFANTLDWDGERVFQAARFGTEMQYQHLVFEEF 505

A3XF15-A AFINEWLATPINEDELS---------TAAIDTLTWDGGRLFQAAKFTTEMQYQHLAFEEF 432

Q1YMS2-B -----------------------------------TEEEVFQAAKMINEAEYQRVVFTEF 346

Q0G341-B -----------------------------------TSEEFFQAAKMLNEAEYQRVVFDEF 346

A6E280-B -----------------------------------SDEEIFQAAKMLNESDYQRVVFQEF 335

C7CGY0-B -----------------------------------TPEQVFQAAKMVNEAEYQRVVFDEY 343

A9W3A5-B -----------------------------------TPEQVFQAAKMVNEAEYQRVVFDEY 343

B7KW13-B -----------------------------------TPEQVFQAAKMVNEAEYQRVVFDEY 343

Q13AU2-B -----------------------------------TAEELFQAAKMINEAEYQRVVFDEY 332

Q07SX1-B -----------------------------------TAEELFQAAKMINEAEYQRVVFDEY 333

A3XF15-B -----------------------------------TPEELFQAARILNIGEYQQVVFNDF 331

..**..:: . :**::.* ::

**Q88JT6-A** ARTIQPQIDEFLAP------NGYDTSINPAILAEFAHVVYRFGHSMLTETVDRYDPAFNP 535

**A5W572-A** ARTIQPQIDEFLAP------NGYDTSINPAILAEFAHVVYRFGHSMLTETVDRYDPAFNP 535

**B0KJL7-A** ARTIQPQIDEFLAP------NGYDTSINPAILAEFAHVVYRFGHSMLTETVDRFDPSFNP 535

**Q88JT6-B** ARTVQPRVDLFFAPT-----QVYDVDLDASIVAEFAHTVYRFGHSMLTETVDRFDIDFNV 576

**A5W572-B** ARTVQPRVDLFFAPT-----QVYDVDLDASIVAEFAHTVYRFGHSMLTETVDRFDIDFNV 576

**B0KJL7-B** ARTVQPRVDLFFAPT-----QVYDVDLDASIVAEFAHTVYRFGHSMLTETVDRFDIDFNV 576

C7CGY0-A ARKIQPLVDPFVFN--------PVTEIDPSIFAEFANTVYRFGHSMLTENMPRLGP---- 593

A9W3A5-A ARKIQPLVDPFVFN--------PVTEIDPSIFAEFANTVYRFGHSMLTENMPRLGP---- 593

B7KW13-A ARKIQPLVDPFVFN--------PVTEIDPSIFAEFANTVYRFGHSMLTENMPRLGP---- 593

Q13AU2-A ARKIQPAIDPFVFN--------SVTDINPAIFSEFANTVYRFGHSMLTEAMPRLDA---- 580

Q07SX1-A ARKIQPAIDPFVFN--------SVTDINPAIFSEFANTVYRFGHSMLTEGMPRLDG---- 580

Q0G341-A GRKINPNIDPFVFN--------AVTDINPAIFAEFANVVYRFGHSMLTDNMPRVFVDE-- 445

A6E280-A GRKIHPNIDPFVFN--------AVTDINPSIFAEFANVVYRFGHSMLTENMPRVLVNE-- 496

Q1YMS2-A ARKIQPAIDPFVFN--------SSTDIDPSIFSEFANVVYRFGHSMLTETVARTNIH--- 554

A3XF15-A GRTVQPQIAAFMVN--------ASAEVDASIMAEFAHVVYRFGHSMLTENVQTMDP---- 480

Q1YMS2-B ADMLIGGIRGEG----DHGFNDYNPNADARISHEFASAVYRVGHSLVGQTMTVIGPDGQ- 401

Q0G341-B ADFLIGGIRGSG----SHGHDEYNPDVDARISHEFAAAVYRVGHSLVGQTMTVIGPDGQ- 401

A6E280-B ADKLLGGLRNADGDREDHGWDGYNPDVDARISHEFAAAAYRFGHSLVGENLQVQGPNGE- 394

C7CGY0-B LETLIGGLRSDG----THGFEAYDPSVDVAISHEFAAAVFRFGHSLIGQTLNVKGADGE- 398

A9W3A5-B LETLIGGLRSDG----THGFEAYDPSVDVAISHEFAAAVFRFGHSLIGQTLNVKGADGE- 398

B7KW13-B LETLIGGLRSDG----THGFEAYDPNVDVAISHEFAAAVFRFGHSLIGQTLNVKGADGE- 398

Q13AU2-B LETLLGGLRSQG----THGFEEYDPNANAGISHEFAGAVFRFGHSLIGQTMTVLDANGN- 387

Q07SX1-B LETLLGGLRSQG----THGFEEYDPGADAGISHEFAAAVFRFGHSLIGQTMTVLDADGN- 388

A3XF15-B ADSLLGGLQGSG----THGHDKYDPTTDARISHEFAAAAYRFGHSQIGQSMTLKDVDADG 387

: : : * *** ..:*.*** : : :

**Q88JT6-A** VSA-DPANPDQQLGLIAAFLNPLAFA----------------GSGATADEAAGAIIRGVT 578

**A5W572-A** VSA-DPANPDQQLGLIAAFLNPLAFA----------------GSGATADEAAGAIIRGVT 578

**B0KJL7-A** VSG-DPANPDQQLGLIAAFLNPLAFA----------------GSGATADEAAGAIIRGVT 578

**Q88JT6-B** IQDPASANPDQQLGLIAAFLNPLAYA----------------ASGVTPEDATSAIVRGVT 620

**A5W572-B** IQDPASANPDQQLGLIAAFLNPLAYA----------------ASGVTPEDATSAIVRGVT 620

**B0KJL7-B** IKDPASANPDQQLGLIAAFLNPLAYA----------------ASGVTPEDATSAIVRGVT 620

C7CGY0-A ----DGQALDAGLGLIDAFLNPLAFD---------------NDGGLSHDESAAAIMRGMT 634

A9W3A5-A ----DGQALDADLGLIDAFLNPLAFD---------------NDGGLSHDESAAAIMRGMT 634

B7KW13-A ----DGQALDADLGLIDAFLNPLAFD---------------NDGGLSHDESAAAIMRGMT 634

Q13AU2-A ----NGNPMDSELGLVESFLNPVLFD---------------NDGAISHDAGAAAIVRGMT 621

Q07SX1-A ----AGNSMDSDLGLVEAFLNPVLFD---------------NDGAISHDAGAAAIVRGMT 621

Q0G341-A ---TTGEVSTDDMGLIQAFLNPDVFKRDG------------NDNEISADEAAAAIVRGMT 490

A6E280-A ---LTGEVTTDNMGLIAAFLNPVAYD---------------NDGAMSADAAAAAVILGMT 538

Q1YMS2-A ------DGSADNIGLIQAFLNPVEFT---------------KNSTVSADEATASIVLGMT 593

A3XF15-A ------NGVNTSTGLIEAFLNPVAFD---------------LDQTLTSDQAAGAVARGMS 519

Q1YMS2-B ---------PRQVELFDAFLNPTSELGAFKPGLP----DGYVPQPGYAQLGAGAILAGVA 448

Q0G341-B ---------PREVALFDAFLNPTNEAGAFTGPLP----PGYVPQPGYAQLGVGAILSGTA 448

A6E280-B ---------LIQVPLYDAFLNPSNDPSVFNGPLP----QGYVPAPGYAQYGVAAIIGGTA 441

C7CGY0-B ---------TVPVSLFDAFLNPSNDPSVFTAPLP----PGYVPQPGYAQYGVGGIIGGTI 445

A9W3A5-B ---------TVPVSLFDAFLNPSNDPSVFTAPLP----PGYVPQPGYAQYGVGGIIGGTI 445

B7KW13-B ---------TVPVSLFDAFLNPSNDPSVFTAPLP----PGYVPQPGYAQYGVGGIIGGTI 445

Q13AU2-B ---------PTQVNLFDAFLNPSNDPSAFPNPLP----PGYTPQPGYAQHGVNAIIGGTV 434

Q07SX1-B ---------PTQVNLFDAFLNPSNDPSVFPSPLP----PGYTPQPGYAQHGVNAIIGGTV 435

A3XF15-B ------NPFTVEVPLFDIFLNPTNDPDAFTADFGTLEQYGYKPQSGYAQYGVDNILGGLV 441

* **** : .. : *

**Q88JT6-A** RQVGNEIDEFVTEALRNNLLGLPLDLPALNIARGRDTGIPSLNEARREFYAATGDSQLKP 638

**A5W572-A** RQVGNEIDEFVTEALRNNLLGLPLDLPALNIARGRDTGIPSLNEARREFYAATGDSQLKP 638

**B0KJL7-A** RQLGNEIDEFVTEALRNNLLGLPLDLPALNIARGRDTGIPSLNEARREFYGATGDSQLKA 638

**Q88JT6-B** RQAGNEIDEFVTEALRNNLLGLPLDLPAINIARGRDVGIPSLNAVRREIYGQTGDTQLKP 680

**A5W572-B** RQAGNEIDEFVTEALRNNLLGLPLDLPAINIARGRDVGIPSLNAVRREIYGQTGDTQLKP 680

**B0KJL7-B** RQGGNEIDEFVTEALRNNLLGLPLDLPAINIARGRDVGIPSLNAVRRDVYGQTGDTQLKP 680

C7CGY0-A IERGSEIDEFVVGALRNNLLGLPLDLAAINIARGRDTGTPTLNEARAQLYAATGSTFLTP 694

A9W3A5-A IERGSEIDEFVVGALRNNLLGLPLDLAAINIARGRDTGTPTLNEARAQLYAATGSTFLTP 694

B7KW13-A IERGSEIDEFVVGALRNNLLGLPLDLAAINIARGRDTGTPTLNEARAQLYAATGSTFLTP 694

Q13AU2-A IERGNEIDEFVVDALRNNLLGLPLDLAAINIARGRDTGMPSLNETRTQLYAASGSTFLKP 681

Q07SX1-A IERGNEIDEFVVDALRNNLLGLPLDLAAINIARGRDTGMPSLNDARTQLYAASGSTFLKP 681

Q0G341-A TERGSAIDEYVVSSLRSNLLGLPLDLPALNIARGRETGMPTFNDARAELYGQTNSVWLKP 550

A6E280-A TEQGSQIDEFIVPALRSNLLGLPLDLAAINIARGRDTGIPSFNDARAELFQQTNSVWLKP 598

Q1YMS2-A SEHGNAIDEFITSALRNNLLGLPLDLAAINIARGRDTGMPTLNETREQLYQATGSSFLKP 653

A3XF15-A RETGANIDEFITSALRDNLVGLPLDLAALNITRGRDTGVPSLNAAREQFYAATGSEFLKP 579

Q1YMS2-B TQSAEEVDFNIVDAIRNDLVRINADLFAFNVARGWDVGLGTLNQVRADLKASGDPYIQEA 508

Q0G341-B IQPAEEVDFNIVDAIR-------------------TTSFG-------------------- 469

A6E280-B VQAAEEVDLKIVEAIRSDLVRINADLFSFNVARGWDVGLGTMNQVRTQLAASTDPYVSQA 501

C7CGY0-B EQAAEDVDFNIVDAVRNDLVRIRADLFAFNVARGWDVGLGTLNQVRADLAASTNPYIRDA 505

A9W3A5-B EQAAEDVDFNIVDAVRNDLVRIRADLFAFNVARGWDVGLGTLNQVRADLAASTNPYIRDA 505

B7KW13-B EQAAEDVDFNIVDAVRNDLVRIRADLFAFNVARGWDVGLGTLNQVRADLAASTNPYIRDA 505

Q13AU2-B SQPAEDVDFNIVDAVRNDLVRINADLFAFNVARGWDLGLGTLNQVRRDLAASTNPYVAES 494

Q07SX1-B SQPAEDVDFNIVDAVRNDLVRINADLFAFNVARGWDVGLGTLNQVRQDLAASTNPYVSEA 495

A3XF15-B EQPSEEVDLQVVDAVRNDLVRVSADLFAFNVARGRDVGLGTLNQVKADLAASDNRYISEA 501

: . :* :. ::* .

**Q88JT6-A** YMSWVDFADHLKHPASLINFIAAYGTHSSITGA-TTEAAKRAAAVALVLGGDG-----AP 692

**A5W572-A** YISWVDFADHLKHPASLINFIAAYGTHSSITGA-TTEAAKRAAAVALVLGGDG-----AP 692

**B0KJL7-A** YISWADFADHLKHPASLINFIAAYGTHSSITGA-TTEAAKRAAAVALVLGGAG-----AP 692

**Q88JT6-B** YTSWVDLVQHLKHPESLINFIAAYGTHSTITNA-TTLLEKRAAAMALVFGGDG-----AP 734

**A5W572-B** YSSWVDLVQHLKHPESLINFIAAYGTHSTITAA-TTLLEKRAAAMALVFGGDG-----AP 734

**B0KJL7-B** YTSWVDLVQHLKHPESLINFIAAYGTHSSITGA-TTLLEKRAAAMALVFGGEG-----AP 734

C7CGY0-A YTSWVEMAANLKNPLSVVNFIAAYGTHGTVVAA-TTLAAKRDAAMALVFGGEG-----AP 748

A9W3A5-A YTSWVEMAANLKNPLSVVNFIAAYGTHGTVVAA-TTLAAKRDAAMALVFGGEG-----AP 748

B7KW13-A YTSWVEMAANLKNPLSVVNFIAAYGTHGTVVAA-TTLAAKRDAAMALVFGGDG-----AP 748

Q13AU2-A YDHWVDLATNLKNPASIVNFVAAYGTHATIVGA-TTLEAKRMAAMELVFGVDQNGDATVA 740

Q07SX1-A YDHWVDFAANLKNPASIVNFVAAYGTHSTIAGA-TTLEAKRLAAMELVFGVDQDGDATVA 740

Q0G341-A YESWADLAQNLKTPMTVVNLIAAYGLHETVTGA-TTLADKRAAAFDLVFGSES----LND 605

A6E280-A YENWVELAANLKTPMTIVNLLAAYGTHSTILAA-NTLEEKRDAAFDLVFGGGG----VSD 653

Q1YMS2-A YDSWVDFAANLKNPMSVVNFIAAYGTHETIVAAGNNLQERRNAAMALVFNTEG-----AP 708

A3XF15-A YEGWSDYAANLKNPASIINFIAAYGTHDTIANA-TTVVQKRAAATDLVLGGDT-----AP 633

Q1YMS2-B VG---FAG-NLDPYASWADFQARNGLSDTIMDQ------MKVAYPDLILSTPEE-IAAFI 557

Q0G341-B ---------------------------------------------------------SMP 472

A6E280-B VD---MAG-DLSPYSSWADFQARNDVSDEDMAR------LMEAYPDLVLETPAQ-IAAFV 550

C7CGY0-B VG---FAGGDLSPYASWEDFQARNGLSDAVIAQ------FRQAYPDLVLAAAD--IAAFR 554

A9W3A5-B VG---FAGGDLSPYASWEDFQARNSLSDAVIAQ------FRQAYPDLVLAAAD--IAAFQ 554

B7KW13-B VG---FAGGDLSPYASWEDFQARNGLSDAVIAQ------FRQAYPDLVLAAAD--IAAFR 554

Q13AU2-B VG---FAGSNLTPYSSWEDFQQRNDLNNAVIAQ------FKQAYPDLTLAAAD--IAAFR 543

Q07SX1-B VG---FAGGDLSPYTSWEDFQQRNGLSQAVIEQ------FKQAYPDLQLAAAD--VAAFQ 544

A3XF15-B ID---LSDMSMTPYTDWEDFQARNGLSDEMIAK------FQTAYPALVLTVDTEQYDAFV 552

Insertion 5 (=C)

**Q88JT6-A** LDRLDFLNGTGAYANVTL**AGADGIAGTADDIAGVTVT**GVDAIDFWVGGLAEKKMPFGGML 752

**A5W572-A** LDRLDFLNGTGAYANVTL**AGADGIAGTADDIAGVTVT**GVDAIDFWVGGLAEKKMPFGGML 752

**B0KJL7-A** ADRLDFLNSTGAWANVTL**AGKDGIAGTADDIAGVTVS**GVDAIDFWVGGLAEKKMPFGGML 752

**Q88JT6-B** ADRMDFLNSSGAWANVTL**PGKDGVLGTADDLKAVTVT**GVDAIDLWIGGLAEAKAPFGGML 794

**A5W572-B** ADRMDFLNSSGAWANVTL**PGKDGVLGTADDLKAVTVT**GVDAIDLWIGGLAEAKAPFGGML 794

**B0KJL7-B** ADRLDFLNSSGAWANVTL**PGKDGVLGTADDLKAVTIT**GVDAIDLWIGGLAEEKTPFGGML 794

C7CGY0-A TDRLDYLNSRGSWAGRET-------------------GFGAVDLWIGGLAEKQMPFGGML 789

A9W3A5-A TDRLDYLNSRGSWAGRET-------------------GFGAVDLWIGGLAEKQMPFGGML 789

B7KW13-A TDRLDYLNSRGSWAGRET-------------------GFGAVDLWIGGLAEKQMPFGGML 789

Q13AU2-A ADRTAFLTGTGAWAGVET-------------------GLNRIDLWIGGLAEKKMPFGGML 781

Q07SX1-A ADRTAFLTGTGAWAGVET-------------------GLNRIDLWIGGLAEKKMPFGGML 781

Q0G341-A TDRLDFMLSRGEWNAANN-------------------GLNEIDLWVGGLAERIMPFGGML 646

A6E280-A ADRFDFLLGRNGWTSDTN-------------------GLNTIDLWVGGLAERIMPFGGML 694

Q1YMS2-A ADRLAFLNSTGGETAESV-------------------GLNDIDLWVGGLAEQILLFGGML 749

A3XF15-A ADRLDFVNGTGAWATIET-------------------GINAVEYWIGGLAEAIMPFGGML 674

Q1YMS2-B AVNPDIELTDGANGTKIV------------------KGIDRVDLWVGGLAEKHVLGG-MV 598

Q0G341-B TCSPSTSLAAGTSG------------------------------W--------------- 487

A6E280-B AVNPDVVLEDGANGAKIV------------------KGIDRVDLWTGGLAEKHVNGG-MV 591

C7CGY0-B AINGDIAIAMQADGTGVV------------------KGIDRLDLWVGGLAEKHINNG-VV 595

A9W3A5-B AINGDIAIAMQADGTGVV------------------KGIDRLDLWVGGLAEKHINNG-VV 595

B7KW13-B AINGDIAIAMQADGTGVV------------------KGIDRLDLWVGGLAEKHINNG-VV 595

Q13AU2-B EVNPDIAIAMQNDGTGIV------------------SGIDRLDLWVGGLAEKHINGG-LV 584

Q07SX1-B AINPDIDIAMNDDGTGIV------------------KGIDRVDLWVGGLAEQHINGG-LV 585

A3XF15-B EANPDIALIDNGDGTMTV------------------KGIDRVDLWVGGLAEQHIQDG-VV 593

*

**Q88JT6-A** GSSFNFVFETQLEALQNGDRFYYLSRTAGMNFGTELENN-SFAKLIMANSDVTHLS---- 807

**A5W572-A** GSSFNFVFETQLEALQNGDRFYYLSRTAGMNFGTELENN-SFAKLIMANSDVTHLS---- 807

**B0KJL7-A** GSSFNFVFETQLEALQNGDRFYYLSRTAGMNFGTELENN-SFAKLIMLNSDVTHLS---- 807

**Q88JT6-B** GSTFNFVFENQMEKLQDGDRFYYLERTAGLSMNAELESN-SFAKLIMANSSATHLP---- 849

**A5W572-B** GSTFNFVFENQMEKLQDGDRFYYLERTAGLSMNAELESN-SFAKLIMANSSATHLP---- 849

**B0KJL7-B** GSTFNFVFENQMEKLQDGDRFYYLERTSGLSMNAELESN-SFAKLIMANTSAAHLP---- 849

C7CGY0-A GSTFNAIFEAQMENLQDADRFYYLSRVQGQNFLNELEQN-SFSKIMLANSSLSLPG---P 845

A9W3A5-A GSTFNAIFEAQMENLQDADRFYYLSRVQGQNFLNELEQN-SFSKIMLANSSLSLPG---P 845

B7KW13-A GSTFNAIFEAQMENLQDADRFYYLSRVQGQNFLNELEQN-SFSKIMLANSSLSLPG---P 845

Q13AU2-A GSTFNAIFELQLENLQDGDRFYYLTRTQGQNFLNMLEQN-SFAKLIMANTDLAQPG---P 837

Q07SX1-A GSTFNAIFELQLENLQDGDRFYYLTRTQGQNFLNMLEQN-SFAKMIMANTDLAQPG---P 837

Q0G341-A GSTFSAIFEAQMEALQFGDRFYYLTRTQGQNLLNELEEN-AFAKIIMANTNLTLPG---P 702

A6E280-A GSTFTAIFEAQMEALQDGDRFYYLTRTQGQNFLNELEEN-SFSKMLLANTSLADPG---A 750

Q1YMS2-A GSTFAAIFEAQLEALQDGDRFYYLSRTQGLNLLNELENN-AFSKLIIANTDLSDPG---P 805

A3XF15-A GSSFGFAFQQQMEALQNGDRFYYLSRTNGMDMLGGLENN-SFASMIMRNTDIADGGAHIP 733

Q1YMS2-B GQTFWVVLHEQFDRLQEGDRFYYLERFDNFDFYDNFIDGQEFSDIIARNTGLTGLP---- 654

Q0G341-B ------------------------------------------------------------

A6E280-B GQTFWVVLHEQLDRLQEGDRFYYIDRFDNFDFYQEFGEDTTFASIVARNTSLTDID---- 647

C7CGY0-B GQTFWVVLHEQFDRLQDGDRFYYLERFDNFDFYENVVDGQGFSDIVARNTGLTVLP---- 651

A9W3A5-B GQTFWVVLHEQFDRLQDGDRFYYLERFDNFDFYENVIDGQGFSDIVARNTGLTVLP---- 651

B7KW13-B GQTFWVVLHEQFDRLQDGDRFYYLERFDNFDFYENLVDGQGFSDIVARNTGLTVLP---- 651

Q13AU2-B GETFWVVLSEQFERLQDGDRFYYISRFDNFDFYENFIDGQQFADIVTRNTGMTGIP---- 640

Q07SX1-B GQTFWVVLSEQFERLQDGDRFYYISRFDNFDFYENFIDGQEFADIIARNTGMTGLP---- 641

A3XF15-B GHTFWVLIHEQLDRLQEGDRFYYVDQIGDLPVYNNFISNLTFGDIVTRNTGMTDLP---- 649

**Q88JT6-A** NTVFLTPTFTLEVNQANQFTG-------LGADG-----KADPT 838

**A5W572-A** NTVFLTPTFTLEVNQANQFTG-------LGADG-----KADPT 838

**B0KJL7-A** NTVFLTPTFTLEVNQANQFTG-------LGADG-----KADPT 838

**Q88JT6-B** GLVFSDPGFYLELDQTKQYNEGLGSADPLGENGEQVVFRDSP- 891

**A5W572-B** GLVFSDPGFYLELDQTKQYNEGLGSADPLGENGEQVVFRDSP- 891

**B0KJL7-B** GLVFSDPGLYLELDQSKQYNDGLGHADPLGENGEQVVFRDSP- 891

C7CGY0-A DGIRGTADDIVPRHIGVDAFAD--YDFELEVNAA--------- 877

A9W3A5-A DGIRGTADDIVPRHIGVDAFAD--YDFELEVNAA--------- 877

B7KW13-A DGIRGTADDIVPRHIGVDAFAD--YDFELEVNAA--------- 877

Q13AU2-A DGIRGTADDIVPRHIGVDSFAN--YDYVLEVDES--------- 869

Q07SX1-A DGIRGTADDIVPRHIGVDSFAD--YDYVLEVD----------- 867

Q0G341-A DGIKGTEDDVTPHHIGIDVFAD--YDFVLEVN----------- 732

A6E280-A DGIRGTEDDVVRHHIGVDSFAR--YDFVLEVNQA--------- 782

Q1YMS2-A DGIRGTGDDVIARHIGVDAFGQ--YDYVLEVNKS--------- 837

A3XF15-A ANIFSSMEYILEVDQSVQAMADP-VSTELDPFLAAM------- 768

Q1YMS2-B EEIFRAN---DENDDTADNDDGVGDDTSDEDGDSDTV------ 688

Q0G341-B -------------------------------------------

A6E280-B NNLFDANGIDDDDDNATEDDNATEDDNATEDDD---------- 680

C7CGY0-B EHIFELSDEDG-PGTEPGDDDDDGDTDPVGGDP---------- 683

A9W3A5-B EHIFELSDEDG-PGTEPGDDDDDGDTDPVGGDP---------- 683

B7KW13-B EHIFELSDEDG-PGTEPGDDDDDD------------------- 674

Q13AU2-B EHMFQTDPID--QNENEGEGEGEEDGTPVGNGDPPTDDD---- 677

Q07SX1-B EHMFRTDPIDDENNQNPDDNEGDDDG----------------- 667

A3XF15-B QDVFSYTGDEIVEDNGTADQQTQQPPVTDDAGNANQAD----- 687
