# Supplementary material for: Identification of a Novel Calcium Binding Motif Based on the Detection of Sequence Insertions in the Animal Peroxidase Domain of Bacterial Proteins
Source: PLoS One. 2012 Jul 13;7(7):e40698. doi: 10.1371/journal.pone.0040698 (PMC3396595; doi:10.1371/journal.pone.0040698)
Supplement: Figure S2 — Alignment of bacterial ANP-like domains with G-x-D-G-x(5)-D-D insertions. Numbering starts from the terminal end of the ANP-like domain according to PS50292. Sequences in the alignment are in Text S3. In the alignment ANP-like domains of Arthrobacter sp. FB24 (A0JUB7), A. chlorophenolicus A6 / ATCC 700700 (B8HDW8), Cyanobium sp. PCC 7001 (B5IMQ8), Erythrobacter sp. SD-21 (A5PER4), Fulvimarina pelagi HTCC2506 (Q0G341), Leptothrix cholodnii ATCC 51168 / LMG 8142 / SP-6 (B1Y442), Manganese-oxidizing bacterium (strain SI85-9A1) (Q1YMS2), Mesorhizobium sp. BNC1 (Q11K84), Methylobacterium chloromethanicum CM4 /NCIMB 13688 (B7KW13, B7L1G6, B7KRS2), M. extorquens DSM 5838 / DM4 (C7CGY0), M. extorquens PA1 (A9W3A5), Nitrosomonas sp. AL212 (C6MFN2), Pseudomonas putida F1 (A5W572), P. putida GB1 (B0KJL7), P. putida KT2440 (Q88JT6), Rhodopseudomonas palustris BisA53 (Q07SX1), R. palustris BisB5 (Q13AU2), Roseovarius sp. TM1035 (A6E280), Roseobacter sp. AzwK-3b (A6FKA5, A6FV45), and Roseobacter sp. MED193 (A3XF15). Letters –A and -B refer to as N- and C-terminal ANP-like domains, respectively. The domain of human myeloperoxidase (P05164) does not present the insertion and is included in this alignment as an out-group member. The alignment was performed with CLUSTAL 2.1. In bold and underlined residues contacting catalytic Ca in human myeloperoxidase. (DOCX) [file pone.0040698.s002.docx]

Q1YMS2-B ---DDDTAGAGGVRELLGRNNNESHPEYG------AADEVFIRLTEARYGEYD------- 44

Q0G341-B ---DDDTEGAAGVRELLGRNNNENNPEFG------SADEVFIRLTEARYGEYD------- 44

A6E280-B ---DDDTEEATGVRTLSGEGNNEANPAYG------AAGEPFIRLTEARYGDPD------- 44

B7KW13-B PDNAGDDDVPTGYRELSGHGNNLDHPTWG------SADQAFIRLTQARYGEAD------- 47

C7CGY0-B PDNAGDDDVPTGYRELSGHGNNLDHPTWG------SADQAFIRLTQARYGETD------- 47

A9W3A5-B PDNAGDDDVPTGYRELSGHGNNLDHPTWG------SADQAFIRLTQARYGEAD------- 47

Q13AU2-B ---GDDDDGATGVRDLSGHNNNVANPNWG------SADQPFIRITNPHYGEAD------- 44

Q07SX1-B PANGDDDGGPTGVRELSGHNNNQANPNWG------AADQPFIRITNAHYGDED------- 47

A3XF15-B ---NEPADLIVGTRDLEGLTNNLLNPEISG-----GATLPFSRVTEARYAGIG------- 45

B8HDW8 --QLASPLLSHGLRTVDGSCNNLQPGQ----DTYGASDQVFPRLAPKAFGPAESGS---- 50

A0JUB7 --QVASPLLPYGLRTVDGTDNNLVAGQ----SGYGSASREFPRLSDPEWRTSSGG----- 49

B1Y442 --LVTSPLLPYGLRTVDGSHNNIVPGQ----EEFGAADRPMPNALQQFFRTAEMG--DVD 52

C6MFN2 -----DPLLPFGLRNVDGTYNNLEDGQ----SLFGSADQVFPRGLSIFLRPGEQITFDPD 51

A6FV45 ------------------------------------------------------------

A6FKA5 ------------------------------------------------------------

A3XF15-A ------------------------------------------------------------

A5PER4 ---LGNFHLPYGLRTVDGSYNNLVEGR----ERWGASDEIMPRMFTPNYRDDQDGDMMSF 53

Q11K84 ------------LEIAEG----------------------QAALFTPELVAAQQG----- 21

B7KW13-A --AIADPHVPYGLRTVDGTYNNLVPGR----ETWGSSGQPMPQLFEPTYLNDADGDTMAL 54

A9W3A5-A --AIADPHVPYGLRTVDGTYNNLVPGR----ETWGSSGQPMPQLFEPTYLNDADGDTMAL 54

C7CGY0-A --AIADPHVPYGLRTVDGTYNNLVPGR----ETWGSSGQPMPQLFEPTYLNDADGDTMAL 54

Q13AU2-A --AVADPKVPVGLRTVDGQDNNIVPGR----EQWGAADQSMPRLLTASYTTGAG--SIDL 52

Q07SX1-A --AIADPKVPAGLRTVNGEDNNIVPGR----EEWGAADQSMPRLLTSSYTTGAG--TLDL 52

Q1YMS2-A NDLVPDPHVPWGLRTVDGTYNNLVDGR----EQWGAADTVMPRYLDGSFVTDTN------ 50

Q0G341-A ------------------------------------------------------------

A6E280-A -------------TTVISSVTLPEPAD----GQWADLT---------------------- 21

B7L1G6 --LTQAHLLPYGLRTVDGSYNNLLPGR----ENWGASDQSFPGLFTPTYINDADGDRYDF 54

B7KRS2 --LTQAHLLPYGLRTVDGSYNNLLPGR----ENWGASDQSFPGLFTPTYINDADGDRYDF 54

Q88JT6-A ---LPNSQVPFGLRTVDGSYNNLVTGQ----SEFGAADNSFLRLLEASYR------ANYV 47

A5W572-A ---LPNSQVPFGLRTVDGSYNNLVTGQ----SEFGAADNSFLRLLEASYR------ANYV 47

B0KJL7-A ---LPNSQVPFGLRTVDGSYNNLVAGQ----SEFGAADNSFLRLLDASYR------ANYV 47

Q88JT6-B ---IPNVRAAMGLRAVDGSNNNLMNLNGNNNTQYGAADNVFPRVTDPVFNPAEGAPAGFF 57

A5W572-B ---IPNVRAAMGLRAVDGSNNNLMNLNGNNNTQYGAADNVFPRVTDPVFNPAEGAPAGFF 57

B0KJL7-B ---IPNIRAPLGLRAVDGSNNNLMNLNGHNNTQFGAADNVFPRLTDPVFNPAEGAPAGFF 57

B5IMQ8 ---------------ADSSTSNPAAVA--------------------------------- 12

P05164 ---GVTCPEQDKYRTITGMCNNRRSPTLG------ASNRAFVRWLPAEYEDGFS------ 45

Q1YMS2-B ---------------------------------GTTNNRAINPIFAGLDARTISNVLGHQ 71

Q0G341-B ---------------------------------ATINNRAVNPIFAGLDPREISNILGVQ 71

A6E280-B ---------------------------------ENGN-RQINPIFDGLDPRAISNILGPH 70

B7KW13-B ---------------------------------ANGN-RAINPIFDGLDARTISNILGTQ 73

C7CGY0-B ---------------------------------ANGN-RAINPIFDGLDARTISNILGTQ 73

A9W3A5-B ---------------------------------ANGN-RAINPIFDGLDARTISNILGTQ 73

Q13AU2-B ---------------------------------ANGN-LAINPVFDGLDPRTISNVLGSQ 70

Q07SX1-B ---------------------------------ANGN-RAINPVFDGLDPRTISNILGTQ 73

A3XF15-B ---------------------------------EDGA-GIVNPVFDDLDARAISNALGAQ 71

B8HDW8 ---FGGPPVATSYTQK-------------SGSVFDSRPRTISNLIADQTSTNPAAVAAAG 94

A0JUB7 ----------QNYESV-------------NANVTDNGPRFVSNVIVDQTATNPAAVAAAG 86

B1Y442 GPGPGGLVQTTYANAT-------------GGLVFDSQPRVISNLISDQTITNQAAVDVAT 99

C6MFN2 GPGPQTVGQSTHYNQT-------------KGAVFDSQPRTVSNLISDQTATNPAAIAVAN 98

A6FV45 ---------------------------------AAEGVEATKQVAVDTATSDLNALTAPG 27

A6FKA5 ---------------------------------AAEGVEATKQVAVDTATSDLNALTAPG 27

A3XF15-A ---------------------------------AAQDIETTKQSAFDTATTNLDALNAPG 27

A5PER4 GPGMT---ITNTDYGA-------------AGDVADVDPRIISNLIVDQTAGNPAAISAAL 97

Q11K84 -------------------------------------------LLDTAAATYG------ 31

B7KW13-A GPGAP--VITNTNYGL-------------PGSVADADPRIISNLVVDATLDNPAAIAAAL 99

A9W3A5-A GPGAP--VITNTNYGL-------------PGSVADADPRIISNLVVDATLDNPAAIAAAL 99

C7CGY0-A GPGAP--VITNTNYGL-------------PGSVADADPRIISNLVVDATLDNPAAIAAAL 99

Q13AU2-A NGPAPGGVVTGGNYAA-------------PGTIVDTAPRTVSNLIVDMSLNNPAAIIAAL 99

Q07SX1-A NGPAPGGAVTGGNYAG-------------PGTIVDTAPRTVSNLIVDMSLNNPAAIIAAL 99

Q1YMS2-A --SGAFFGVTNNNYAA-------------PGSVVDTDPRIISNLIVDMSVDNPAAVLAFL 95

Q0G341-A ----------------------------------QLAAHVGSELRVEISHTG-------- 18

A6E280-A -----------------------------LNSGPISAGLAGQTLRVEIQQTG-------- 44

B7L1G6 NPLPNVETWYSNNDYANAGVRSGSQPGPGSGTVIDADPRIISNLIVDQTLDNPAAIAAAL 114

B7KRS2 NPLPNVETWYSNNDYANAGVRSGSQPGPGSGTVIDADPRIISNLIVDQTLDNPAAIAAAL 114

Q88JT6-A GTGN----------------------------VVDSQPRTISNLIVDQTANNPAAVEAN- 78

A5W572-A GTGN----------------------------VVDSQPRTISNLIVDQTANNPAAVEAN- 78

B0KJL7-A GTGN----------------------------VVDSQPRTISNLIVDQTANNPAAVEAN- 78

Q88JT6-B GPGSPAIPGSSYQQTS--------------GPVFDSQPRTISNLIVDQTSNNPAAYATAY 103

A5W572-B GPGSPAIPGSSYQQTS--------------GPVFDSQPRTISNLIVDQTSNNPAAYATAY 103

B0KJL7-B GPGSPAIPGSSYQQTS--------------GPVFDSQPRTISNLIVDQTSNNPAAYATAY 103

B5IMQ8 ---------------------------------AAATQNSLLEAPVPMPGQNVNAFGVPL 39

P05164 -------------------------------LPYGWTPGVKRNGFPVALARAVSNEIVRF 74

Q1YMS2-B E----------------------------------------------------------- 72

Q0G341-B E----------------------------------------------------------- 72

A6E280-B D----------------------------------------------------------- 71

B7KW13-B E----------------------------------------------------------- 74

C7CGY0-B E----------------------------------------------------------- 74

A9W3A5-B E----------------------------------------------------------- 74

Q13AU2-B E----------------------------------------------------------- 71

Q07SX1-B E----------------------------------------------------------- 74

A3XF15-B D----------------------------------------------------------- 72

B8HDW8 FPARSQGN-TGVVPCTTDPDAEA------------VPPVAAAPEGCVPSHNTLDIPN--- 138

A0JUB7 KAHRTVNDGPTAVPCDGN----------------------GLPENCVPEGETLDIPN--- 121

B1Y442 RFA----DGEPVVISDEG---------------------------------TYFIQN--- 119

C6MFN2 GTP----GSELVNGTRADG----------------------------SAFQTYFIPN--- 123

A6FV45 -----AAEAALAAAQATAA------------EAQTTLDTLLTTHNITMDGNNVLLP---- 66

A6FKA5 -----AAEAALAAAQATAA------------EAQTTLDTLLTTHNITMDGNNVLLP---- 66

A3XF15-A -----AADAALAAAQAIAT------------EAQTTLDTLLSTHAITMDGNNVMLP---- 66

A5PER4 TQT--GYEGDATAAIQALHSAWADHEN-GLMTYEEFATLALDTYGLEMQGDTLVIP---- 150

Q11K84 -------------------------------------------LEFEMDGKTIKLP---- 44

B7KW13-A RIA--GSENVIADQRAITAAHEA-------LKAAQAANPAGDHAVLQSNLDALLEQTGVT 150

A9W3A5-A RIA--GSENVIADQRAITAAHEA-------LKAAQAASPAGDHAVLQSNLDALLEQTGVT 150

C7CGY0-A RIA--GSENVIADQRAITAAHEA-------LKAAQAANPAGDHAVLQSNLDALLEQTGVT 150

Q13AU2-A TFA--GSEDVLADQSEITAAFLA-------LKAARDADPLGDHAALQLELDTILEQKGVT 150

Q07SX1-A TFA--GSGDVLGDQGEITAAFLA-------LKAARDADPLGDHATLQQALDDVLEQKGVT 150

Q1YMS2-A N-----NELAVETFKELHGGLEP-------VAPGTVVNSATQLAVTDADLALIP------ 137

Q0G341-A -----GGQALIDN-------------------VELSASSGNRIEITDEDLATLP------ 48

A6E280-A -----GSQVLVDN-------------------VALSTSNGNEIEIDNIDLATIP------ 74

B7L1G6 THAGLAGQPLMTALSEIVGAHNAAKTTPNDPAATAALDAKLAQYGVEMDGNTVFLP---- 170

B7KRS2 THAGLAGQPLMTALSEIVGAHNAAKMTPDDPAAIAALGAKLVQYGVEMDGNTVFLP---- 170

Q88JT6-A ------------------------------GGAAPVMSPGIDGVFGTADDKPVFFIP--- 105

A5W572-A ------------------------------GGAAPVMSPGIDGVFGTADDKPVFFIP--- 105

B0KJL7-A ------------------------------GGAAPVMSPGIDGVFGTADDKPVFFIP--- 105

Q88JT6-B DPGADGVLNFGAAGNDDVLK----------DGVRIVASPGMDGQFGTTDDHDVYLFE--- 150

A5W572-B DPGADGVLNFGAAGNDDVLK----------DGVRIVASPGMDGQFGTTDDHDVYLFE--- 150

B0KJL7-B DPGADGVLNFGAPGNDDVLK----------DGVRIVASPGMDGQFGTTDDHDVYLFE--- 150

B5IMQ8 G-----------------------------------DDGQELGIENFGGGDTIEIRN--- 61

P05164 P----------------------------------------------------------- 75

Q1YMS2-B -----------ADLSPAASGANTFFMAFGQYFDHGLDFLPKNSANGVLAIGGPGTSRAPG 121

Q0G341-B -----------ADLAPAKSGANTFFMAFGQYFDHGLDFLPKDSLNGVIEIGGPGSARAPG 121

A6E280-B -----------DTTAPNAMNASALFMAFGQYFDHGLDFIAKNPAFGTIEIGGPGAERSPT 120

B7KW13-B -----------AGLPKAGNDANIFFMAMGQYIDHGLDFLPKG-GNGSIVIGAPGG-GAPG 121

C7CGY0-B -----------AGLPKAGNDANIFFMAMGQYIDHGLDFLPKG-GNGSIVIGAPGG-GAPG 121

A9W3A5-B -----------AGLPKAGNDANIFFMAMGQYIDHGLDFLPKG-GNGSIVIGAPGG-GAPG 121

Q13AU2-B -----------AGLPSAGNDANIFFMAMGQYIDHGLDFLGKG-GNGTIQIGALGG-GAPG 118

Q07SX1-B -----------ANLPHAGNDANIFFMAMGQYIDHGLDFLGKG-GNGSIQIGAAGG-GAPG 121

A3XF15-B -----------ADAAKAAS-ANMFMMSFGQYFDHGLTFIPKG-GHDPITIGGADM-GRPS 118

B8HDW8 ----------VTTDVGLSPPYNSLFTLFGQFFDHGIDQTVKG-GGTVYVPLKADDPLIAG 187

A0JUB7 ----------VTTDFGLSPPYNGMFALFGQFFDHGVDFTKKT-KNYVMMPLSPDDPLYVP 170

B1Y442 ----------VAPDEGLSAPFNSWFTIFGQFFDHGLDLIGKG-AGTVFIPLKPDDPLIAG 168

C6MFN2 ----------VTPDAGLSAPYNSWFTFFGQFFDHGLDLVNKGQSGTVFVPLLDDDPLVTG 173

A6FV45 ---------DVTPDEGLSAPYNSWMTLFGQFFDHGLDLVGKGGSGTVYIPLQPDDPLYDA 117

A6FKA5 ---------DVTPDEGLSAPYNSWMTLFGQFFDHGLDLVGKGGSGTVYIPLQPDDPLYDA 117

A3XF15-A ---------DVTPDEGLSAPYNSWMTLFGQFFDHGLDLVGKGGSGTVYIPLQPDDPLYDA 117

A5PER4 ---------NTAPDEGLSSPFNAWMTYFGQFFDHGLDLIPKADNGTVYVPLQPDDPLFDA 201

Q11K84 ---------NVAPDEGLSAPFNGWMTFFGQFFDHGLDLISKGGNGTIYVPLEPDDPLYVP 95

B7KW13-A VTNGSIDVLNVSPDEGLSKPFNAWMTFFGQFFDHGLDLISKGGNGTVYVPLAADDPL-VL 209

A9W3A5-A VTNGSIDVLNVSPDEGLSKPFNAWMTFFGQFFDHGLDLISKGGNGTVYVPLAADDPL-VL 209

C7CGY0-A VTNGSIDVLNVSPDEGLSKPFNAWMTFFGQFFDHGLDLISKGGNGTVYVPLAADDPL-VL 209

Q13AU2-A VTNGSIDVPNVAPDEGLSAPFNAWMTFFGQFFDHGLDLISKGGAGTVYVPLAADDPLRTH 210

Q07SX1-A VTNGSIEVPNVAPDEGLSAPFNAWMTFFGQFFDHGLDLISKGGAGTIYVPLAADDPLRTH 210

Q1YMS2-A ---------NIAPDEGISAPFNGWTTFFGQFFDHGLDLITKGTNGTVYIPLQPDDPLYVP 188

Q0G341-A ---------NIAPDDGISAPFNAWMTFFGQFFDHGLDLITKGGNGTVFIPLQADDPLVVS 99

A6E280-A ---------NIAPDDGISAPFNAWMTFFGQFFDHGLDLITKGDNGTVFIPLQADDPLVLG 125

B7L1G6 ---------NVSPDEGLSSPFNGWMTIFGQFFDHGLDLVAKGGNGTVYVPLSPDDPLYVP 221

B7KRS2 ---------NVSPDEGLSSPFNGWMTIFGQFFDHGLDLVAKGGNGTVYVPLSPDDPLYVP 221

Q88JT6-A ---------NVSPDAGLTAGFNAWMTFFGQFFDHGLDLVSKSSTDIVFIPLRPDDPLFVA 156

A5W572-A ---------NVSPDAGLTAGFNAWMTFFGQFFDHGLDLVSKSSTDIVFIPLRPDDPLFVA 156

B0KJL7-A ---------NVSPDVGLTAGFNAWMTFFGQFFDHGLDLVTKSSTDIVFIPLRPDDPLYNA 156

Q88JT6-B ---------NTAADAGLSAPFNAWMTFFGQFFDHGLDLVTKGGSGTIYIPLQPDDPLYVE 201

A5W572-B ---------NTAADAGLSAPFNAWMTFFGQFFDHGLDLVTKGGSGTIYIPLQPDDPLYVE 201

B0KJL7-B ---------NTAADAGLSAPFNAWMTFFGQFFDHGLDLVTKGGSGTIYIPLQPDDPLYVE 201

B5IMQ8 ----------VAPLP-INAPYNSWLTLFGQGFDHGLDFINKGGNGNVFIPLDPSDSLFDA 110

P05164 -----------TDQLTPDQERSLMFMQWGQLLDHDLDFTPEPAARASFVTGVNCETSCVQ 124

. ** :**.: :

Q1YMS2-B VDNP-------------------------------------ADLTRGEVYTIDEN----- 139

Q0G341-B VDNP-------------------------------------ADLTRGKVHVIDEN----- 139

A6E280-B SDNP-------------------------------------ADLTRAEVAGYDED----- 138

B7KW13-B SNNP-------------------------------------ADLTRGTVMAVDAN----- 139

C7CGY0-B SNNP-------------------------------------ADLTRGTVMAVDAN----- 139

A9W3A5-B SNNP-------------------------------------ADLTRGTVMAVDAN----- 139

Q13AU2-B SGNP-------------------------------------ADLTRGSVASYDAN----- 136

Q07SX1-B SDNP-------------------------------------ADLTRGSVAGYEN------ 138

A3XF15-B GDNP-------------------------------------ADLTRATATINPET----- 136

B8HDW8 PDHDFGT----------ADDLNPHLRFMVLTRGQNQ---PGQDGILGTADDLQ------- 227

A0JUB7 GGRTN---------------------FMLLNRAENQ---PGPDGVLGTADDEQ------- 199

B1Y442 DDGILGN----------ADDLPPQLQFMALSRAAASNIAPGADGVVGTSDDVR------- 211

C6MFN2 PDGILGDDPLTVGVDESLDDLPEGLRFMALTRATNK---PGNDGIMGTGDDVR------- 223

A6FV45 NSPTN---------------------FMVLTRATNQ---PGPDGILGTADDIR------- 146

A6FKA5 NSPTN---------------------FMVLTRATNQ---PGPDGILGTADDIR------- 146

A3XF15-A TSPTN---------------------FMVLTRATNQ---PGPDGILGTADDVR------- 146

A5PER4 GPDG----------------------IPFTADDGRT---NFMVMTRAQIDEN-------- 228

Q11K84 GGHANFM-----------------VLTRATQFDGPG---PDGVLG-TADDTS-------- 126

B7KW13-A GQDG--------------------LAGTADDLAPHL---RFMTLTRAAQVE--------- 237

A9W3A5-A GQDG--------------------LAGTADDLAPHL---RFMTLTRAAQVE--------- 237

C7CGY0-A GQDG--------------------LAGTADDLAPHL---RFMTLTRAAQVE--------- 237

Q13AU2-A GPDG--------------------IAGTGDEVPGQM---AFMALTRATPAAD-------- 239

Q07SX1-A GPDG--------------------VAGTGDEVSEQM---AFMALTRATPASD-------- 239

Q1YMS2-A GGFTNF------------------MVLTRAAKAEHL---PGEDGVLGTADDI-------- 219

Q0G341-A G-----------------------------QVPPHM---QFMVLTRSTPTEGP------- 120

A6E280-A ADGI---------------------AGTADDLPNHL---RFMALTRSTPVDGPGADGVLG 161

B7L1G6 GGQN---------------------YIPLTRVTVEA---G-ADGILGTADDG-------- 248

B7KRS2 GGQN---------------------YIPLTRVTVEA---G-ADGILGTADDG-------- 248

Q88JT6-A GSPTN---------------------FMVLSRAVRT---AGADGVVGTADDS-------- 184

A5W572-A GSPTN---------------------FMVLSRAVRT---AGADGVVGTADDS-------- 184

B0KJL7-A SSPTN---------------------FMVLSRAVRT---AGADGVVGTADDG-------- 184

Q88JT6-B GGFTN---------------------FMVVTRATNL---PGPDGILGNADDIR------- 230

A5W572-B GGFTN---------------------FMVVTRATNL---PGPDGILGNADDIR------- 230

B0KJL7-B GGFTN---------------------FMVVTRATNL---PGPDGILGNADDIH------- 230

B5IMQ8 GADG-----------------------IPGTADDGP---NFMVVTRATRDFANG------ 138

P05164 QPPCFP------------------------------------------------------ 130

Q1YMS2-B ----GVPQHLNKASPFVDQNQAYGSNALVGQFLRESDGD--------------------Q 175

Q0G341-B ----GIPQHLNKASPFVDQNQAYGSNELVGQFLRESDGA--------------------Q 175

A6E280-B ----GVPQHTNMTSPFVDQNQAYGSHELVGQFLRESDGA--------------------H 174

B7KW13-B ----GVPQHKNQTSPYIDQNQAYGSNALVGQFLRESDGA--------------------Q 175

C7CGY0-B ----GVPQHKNQTSPYIDQNQAYGSNALVGQFLRESDGA--------------------Q 175

A9W3A5-B ----GVPQHKNQTSPYIDQNQAYGSNALVGQFLRESDGA--------------------Q 175

Q13AU2-B ----GVPQHINRTSPYVDQNQAYGSNDLVGQFLRAGDDN--------------------G 172

Q07SX1-B ----GVPQHVNRTSPYVDQNQAYGSNDLVGQFLREGDGN--------------------G 174

A3XF15-B ----GEIEHTNITSPVVDQNQVYGSSNLVGQLLRESGSN--------------------G 172

B8HDW8 -------DALNTNSPWVDQSQTYASHSSHQVFLREYTNN--------------------- 259

A0JUB7 -------NATNTDSPWVDQSQTYSSHSSHQVFLREYTLN--------------------- 231

B1Y442 -------TFSNSTTPWIDQNQTYTSHASHQVFLREYTLV--------------------- 243

C6MFN2 -------DHVNQTTPFVDQNQTYTSHPSHQVFLREYELN--------------------- 255

A6FV45 -------EHFNKTTPWVDQNQTYTSHPSHQVFLREYDID--------------------- 178

A6FKA5 -------EHFNKTTPWVDQNQTYTSHPSHQVFLREYDID--------------------- 178

A3XF15-A -------EHFNKTTPWVDQNQTYTSHPSHQVFLREYDLD--------------------- 178

A5PER4 ------GETMNTTTPFVDQNQTYGSHASKQVFMREYEL---------------------- 260

Q11K84 ------REQINTTTPFVDQNQTYTSHPSHQVFLREYAM---------------------- 158

B7KW13-A ------GSQRNVTTPFVDQNQTYTSNASHQVFLREYAL---------------------- 269

A9W3A5-A ------GSQRNVTTPFVDQNQTYTSNASHQVFLREYAL---------------------- 269

C7CGY0-A ------GSQRNVTTPFVDQNQTYTSNASHQVFLREYAL---------------------- 269

Q13AU2-A ------GSQVNTTTPFVDQNQTYTSHASHQVFLREYKM---------------------- 271

Q07SX1-A ------GSQTNTTTPFVDQNQTYTSHASHQVFLREYSM---------------------- 271

Q1YMS2-A ------VSHTNTTTPFVDQNQTYTSHASHQVFLREYKFN--------------------- 252

Q0G341-A --DGSMTEGKNVTTPFVDQNQTYTSHASHQVFVREYEMV--------------------- 157

A6E280-A TADDTQHEGQNTTTPFVDQNQTYTSHASHQVFLREYAFD--------------------- 200

B7L1G6 ------AGPKNLTTPWVDQNQTYASTASKQVFMREYIPG--------------------- 281

B7KRS2 ------AGPKNLTTPWVDQNQTYASTASKQVFMREYIPG--------------------- 281

Q88JT6-A --------QPNTTSPFVDQSQTYSSHPSHQVFLREYTVN--------------------- 215

A5W572-A --------QPNTTSPFVDQSQTYSSHPSHQVFLREYTVN--------------------- 215

B0KJL7-A --------QPNTTSPFVDQSQTYSSHPSHQVFLREYMLD--------------------- 215

Q88JT6-B -------EHTNTTTPFVDQNQTYSSHPSHQVFLRAYVMT--------------------- 262

A5W572-B -------EHTNTTTPFVDQNQTYSSHPSHQVFLRAYVMT--------------------- 262

B0KJL7-B -------EHTNTTTPFVDQNQTYSSHPSHQVFLRAYVMT--------------------- 262

B5IMQ8 ------GATQNVTTPWIDMNQAYGSHASYQVFLRDYTVNSTFGSDGDPGFSPISGLQLNQ 192

P05164 -------------LKIPPNDPRIKNQADCIPFFRSCPACP-------------------- 157

. . :.*

Q1YMS2-B GVGMRLLSGATDP------------------------------STPDFNLLPTLRELIAH 205

Q0G341-B GFGMRLLAGADDP------------------------------SNPEFRLLPTLRELIEH 205

A6E280-B GFGMRLLGGEADP------------------------------SDPAFTLLPTLRDLILH 204

B7KW13-B GVGMRLLAGAPDP------------------------------SNPAFNLLPTLRELVNH 205

C7CGY0-B GVGMRLLAGAPDP------------------------------SNPAFNLLPTLRELVNH 205

A9W3A5-B GVGMRLLAGAPDP------------------------------SNPAFNLLPTLRELVNH 205

Q13AU2-B GLGAHLFAGAPDP------------------------------SNPEFALLPTLRELITE 202

Q07SX1-B GVGAHLFAGGPDP------------------------------SNPQFSLLPTLRELIEE 204

A3XF15-B GFGAHVLMGQEDP------------------------------SASGFQLMATLRELLDH 202

B8HDW8 PEGRPVSTGGLLGGPAGTAAA----------------GGMATWADTKKQAREMLGIQLLD 303

A0JUB7 ENGDPVSTGELIEGEP---------------------GGMATWARIKEQARTMLGLELSD 270

B1Y442 G-GRPESTGRLLDGAVEGS--------------------IGNWAEVKAQAL-MLGIRLTD 281

C6MFN2 GAGRPVSTGNLLEDSTTGDG-------------------LATWADIKAQAQNMLGINLTD 296

A6FV45 ANGNPVSNGYLLH--------GQT-------------GGMATWGDVKAQAATKLGIQLND 217

A6FKA5 ANGNPVSNGYLLH--------GQT-------------GGMATWGDVKAQAATKLGIQLND 217

A3XF15-A ANGSPVSNGYLLH--------GQS-------------GGMSTWGDVKAQAAAKLGIQLND 217

A5PER4 VNGRPEATGHLLE--------GVN-------------GGLATWADVKAQALDVLGIVLSD 299

Q11K84 VDGKPLATGHMLD--------GAN-------------GGLPTWADIKAQAKNLLGIELTD 197

B7KW13-A VDGRPVATGRLLG--------GAD-------------GGLATWADVKFQARTILGIELTD 308

A9W3A5-A VDGRPVATGRLLG--------GAD-------------GGLATWADVKFQARTILGIELTD 308

C7CGY0-A VDGRPVATGRLLG--------GAD-------------GGLATWADVKFQARTILGIELTD 308

Q13AU2-A VGGVPMATGKLLG--------GAE-------------GGLATWADVKAQAQNMLGIVLSD 310

Q07SX1-A ASGVPMATGKLLG--------GAD-------------GGLATWADVKAQARDALGIELSD 310

Q1YMS2-A ADGEPVSTGRLLD--------GLE-------------GGLATWGQIKAEAAAKLGIALDD 291

Q0G341-A D-GRPISTGHLLD--------GAN-------------GGLATWAEVKVQAAEKLGIALAD 195

A6E280-A TNGNPVSTGKLLD--------GAN-------------GGIPTWAEVKAQARDLLGIELTD 239

B7L1G6 PDGKPIASGHLLE--------GSN-------------GGLATWADIKAQAKTVLGIELTD 320

B7KRS2 PDGKPIASGHLLE--------GST-------------GGLATWADIKAQAKTVLGIELTD 320

Q88JT6-A AAGDPVATGRLITNRDL----GADGKFGTADDGNGESGGMATWAVVKAQARDLLGINLTD 271

A5W572-A AAGEPVATGRLITNRDL----GADGKFGTADDGNGESGGMATWAVVKAQARDLLGINLTD 271

B0KJL7-A AAGDPVATGRLITNRDL----GADGKFGTADDGNSENGGMATWAVVKAQARDLLGINLTD 271

Q88JT6-B DDG-PVATGRLITNRDL----GADGRFGTADD--TEIGGMATWKVVKAQARDLLGINLTD 315

A5W572-B DDG-PVATGRLITNRDL----GADGRFGTADD--TEIGGMATWKVVKAQARDLLGINLTD 315

B0KJL7-B DDG-PVATGRLITNRDL----GADGKFGTADD--TEIGGMATWKVVKAQARDLLGINLTD 315

B5IMQ8 EFGAVISTGNMLASTRTG-------------------SGLPTWADIKANAESMLGFVLTD 233

P05164 --GSNITIRNQIN-------------------------------------------AL**T**S 172

* :

Q1YMS2-B HWENDTIFVDPSLPGGSVSFR-DYFTDYPISDNATGSIFDEATGAYD------------- 251

Q0G341-B HWEANTIFRDPSLPNGAISFR-EYFTDFPISEGVTGNLFDEATGAYD------------- 251

A6E280-B HWEAETYFEDPGLPGGAARLQ-DVFPD----------LVDEITGEID------------- 240

B7KW13-B HWQADTIFAG---PDGPISFR-TYYTNFALSEGVTGTLFNTETGAFD------------- 248

C7CGY0-B HWQADTIFAG---PDGPISFR-TYYTNFALSEGVTGTLFNTETGAFD------------- 248

A9W3A5-B HWQADTIFAG---PDGPISFR-TYYTNFALSEGVTGTLFNTETGAFD------------- 248

Q13AU2-B HWQNNTVFHSSSLQGGSVAFR-DYFAGLVG-----------QNGVIN------------- 237

Q07SX1-B HWSNNTVFHSESLPDGAVAFR-DYFPGLV------------QGGVIN------------- 238

A3XF15-B HTQAGTVFTDT--DKGDVTLE-GYYPDLFN-----------EDGTYN------------- 235

B8HDW8 KDALNVPLLAADAYGKFIPGPKDGLPQFVTRSG-----LVEADRTANGGNGTLVPQD--- 355

A0JUB7 VDVADIPKLATDQYGRFLRGP-NGLPQYETATG-----RVEGNLAAP----VAPPAN--- 317

B1Y442 ADIGNVPVLLTDEYGKFIPGS-NGYAQLVMANGS----IVQGTAAG-----ILTTG---- 327

C6MFN2 ADLTNLPLLATDTYGKFIPGS-NGFVQIVTSSG-----LVQGNPAANGGNGIDIPGN--- 347

A6FV45 SDVLDGPLLATDPYGNFIPGA-NGLPQLVVPNP------AYVEGGTEPL-NILIEGDLAN 269

A6FKA5 SDVLDGPLLATDPYGNFIPGA-NGLPQLVVPNP------AYVEGGTEPL-NILIEGDLAN 269

A3XF15-A SDVLDGPLLATDAYGNFIPGA-NGLPQLVVANP------AYVEGGTEPL-NILIEGDLAN 269

A5PER4 ADVLALPLIRSDPYGNFIPDE-NGYPQVVVGIG------ADGIPNTAD--DVVVSGSPTN 350

Q11K84 RDVFNIPLIRTDLYGEFIRDE-NGFPQVIVGIG------PDGIPNTDD--DIVMSGTPDA 248

B7KW13-A ADVSAVPQLLVDAYGEFVRSA-NGLPQVMVGVG------PGGQAVYAS--GSLAEPLKLS 359

A9W3A5-A ADVSAVPQLLVDAYGEFVRSV-NGLPQVMVGVG------PGGQAVYAS--GSLAEPLKLS 359

C7CGY0-A ADVSAVPQLLVDAYGEFVRSA-NGLPQVMVGVG------PGGQAVYAS--GSLAEPLKLS 359

Q13AU2-A LDVLNVPLLRTDAYGEFIRDA-NGFAQVIIGLG------DDGIPNTAD--DLVASGTNLA 361

Q07SX1-A LDVFNVPLLRTDPYGEFIRDA-NGFPQVIVGIG------ADGIPNTAD--DIVASGTNLA 361

Q1YMS2-A QDALNIPLLRTDPYGEFIRGD-NGLPMIVTGLG------PDGIPNTAD--DIVVQGN--- 339

Q0G341-A GDAVSIPLILTDAYGEFVRGP-NGFPQVVLGVG------PDGIPNTAD--DIVVEGDPEN 246

A6E280-A GDVLNIPLLRTDDYGEFIRGP-NGLPQIVVGIG------ADGIPNTAD--DDVVEGNLAA 290

B7L1G6 LNVGNIPLVAADPYGNFIPGP-NGYPQLVVGMG------PDSLLGTAD--DVLREGNPAA 371

B7KRS2 LNVGNIPLVAADPYGNFIPGP-NGYPQLVVGMG------PDGLLGTVD--DVLREGNPAA 371

Q88JT6-A ADVHSVPLLATDAYGNFLRGP-NGMPQVVMRVNN----GADGIAGTADDVTQLVEGDRNA 326

A5W572-A ADVHSVPLLATDAYGNFLRGP-NGMPQVVVRVNN----GADGIAGTADDVTQLVEGDRNA 326

B0KJL7-A ADVHSVPLLATDAYGNFLRGP-NGMPQVVMRVNN----GADGIAGTADDVTTLVEGNRAA 326

Q88JT6-B ADVDNVPLLATDAYGNFIKGP-NGYPMVVMKG-------LDGIAGTADD--QEVEGNPLA 365

A5W572-B ADVDNVPLLATDAYGNFIKGP-NGYPMVVMKG-------LDGIAGTADD--QEVEGNPLA 365

B0KJL7-B ADVDNVPLLATDAYGNFIKGP-NGYPMVVMKG-------VDGIAGTADD--QQVEGNPLA 365

B5IMQ8 DFVLSSPLLATDDYGRFIPGA-SGLPQVVVETAPGVFTLVEGNLTTPVDLSAIPGGGT-- 290

P05164 FV**D**A**S**MVYGSEEPLARNLRNMSNQLGLLAVNQR--------------------------- 205

Q1YMS2-B ----------------------------PDVVASMVSNFMGGGYP--------------- 268

Q0G341-B ----------------------------PDVVNHLVSDFMGGGYP--------------- 268

A6E280-B ----------------------------PDMVQALASDFLGSGQP--------------- 257

B7KW13-B ----------------------------PQVLTKLVGNFMGSGHP--------------- 265

C7CGY0-B ----------------------------PQVLTKLVGNFMGSGHP--------------- 265

A9W3A5-B ----------------------------PQVLTKLVGNFMGSGHP--------------- 265

Q13AU2-B ----------------------------QALLPSMISNFMGTNHA--------------- 254

Q07SX1-B ----------------------------TAMLPGMISNFMGTSHA--------------- 255

A3XF15-B ----------------------------AATIKDLSDDFMGEGWP--------------- 252

B8HDW8 ---------------------------ILYFNTPFLTDIAHNADPSPQDT-DHNPATPPA 387

A0JUB7 ---------------------------VERIGIAFLDDIGHNAAP-------FNSQTG-A 342

B1Y442 ---------------------------SAKTGHAFLDDIAHHAVPGFVDT-NRDGIQNNG 359

C6MFN2 ---------------------------AVRTGHAFLDDIAHTAVPKFAS----------G 370

A6FV45 PVDASQ---------------------ALRNGHAFLEDISHTAFPKGMIDHDRNPMTPEI 308

A6FKA5 PVDASQ---------------------ALRNGHAFLEDISHTAFPKGMIDHDRNPMTPEI 308

A3XF15-A PVDASQ---------------------AVRNGHAFLEDIAHNAVP-GTYVVDR-FTGETA 306

A5PER4 PVHLLTGMAGEIPPEGVPEGM---AVIPVRTAHAFLDDIAHNAVP----------VFDEG 397

Q11K84 PLKLN-------PADGGTE--------PVRTSHAFLDDIAHNAVP----------VLDTQ 283

B7KW13-A AIQLPVGTVLVGPNGAQNVIEAGETVAAARTFNAFLDDIAHNAVP----------VAVNG 409

A9W3A5-A AIQLPVGTVLVGPNGAQNVIEAGETVAAARTFNAFLDDIAHNAVP----------VAVNG 409

C7CGY0-A AIQLPVGTVLVGPNGAQNVIEAGETVAAARTFNAFLDDIAHNAVP----------VAVNG 409

Q13AU2-A PLNLATLNNGAGP---------------VRTSHAFLDDIAHNAAP----------VVVDG 396

Q07SX1-A PFDLASLNGGLGP---------------VRTSHAFLDDIAHNAAP----------VVVGG 396

Q1YMS2-A -LTTPVNTMAIGA---------------IRIGHAFLDDIAHNAAP----------VINGG 373

Q0G341-A PINTFTVQSEDGS-----------LTGAVRIGHAFLDDIAHAANP----------VDSQT 285

A6E280-A PVNTFTA-------------------GAIRIGHAFLDDIAHAANP----------FDSQS 321

B7L1G6 PVSAQG---------------------VVLTGHAFLDDIAHAAVP----------VIAGG 400

B7KRS2 PVSAQG---------------------VVLTGHAFLDDIAHAAVP----------VIAGG 400

Q88JT6-A PISLAN---------------------AVSTGHGFLDDIAHNAAP----------VVVGG 355

A5W572-A PISLAN---------------------AVSTGHGFLDDIAHNAAP----------VVVGG 355

B0KJL7-A PISLAN---------------------AVSTGHGFLDDIAHNAEP----------VKVGG 355

Q88JT6-B PLDLTN---------------------AVRTGHQFLADIAHNAVP----------VFSGG 394

A5W572-B PLDLTN---------------------AVRTGHQFLADIAHNAVP----------VFSGG 394

B0KJL7-B PISLTN---------------------AVRTGHQFLADIAHNAVP----------VFSGG 394

B5IMQ8 ---------------------------VVRTGHAFLLDIAHPANP--------------- 308

P05164 ---------------------------FQDNGRALLPFDNLHDDP--------------- 223

: .

Q1YMS2-B -LLLDTNPYIN---------------------------------LLDHYVAGDGRANENF 294

Q0G341-B -LLLDTNPFIN---------------------------------LLDHYIAGDGRANENF 294

A6E280-B -LLLDANPFID---------------------------------LLDHRMAGDGRANENF 283

B7KW13-B -LLLDTNPFIS---------------------------------VLDHFVAGDGRANENF 291

C7CGY0-B -LLLDTNPFIS---------------------------------VLDHFVAGDGRANENF 291

A9W3A5-B -LLLDTNPFIS---------------------------------VLDHFVAGDGRANENF 291

Q13AU2-B -LLLDANPFIN---------------------------------VLDHYVSGDGRTNENF 280

Q07SX1-B -LVLDANPFIN---------------------------------VLDHYVAGDGRANENF 281

A3XF15-B -LLIDTNPFMN---------------------------------LLDHFVGGDGRANENV 278

B8HDW8 APAPDADNTAS------ADFAA------------QAPGTYDDEMLGAHFIAGDGRVNENI 429

A0JUB7 PLQPDDDEDVN------GVNEP------------RPAGRYDDEMLDKHFVAGDGRVNENI 384

B1Y442 ELSQTPDTDTG------DVNLDGVVNEADLTADDRIAGTYDNELLDRHFITGDGRGNENI 413

C6MFN2 LLNPDPDPDTG------NTPAP---------------GTYDDELLDAHFITGDGRGNENI 409

A6FV45 EVLPDADTDTG------NAIMPN---------IFGMNETYDNELLDRHFIAGDGRGNENF 353

A6FKA5 EVLPDADTDTG------NAIMPN---------IFGMNETYDNELLDRHFIAGDGRGNENF 353

A3XF15-A TKQADADTDTG------NAIIPN---------QFGQNETYDNELLDRHFIAGDGRGNENF 351

A5PER4 NLAADADTDT-----GNAVATG----------TRGENLEYDNELLDRHFITGDGRGNENI 442

Q11K84 GFLRPDGVDLPVDPAGNAVQFDP---------LTGHNLEYDNELLDRHFITGDGRGNENV 334

B7KW13-A VLRPDADALT-----GNAVQMNP---------QTGRNLEYDNELLDRHFVTGDGRGNENI 455

A9W3A5-A VLRPDADALT-----GNAVQMNP---------QTGRNLEYDNELLDRHFVTGDGRGNENI 455

C7CGY0-A VLRPDADALT-----GNAVQMNP---------QTGRNLEYDNELLDRHFVTGDGRGNENI 455

Q13AU2-A VLTPDTDSAT-----GNDVAINP---------LTGQRLEYDDELLDRHYITGDGRGNENI 442

Q07SX1-A VLAPDADSAT-----GNDVAINP---------LTGQRLEYDNELLDRHYITGDGRGNENI 442

Q1YMS2-A VLQPDADVLT-----GNTVAS-----------QQGQNTEYDNELLDRHYITGDGRGNENI 417

Q0G341-A GLLK-------------------------------AEGTYDSELLGRHFITGDGRGNENI 314

A6E280-A GMLKTADDDT-------AVGLSD---------SVSTAGTYDNELLDRHFVTGDGRGNENI 365

B7L1G6 VLQADGDAALG-YASADGAPGPQ---------GPRGATAYDNELLDRHFVAGDGRANENI 450

B7KRS2 VLQADGDAALG-YANVDGTPGPQ---------GPRGATAYDNELLDRHFVAGDGRANENI 450

Q88JT6-A ALQADADTLVG-----NAQPVG----------QGGNNLTYDNELLDAHYIAGDGRVNENI 400

A5W572-A VLQADADTLVG-----NAQPVG----------QGGNNLTYDNELLDAHYIAGDGRVNENI 400

B0KJL7-A VLQADADSAVG-----NVQPVG----------PGGNNLTYDNELLDAHYIAGDGRVNENI 400

Q88JT6-B VLAPDADNVAG-----NAVPVNP---------QTGANLAYDNELLDAHYIAGDGRVNENI 440

A5W572-B VLAPDADNVAG-----NAVPVNP---------QTGANLAYDNELLDAHYIAGDGRVNENI 440

B0KJL7-B VLAPDADNAVG-----NAVPVNP---------QTGANLAYDNELLDAHYIAGDGRVNENI 440

B5IMQ8 TFNPDGTVDPT---------------------------SYDPALLNEHFIAGDGRVNENQ 341

P05164 CLLTNRSARIP------------------------------------CFLAGDTRSSEMP 247

: ** * .*

Q1YMS2-B ALTSMHTIWARNHNFHVEMLLEAGFEG--------------------------------- 321

Q0G341-B ALTSMHTVWARNHNFHVETLMEAGFEG--------------------------------- 321

A6E280-B ALTSVHTVWARNHNFHVENMLAQGFEG--------------------------------- 310

B7KW13-B ALTSIHTVWARNHNYHVEKLLESGFEG--------------------------------- 318

C7CGY0-B ALTSIHTVWARNHNYHVEKLLESGFEG--------------------------------- 318

A9W3A5-B ALTSIHTVWARNHNYHVEKLLESGFEG--------------------------------- 318

Q13AU2-B ALTSIHTIWARNHNHHVEGLAAAGFQG--------------------------------- 307

Q07SX1-B ALTSIHTIWARNHNHHVEGLEAAGFQG--------------------------------- 308

A3XF15-B GLTSMHTVWARNHNYHVDQLLASGYDAD-------------------------------- 306

B8HDW8 GLTAIHQVFHSEHDRLVGDIKNVLTSDKSSRGTAALTEWRATAGAD-------------- 475

A0JUB7 GLTAIHQVFHSEHNRLVGYMEELLTSQN-----LDLNEWKLPNGQ--------------- 424

B1Y442 ALTAVHSVFHSEHNRLAEQIKELAVSSND---VTFLNQWLDTPVAAVPTT---------- 460

C6MFN2 GLTTVHTVFHAEHNRKVQEIKQLITASGD---TPFMNQWKLP------------------ 448

A6FV45 GLTAVHHVFHTEHNRQAAEMKQTILDSGE---LAFINEWLATPITEA---DLA------- 400

A6FKA5 GLTAVHHVFHTEHNRQAAEMKQTILDSGE---LAFINEWLATPITEA---DLA------- 400

A3XF15-A GLTAVHHVFHSEHNRQTTEMKQTILDSGE---LAFINEWLATPINED---ELS------- 398

A5PER4 ALTAVHHVFHSEHNRQIDSNKIEILKSGD---LAFINEWLDVDITAP---EVAGLAALTD 496

Q11K84 GLTAVHHVFHSEHNRQVEAQKLEILKSKD---LAFINEWLLTDLAS-----VDDIPAG-G 385

B7KW13-A GLTAVHHIFHSEHNRQIDAHKLTILQSGD---LAFINDWLATDIAAL---PGNFAQMT-P 508

A9W3A5-A GLTAVHHIFHSEHNRQIDAHKLTILQSGD---LAFINDWLATDIAAL---PGNFAQMT-A 508

C7CGY0-A GLTAVHHIFHSEHNRQIDAHKLTILQSGD---LAFINDWLATDIAAL---PGNFAQMT-A 508

Q13AU2-A GLTAVHHIFHSEHNRQVDSQKLTILNSGD---IAFINEWLATDIGAL---DPGFGTMT-A 495

Q07SX1-A GLTAVHHIFHSEHNRQVDSQKLTILRSGD---TAFINEWLATDIGGL---PSGFASLS-G 495

Q1YMS2-A ALTAVHHVFHSEHNRLVDATRMEVLKSGD---LAFINEWLATDIATL---EG--IPAD-G 468

Q0G341-A ALTSVHHVFHSEHNRQVEDQKKTILETGD---LEMLNEWLAVDVSEV---PT-------- 360

A6E280-A GLTAVHHVFHSEHNRQVVAQKKTILESGD---IDFINEWLLVDLAAG---DP-------- 411

B7L1G6 ALTAVHQVFHSEHNRVVEMTKQIALDSGD---LAFLNQWLLVDVATI---PIS------- 497

B7KRS2 ALTAVHQVFHSEHNRVVEMTKQIALDSGD---LAFLNKWLLVDVEAM---PTT------- 497

Q88JT6-A GLTAVHHVFHSEHNRLVQQTKDTLLAAGD---LAFLNEWLIDDVTAI---PTT------- 447

A5W572-A GLTAVHHVFHSEHNRLVQQTKDTLLAAGD---LAFLNEWLIDDVTAI---PTA------- 447

B0KJL7-A GLTAVHHVFHSEHNRLVQQTKDTLLAAGD---LAFLNEWLIDDVIAI---PTT------- 447

Q88JT6-B GLTAVHAIFHSEHNRLVAQTMDTVLDSGD---LAFLNEWLLNPVSAL---PVT------- 487

A5W572-B GLTAVHAIFHSEHNRLVAQTMDTVLDSGD---LAFLNEWLLNPVSAL---PVT------- 487

B0KJL7-B GLTAVHAIFHAEHNRLVAQTMDTVLDSHD---LAFLNEWLLNPVTAL---PVT------- 487

B5IMQ8 GLTAFHTIFHNEHDRLVADYKRIALEAAASGDLSFINAWLRPGRQLAS------------ 389

P05164 ELTSMHTLLLREHNRLATELKSLNPRWDG------------------------------- 276

**:.* : :*:

Q1YMS2-B ------------TEEEVFQAAKMINEAEYQRVVFTEFADMLIGGIRGEG----DHGFNDY 365

Q0G341-B ------------TSEEFFQAAKMLNEAEYQRVVFDEFADFLIGGIRGSG----SHGHDEY 365

A6E280-B ------------SDEEIFQAAKMLNESDYQRVVFQEFADKLLGGLRNADGDREDHGWDGY 358

B7KW13-B ------------TPEQVFQAAKMVNEAEYQRVVFDEYLETLIGGLRSDG----THGFEAY 362

C7CGY0-B ------------TPEQVFQAAKMVNEAEYQRVVFDEYLETLIGGLRSDG----THGFEAY 362

A9W3A5-B ------------TPEQVFQAAKMVNEAEYQRVVFDEYLETLIGGLRSDG----THGFEAY 362

Q13AU2-B ------------TAEELFQAAKMINEAEYQRVVFDEYLETLLGGLRSQG----THGFEEY 351

Q07SX1-B ------------TAEELFQAAKMINEAEYQRVVFDEYLETLLGGLRSQG----THGFEEY 352

A3XF15-B ------------TPEELFQAARILNIGEYQQVVFNDFADSLLGGLQGSG----THGHDKY 350

B8HDW8 ----------GWNGERLFQAARFIAEMEYQHLVFEEFARKIQPAVNIFEPFA-----F-S 519

A0JUB7 -----------WNGERLFQAARYVTEMEYQHIVFEDFARKIQPGINGFNVFT-----Q-S 467

B1Y442 ---QAGVDALNWNGERLFQAARFGTEMQYQHFVFEEFARKVQPSIDVFSGY--------- 508

C6MFN2 --------NGNWDGDKLFQAARFATEMQYQHLVFEEFARKVQPNVDVFIGY--------- 491

A6FV45 ---TATVDTVEWDGGRLFQAAKFTTEMQYQHLAFEEFGRTVQPQIAAFGVN--------G 449

A6FKA5 ---TATVDTVEWDGGRLFQAAKFTTEMQYQHLAFEEFGRTVQPQIAAFGVN--------G 449

A3XF15-A ---TAAIDTLTWDGGRLFQAAKFTTEMQYQHLAFEEFGRTVQPQIAAFMVN--------A 447

A5PER4 AQLAAYGESLDWDGERLFQMAKFSTEMQYQHLVFEEFGRRIQPNIDPFVFN--------T 548

Q11K84 AALANYAATLTWDGERLFQSARFATEMQYQHLVFEEFGRKVQPLIDLFIFN--------T 437

B7KW13-A LGQLAYANTLSWDGERLFQAARFATEMQYQHLVFEEFARKIQPLVDPFVFN--------P 560

A9W3A5-A LGQLAYANTLSWDGERLFQAARFATEMQYQHLVFEEFARKIQPLVDPFVFN--------P 560

C7CGY0-A LGQLAYANTLSWDGERLFQAARFATEMQYQHLVFEEFARKIQPLVDPFVFN--------P 560

Q13AU2-A LQQLDYANSLNWDGERLFQGARFATEMQYQHLVFEEFARKIQPAIDPFVFN--------S 547

Q07SX1-A LDQLAYANSLNWDGERLFQAARFATEMQYQHLVFEEFARKIQPAIDPFVFN--------S 547

Q1YMS2-A LPLLNFANTLDWDGERVFQAARFGTEMQYQHLVFEEFARKIQPAIDPFVFN--------S 520

Q0G341-A --DPAVIATLSWDGERLFQAARFATEMQYQHLVFEEFGRKINPNIDPFVFN--------A 410

A6E280-A --IPTDPTALTWDGERLFQAGRFATEMQYQHLVFEEFGRKIHPNIDPFVFN--------A 461

B7L1G6 ---EADRAALVWDGERLFQAGRFTNEMEYQHLVFEEFGRMMQPDIDAFVFE--------P 546

B7KRS2 ---EAERAALVWDGERLFQAGRFTNEMEYQHLVFEEFGRMMQPDIDAFVFE--------P 546

Q88JT6-A ---PADIAALVWDGERLFQAAKFGTEMQYQHLVFEEFARTIQPQIDEFLAP------NGY 498

A5W572-A ---PADIAALVWDGERLFQAAKFGTEMQYQHLVFEEFARTIQPQIDEFLAP------NGY 498

B0KJL7-A ---PAGIAALVWDGERLFQAAKFGTEMQYQHLVFEEFARTIQPQIDEFLAP------NGY 498

Q88JT6-B ---PAEIGALVWNGERLFQAAKFGTEMQYQHLVFEEFARTVQPRVDLFFAPT-----QVY 539

A5W572-B ---PAEIDALVWNGERLFQAAKFGTEMQYQHLVFEEFARTVQPRVDLFFAPT-----QVY 539

B0KJL7-B ---PAEIDALVWNGERLFQAAKFGTEMQYQHLVFEEFARTVQPRVDLFFAPT-----QVY 539

B5IMQ8 GDPVPGVDSDAWDGERLFQAARFMVEMQYQHGVFEEFGRFIDPNIALDSGF--------- 440

P05164 --------------ERLYQEARKIVGAMVQIITYRDYLPLVLGPTAMRKYLP---TYRSY 319

..:* .: * .: :: :

Q1YMS2-B NPNADARISHEFASAVYRVGHSLVGQTMTVIGPDGQ------------------------ 401

Q0G341-B NPDVDARISHEFAAAVYRVGHSLVGQTMTVIGPDGQ------------------------ 401

A6E280-B NPDVDARISHEFAAAAYRFGHSLVGENLQVQGPNGE------------------------ 394

B7KW13-B DPNVDVAISHEFAAAVFRFGHSLIGQTLNVKGADGE------------------------ 398

C7CGY0-B DPSVDVAISHEFAAAVFRFGHSLIGQTLNVKGADGE------------------------ 398

A9W3A5-B DPSVDVAISHEFAAAVFRFGHSLIGQTLNVKGADGE------------------------ 398

Q13AU2-B DPNANAGISHEFAGAVFRFGHSLIGQTMTVLDANGN------------------------ 387

Q07SX1-B DPGADAGISHEFAAAVFRFGHSLIGQTMTVLDADGN------------------------ 388

A3XF15-B DPTTDARISHEFAAAAYRFGHSQIGQSMTLKDVDADG---------------------NP 389

B8HDW8 QTDVNPAINAEFAHSVYRFGHSMLTETISRRNEDSPGPDGVW----------GTQDDVPG 569

A0JUB7 DTGIDPAIQAEFAHATYRFGHSMLTETVDRKLND-------------------------G 502

B1Y442 NSTVDPAIMSEFANVVYRFGHSMLTETVDRIDPLT------------------------G 544

C6MFN2 DSSIDPAIMAEFAHVVYRFGHSMLTEKVERIDAQ-------------------------G 526

A6FV45 SAEIDGAVMAEFAHVVYRFGHSMLTENVHTMDP-------------------------NG 484

A6FKA5 SAEIDCAVMADSP-MWCTASPLDADRDVHTMDP-------------------------NG 483

A3XF15-A SAEVDASIMAEFAHVVYRFGHSMLTENVQTMDP-------------------------NG 482

A5PER4 TTDINPAIFAEFAHVVYRFGHSMLTEDVNRMFLNDAGEPVFY--DELGN--ETPVTDLEG 604

Q11K84 ITDIDPAIFSEFANVVYRFGHSMLTEDIGRMFLNDAGEPVTYSRDDEGNLIETVVTDLTA 497

B7KW13-A VTEIDPSIFAEFANTVYRFGHSMLTENMPRLGP-----------------------DGQA 597

A9W3A5-A VTEIDPSIFAEFANTVYRFGHSMLTENMPRLGP-----------------------DGQA 597

C7CGY0-A VTEIDPSIFAEFANTVYRFGHSMLTENMPRLGP-----------------------DGQA 597

Q13AU2-A VTDINPAIFSEFANTVYRFGHSMLTEAMPRLDA-----------------------NGNP 584

Q07SX1-A VTDINPAIFSEFANTVYRFGHSMLTEGMPRLDG-----------------------AGNS 584

Q1YMS2-A STDIDPSIFSEFANVVYRFGHSMLTETVARTNI-----------------------HDGS 557

Q0G341-A VTDINPAIFAEFANVVYRFGHSMLTDNMPRVFVDET--------------------TGEV 450

A6E280-A VTDINPSIFAEFANVVYRFGHSMLTENMPRVLVNEL--------------------TGEV 501

B7L1G6 SADINPSIAAEFAHVVYRFGHSMLRQDIAVIGMD---------------------ADGKP 585

B7KRS2 SADINPSIAAEFAHVVYRFGHSMLRQDIAVIGMD---------------------ADGKP 585

Q88JT6-A DTSINPAILAEFAHVVYRFGHSMLTETVDRYDPAFN---------------PVSA-DPAN 542

A5W572-A DTSINPAILAEFAHVVYRFGHSMLTETVDRYDPAFN---------------PVSA-DPAN 542

B0KJL7-A DTSINPAILAEFAHVVYRFGHSMLTETVDRFDPSFN---------------PVSG-DPAN 542

Q88JT6-B DVDLDASIVAEFAHTVYRFGHSMLTETVDRFDIDFN---------------VIQDPASAN 584

A5W572-B DVDLDASIVAEFAHTVYRFGHSMLTETVDRFDIDFN---------------VIQDPASAN 584

B0KJL7-B DVDLDASIVAEFAHTVYRFGHSMLTETVDRFDIDFN---------------VIKDPASAN 584

B5IMQ8 NVTLNPAITAEFAHAVYRFGHSMLTESVDRFGIDP--------------------VTGDW 480

P05164 NDSVDPRIANVFT-NAFRYGHTLIQPFMFRLDNRYQP---------------------ME 357

: : . . :

Q1YMS2-B -PRQVELFDAFLNPTSELGAFKPGLP----DGYVPQPGYAQLGAGAILAGVATQSAEE-- 454

Q0G341-B -PREVALFDAFLNPTNEAGAFTGPLP----PGYVPQPGYAQLGVGAILSGTAIQPAEE-- 454

A6E280-B -LIQVPLYDAFLNPSNDPSVFNGPLP----QGYVPAPGYAQYGVAAIIGGTAVQAAEE-- 447

B7KW13-B -TVPVSLFDAFLNPSNDPSVFTAPLP----PGYVPQPGYAQYGVGGIIGGTIEQAAED-- 451

C7CGY0-B -TVPVSLFDAFLNPSNDPSVFTAPLP----PGYVPQPGYAQYGVGGIIGGTIEQAAED-- 451

A9W3A5-B -TVPVSLFDAFLNPSNDPSVFTAPLP----PGYVPQPGYAQYGVGGIIGGTIEQAAED-- 451

Q13AU2-B -PTQVNLFDAFLNPSNDPSAFPNPLP----PGYTPQPGYAQHGVNAIIGGTVSQPAED-- 440

Q07SX1-B -PTQVNLFDAFLNPSNDPSVFPSPLP----PGYTPQPGYAQHGVNAIIGGTVSQPAED-- 441

A3XF15-B FTVEVPLFDIFLNPTNDPDAFTADFGTLEQYGYKPQSGYAQYGVDNILGGLVEQPSEE-- 447

B8HDW8 SQNDLPLLGGFLNP----PAYTD--------GGPAGPLTSEEAAGSIVMGMSDQVGAE-- 615

A0JUB7 TDIGMPLLDAFLNP----PAYYE--------S-TAGTLNPKQAAGAIAMGMTDQVGAE-- 547

B1Y442 ESNPIGLIEAFLNP----VEFGA--------G----TTTTAEAVGNIVTGMTRQTGNH-- 586

C6MFN2 DSNDIGLIQAFLNP----VEFGA--------D----YANNSEAAAAVVRGMTKQVGNE-- 568

A6FV45 VNTSNGLIEAFLNP----VAFD-----------LDQTLTSDQAAGAVARGMSRETGAN-- 527

A6FKA5 VNTSNGLIEAFLNP----VAFD-----------LDQTLTSDQAAGAVARGMSRETGAN-- 526

A3XF15-A VNTSTGLIEAFLNP----VAFD-----------LDQTLTSDQAAGAVARGMSRETGAN-- 525

A5PER4 WGNNTGLIEAFLNP----VDYD-----------LDGNISAEQAAGAIFRGLNREQGNH-- 647

Q11K84 WGDDIGLIEAFLNP----IEFD-----------QNGQISHEQAAGAIFRGMTLVHGNE-- 540

B7KW13-A LDADLGLIDAFLNP----LAFD-----------NDGGLSHDESAAAIMRGMTIERGSE-- 640

A9W3A5-A LDADLGLIDAFLNP----LAFD-----------NDGGLSHDESAAAIMRGMTIERGSE-- 640

C7CGY0-A LDAGLGLIDAFLNP----LAFD-----------NDGGLSHDESAAAIMRGMTIERGSE-- 640

Q13AU2-A MDSELGLVESFLNP----VLFD-----------NDGAISHDAGAAAIVRGMTIERGNE-- 627

Q07SX1-A MDSDLGLVEAFLNP----VLFD-----------NDGAISHDAGAAAIVRGMTIERGNE-- 627

Q1YMS2-A ADN-IGLIQAFLNP----VEFT-----------KNSTVSADEATASIVLGMTSEHGNA-- 599

Q0G341-A STDDMGLIQAFLNP----DVFKR--------DGNDNEISADEAAAAIVRGMTTERGSA-- 496

A6E280-A TTDNMGLIAAFLNP----VAYD-----------NDGAMSADAAAAAVILGMTTEQGSQ-- 544

B7L1G6 VQNDISLFDGFLNP----IMYD-------------SLGDAEAASGAIIRGMSRQTGSE-- 626

B7KRS2 VQNDISLFDGFLNP----VMYD-------------SLGDAEAASGAIIRGMSRQTGSE-- 626

Q88JT6-A PDQQLGLIAAFLNP----LAFA------------GSGATADEAAGAIIRGVTRQVGNE-- 584

A5W572-A PDQQLGLIAAFLNP----LAFA------------GSGATADEAAGAIIRGVTRQVGNE-- 584

B0KJL7-A PDQQLGLIAAFLNP----LAFA------------GSGATADEAAGAIIRGVTRQLGNE-- 584

Q88JT6-B PDQQLGLIAAFLNP----LAYA------------ASGVTPEDATSAIVRGVTRQAGNE-- 626

A5W572-B PDQQLGLIAAFLNP----LAYA------------ASGVTPEDATSAIVRGVTRQAGNE-- 626

B0KJL7-B PDQQLGLIAAFLNP----LAYA------------ASGVTPEDATSAIVRGVTRQGGNE-- 626

B5IMQ8 SNNSESLIAAFLNP----VGFLQSLD----AVGTTQDLTPEEAAAEVLRGMTRQQGND-- 530

P05164 PNPRVPLSRVFFAS---------------------WRVVLEGGIDPILRGLMATPAKLNR 396

* *: . . : * .

Q1YMS2-B ----VDFNIVDAIRNDLVRINADLFAFNVARGWDVGLGTLNQVRADLKASGD**PYIQEAVG** 510

Q0G341-B ----VDFNIVDAIR-------------------TTSFGSM--------PTCS**PSTSLAAG** 483

A6E280-B ----VDLKIVEAIRSDLVRINADLFSFNVARGWDVGLGTMNQVRTQLAASTD**PYVSQAVD** 503

B7KW13-B ----VDFNIVDAVRNDLVRIRADLFAFNVARGWDVGLGTLNQVRADLAASTN**PYIRDAVG** 507

C7CGY0-B ----VDFNIVDAVRNDLVRIRADLFAFNVARGWDVGLGTLNQVRADLAASTN**PYIRDAVG** 507

A9W3A5-B ----VDFNIVDAVRNDLVRIRADLFAFNVARGWDVGLGTLNQVRADLAASTN**PYIRDAVG** 507

Q13AU2-B ----VDFNIVDAVRNDLVRINADLFAFNVARGWDLGLGTLNQVRRDLAASTN**PYVAESVG** 496

Q07SX1-B ----VDFNIVDAVRNDLVRINADLFAFNVARGWDVGLGTLNQVRQDLAASTN**PYVSEAVG** 497

A3XF15-B ----VDLQVVDAVRNDLVRVSADLFAFNVARGRDVGLGTLNQVKADLAASDN**RYISEAID** 503

B8HDW8 ----LDEFVTDTLRSKLLGLPMDLAAINLARGRSEGIPALNVFRRQLHGATN-------- 663

A0JUB7 ----LDEFVTDTLRNNVLGLPLDLASLNLARGRDTGIPSLNNFRTQLYASTG-------- 595

B1Y442 ----IDEFITEALRNNLVGLPLDLAALNIARGRDTGIPGLNAARAMFFEDTG-------- 634

C6MFN2 ----VDEFVTEALRNNLLGLPLDLPTINITRGRDTGMPGLNDARRMFFDDVG-------- 616

A6FV45 ----IDEFITSALRDNLVGLPLDLAALNIARGRETGVPSLNAAREQFYSATG-------- 575

A6FKA5 ----IDEFITSALRDNLVGLPLDLAALNIARGRETGVPSLNAAREQFYSATG-------- 574

A3XF15-A ----IDEFITSALRDNLVGLPLDLAALNITRGRDTGVPSLNAAREQFYAATG-------- 573

A5PER4 ----IDEFVTDALRNNLLGLPLDLAAINIARGRDTGMPTLNEARAELYDATG-------- 695

Q11K84 ----IDEFVVDALRNNLLGLPLDLAAINIARGRDTGMPTLNQARTQLYEASN-------- 588

B7KW13-A ----IDEFVVGALRNNLLGLPLDLAAINIARGRDTGTPTLNEARAQLYAATG-------- 688

A9W3A5-A ----IDEFVVGALRNNLLGLPLDLAAINIARGRDTGTPTLNEARAQLYAATG-------- 688

C7CGY0-A ----IDEFVVGALRNNLLGLPLDLAAINIARGRDTGTPTLNEARAQLYAATG-------- 688

Q13AU2-A ----IDEFVVDALRNNLLGLPLDLAAINIARGRDTGMPSLNETRTQLYAASG-------- 675

Q07SX1-A ----IDEFVVDALRNNLLGLPLDLAAINIARGRDTGMPSLNDARTQLYAASG-------- 675

Q1YMS2-A ----IDEFITSALRNNLLGLPLDLAAINIARGRDTGMPTLNETREQLYQATG-------- 647

Q0G341-A ----IDEYVVSSLRSNLLGLPLDLPALNIARGRETGMPTFNDARAELYGQTN-------- 544

A6E280-A ----IDEFIVPALRSNLLGLPLDLAAINIARGRDTGIPSFNDARAELFQQTN-------- 592

B7L1G6 ----IDEFVTDVLRNQLLGIPLDLATINLARGRDVGTPTLNVARERFFEETG-------- 674

B7KRS2 ----IDEFVTDVLRNQLLGIPLDLATINLARGRDVGTPTLNVARERFFEETG-------- 674

Q88JT6-A ----IDEFVTEALRNNLLGLPLDLPALNIARGRDTGIPSLNEARREFYAATG-------- 632

A5W572-A ----IDEFVTEALRNNLLGLPLDLPALNIARGRDTGIPSLNEARREFYAATG-------- 632

B0KJL7-A ----IDEFVTEALRNNLLGLPLDLPALNIARGRDTGIPSLNEARREFYGATG-------- 632

Q88JT6-B ----IDEFVTEALRNNLLGLPLDLPAINIARGRDVGIPSLNAVRREIYGQTG-------- 674

A5W572-B ----IDEFVTEALRNNLLGLPLDLPAINIARGRDVGIPSLNAVRREIYGQTG-------- 674

B0KJL7-B ----IDEFVTEALRNNLLGLPLDLPAINIARGRDVGIPSLNAVRRDVYGQTG-------- 674

B5IMQ8 ----IDEFVTPALRNNLVGLPLDLATINIARGNELGLPKLNAARAEFFSLTGG------- 579

P05164 QNQIAVDEIRERLFEQVMRIGLDLPALNMQRSRDHGLPGYNAWRRFCGLPQP-------- 448

: : .

Q1YMS2-B **FA**GN-LDPYASWADFQARNGLSDTIMDQMKVAYPDLILSTP----EEIAAFIAVNPDIEL 565

Q0G341-B **TSG**--------WAR---------------------------------------------- 489

A6E280-B **MAG**D-LSPYSSWADFQARNDVSDEDMARLMEAYPDLVLETP----AQIAAFVAVNPDVVL 558

B7KW13-B **FAG**GDLSPYASWEDFQARNGLSDAVIAQFRQAYPDLVLAA-----ADIAAFRAINGDIAI 562

C7CGY0-B **FAG**GDLSPYASWEDFQARNGLSDAVIAQFRQAYPDLVLAA-----ADIAAFRAINGDIAI 562

A9W3A5-B **FA**GGDLSPYASWEDFQARNSLSDAVIAQFRQAYPDLVLAA-----ADIAAFQAINGDIAI 562

Q13AU2-B **FA**GSNLTPYSSWEDFQQRNDLNNAVIAQFKQAYPDLTLAA-----ADIAAFREVNPDIAI 551

Q07SX1-B FAGGDLSPYTSWEDFQQRNGLSQAVIEQFKQAYPDLQLAA-----ADVAAFQAINPDIDI 552

A3XF15-B **LS**DMSMTPYTDWEDFQARNGLSDEMIAKFQTAYPALVLTVDT---EQYDAFVEANPDIAL 560

B8HDW8 --DSQLKPYANWIDFGENIKHPASLINFIAAYGTHPSIVSA----TTLDAKRKAARLIVS 717

A0JUB7 --ESSLKPYTSWVDFGQNLKHPDSVVNFMAAYGTHETITAA----ASITDKRAAAQRLFD 649

B1Y442 --LSSLQPYDSWSDFGLGIKNPESLVNFIAAYGTDASITGA----TSLADKRAAAQAILD 688

C6MFN2 --HPSLAPYESWNDFKLDLKNPESLTNFIAAYGNHPSINSV----TTMEDKRAAADLLVK 670

A6FV45 --SEFLKPYEGWSEFAANLKNPASIINFIASYGTHETIVNA----TTVVEKRAAATDLVL 629

A6FKA5 --SEFLKPYEGWSEFAANLKNPASIINFIASYGTHETIVNA----TTVVEKRAAATDLVL 628

A3XF15-A --SEFLKPYEGWSDYAANLKNPASIINFIAAYGTHDTIANA----TTVVQKRAAATDLVL 627

A5PER4 --STFLTPYGSWAEFAEGLKNPLSVINFIAAYGTHQTIIDAG---DNVDARRAAATDLVL 750

Q11K84 --STFVKPYANWTDFAANLKTPASIINFIAAYGTHDLILAAE---DDVAARRAAATLIVL 643

B7KW13-A --STFLTPYTSWVEMAANLKNPLSVVNFIAAYGTHGTVVAA----TTLAAKRDAAMALVF 742

A9W3A5-A --STFLTPYTSWVEMAANLKNPLSVVNFIAAYGTHGTVVAA----TTLAAKRDAAMALVF 742

C7CGY0-A --STFLTPYTSWVEMAANLKNPLSVVNFIAAYGTHGTVVAA----TTLAAKRDAAMALVF 742

Q13AU2-A --STFLKPYDHWVDLATNLKNPASIVNFVAAYGTHATIVGA----TTLEAKRMAAMELVF 729

Q07SX1-A --STFLKPYDHWVDFAANLKNPASIVNFVAAYGTHSTIAGA----TTLEAKRLAAMELVF 729

Q1YMS2-A --SSFLKPYDSWVDFAANLKNPMSVVNFIAAYGTHETIVAAG---NNLQERRNAAMALVF 702

Q0G341-A --SVWLKPYESWADLAQNLKTPMTVVNLIAAYGLHETVTGA----TTLADKRAAAFDLVF 598

A6E280-A --SVWLKPYENWVELAANLKTPMTIVNLLAAYGTHSTILAA----NTLEEKRDAAFDLVF 646

B7L1G6 --DTLLKPYESWADFALSLKNPASIINFIAAYGNHASVLNTADAPRTVADLRAAATKLVL 732

B7KRS2 --DTLLKPYESWADFALSLKNPASIINFIAAYGNHASVLNTADAPRTVADLRAAATKLVL 732

Q88JT6-A --DSQLKPYMSWVDFADHLKHPASLINFIAAYGTHSSITGA----TTEAAKRAAAVALVL 686

A5W572-A --DSQLKPYISWVDFADHLKHPASLINFIAAYGTHSSITGA----TTEAAKRAAAVALVL 686

B0KJL7-A --DSQLKAYISWADFADHLKHPASLINFIAAYGTHSSITGA----TTEAAKRAAAVALVL 686

Q88JT6-B --DTQLKPYTSWVDLVQHLKHPESLINFIAAYGTHSTITNA----TTLLEKRAAAMALVF 728

A5W572-B --DTQLKPYSSWVDLVQHLKHPESLINFIAAYGTHSTITAA----TTLLEKRAAAMALVF 728

B0KJL7-B --DTQLKPYTSWVDLVQHLKHPESLINFIAAYGTHSSITGA----TTLLEKRAAAMALVF 728

B5IMQ8 --NPSLTPYSNWNDFRSHLKHDASIYNFVAAYGTHPTITSA----TTVEAKRAAAMDLVD 633

P05164 ---------ETVGQLGTVLRNLKLARKLMEQYGTP------------------------- 474

Q1YMS2-B TDGANG----------------------------------------TKIVKGIDRVDLWV 585

Q0G341-B ------------------------------------------------------------

A6E280-B EDGANG----------------------------------------AKIVKGIDRVDLWT 578

B7KW13-B AMQADG----------------------------------------TGVVKGIDRLDLWV 582

C7CGY0-B AMQADG----------------------------------------TGVVKGIDRLDLWV 582

A9W3A5-B AMQADG----------------------------------------TGVVKGIDRLDLWV 582

Q13AU2-B AMQNDG----------------------------------------TGIVSGIDRLDLWV 571

Q07SX1-B AMNDDG----------------------------------------TGIVKGIDRVDLWV 572

A3XF15-B IDNGDG----------------------------------------TMTVKGIDRVDLWV 580

B8HDW8 PDAL----AGEVAPDDAVAFMNSTDAWANNGT---------------ASTTGLDDIDLWM 758

A0JUB7 MDT-----ADPATPADSYDFVNSAGAWASQ-------------------PSGLNNVDLWV 685

B1Y442 AAE--------LGDAAALAFLEGP----AAT-------------------TGVNDIDFWI 717

C6MFN2 GGP--------GAPADRANFMNSTGAYTTAN-------------------SGLDSVDFWI 703

A6FV45 GGDN--------APADRLDFVNGTGAWA-------------------DAETGINDVEFWI 662

A6FKA5 GGDN--------APADRLDFVNGTGAWA-------------------DAETGINDVEFWI 661

A3XF15-A GGDT--------APADRLDFVNGTGAWA-------------------TIETGINAVEYWI 660

A5PER4 GGG-------AVSEADRLAFLNGP-----------------------AAETGVNDIDMWI 780

Q11K84 GG--------QDAPADRLEFLNGTGAWS-------------------GVETGLNLVDLWI 676

B7KW13-A GGDG--------APTDRLDYLNSRGSWA-------------------GRETGFGAVDLWI 775

A9W3A5-A GGEG--------APTDRLDYLNSRGSWA-------------------GRETGFGAVDLWI 775

C7CGY0-A GGEG--------APTDRLDYLNSRGSWA-------------------GRETGFGAVDLWI 775

Q13AU2-A GVDQNG---DATVAADRTAFLTGTGAWA-------------------GVETGLNRIDLWI 767

Q07SX1-A GVDQDG---DATVAADRTAFLTGTGAWA-------------------GVETGLNRIDLWI 767

Q1YMS2-A NTEG--------APADRLAFLNSTGGET-------------------AESVGLNDIDLWV 735

Q0G341-A GSES-------LNDTDRLDFMLSRGEWN-------------------AANNGLNEIDLWV 632

A6E280-A GGGG-------VSDADRFDFLLGRNGWT-------------------SDTNGLNTIDLWV 680

B7L1G6 GDSALTGDAKAAFDADRLDFLNHTGAYAGD-----------------GSLGGLNTVDFWM 775

B7KRS2 GDSALTGDAKAAFDADRLDFLNHTGAYAGD-----------------GSLGGLNTVDFWM 775

Q88JT6-A GGDG--------APLDRLDFLNGTGAYANVTLAGADGIAGTADDIAGVTVTGVDAIDFWV 738

A5W572-A GGDG--------APLDRLDFLNGTGAYANVTLAGADGIAGTADDIAGVTVTGVDAIDFWV 738

B0KJL7-A GGAG--------APADRLDFLNSTGAWANVTLAGKDGIAGTADDIAGVTVSGVDAIDFWV 738

Q88JT6-B GGDG--------APADRMDFLNSSGAWANVTLPGKDGVLGTADDLKAVTVTGVDAIDLWI 780

A5W572-B GGDG--------APADRMDFLNSSGAWANVTLPGKDGVLGTADDLKAVTVTGVDAIDLWI 780

B0KJL7-B GGEG--------APADRLDFLNSSGAWANVTLPGKDGVLGTADDLKAVTITGVDAIDLWI 780

B5IMQ8 YVHP-----------DGPAFMNAQGGFAG-------------------QRAGLDNVHLWI 663

P05164 -----------------------------------------------------NNIDIWM 481

Q1YMS2-B GGLAEKHVLGG-MVGQTFWVVLHEQFDRLQEGDRFYYLERFDNFDFYDNFIDGQEFSDII 644

Q0G341-B ------------------------------------------------------------

A6E280-B GGLAEKHVNGG-MVGQTFWVVLHEQLDRLQEGDRFYYIDRFDNFDFYQEFGEDTTFASIV 637

B7KW13-B GGLAEKHINNG-VVGQTFWVVLHEQFDRLQDGDRFYYLERFDNFDFYENLVDGQGFSDIV 641

C7CGY0-B GGLAEKHINNG-VVGQTFWVVLHEQFDRLQDGDRFYYLERFDNFDFYENVVDGQGFSDIV 641

A9W3A5-B GGLAEKHINNG-VVGQTFWVVLHEQFDRLQDGDRFYYLERFDNFDFYENVIDGQGFSDIV 641

Q13AU2-B GGLAEKHINGG-LVGETFWVVLSEQFERLQDGDRFYYISRFDNFDFYENFIDGQQFADIV 630

Q07SX1-B GGLAEQHINGG-LVGQTFWVVLSEQFERLQDGDRFYYISRFDNFDFYENFIDGQEFADII 631

A3XF15-B GGLAEQHIQDG-VVGHTFWVLIHEQLDRLQEGDRFYYVDQIGDLPVYNNFISNLTFGDIV 639

B8HDW8 GGLAERTNMFGGLLGSTFNYVFESQMTDLQNGDRLYYLARTPGMNLMAQLEGN-SFAELI 817

A0JUB7 GGLAERQNLFGGLLGSTFNYIFERQMTDLQDGDRLYYLSRTSGLNLRTQLEGN-SLAELI 744

B1Y442 GGLAEVTQPFGGMLGTTFDYVFSTTLLQLQNNDRLYYLARTAGLNVLTQLEEG-SLAELV 776

C6MFN2 GGMAEKQAPFGGLLGSTFNYVFETQMENLQNGDRFYYLARTAGMDFLTQLEEN-SFAEMI 762

A6FV45 GGLAEDIMPFGGMLGSSFGFVFQQQMEALQNGDRFYYLARTAGMNMIAELENN-SFASMI 721

A6FKA5 GGLAEDIMPFGGMLGSSFGFVFQQQMEALQNGDRFYYLARTAGMNMIAELENN-SFASMI 720

A3XF15-A GGLAEAIMPFGGMLGSSFGFAFQQQMEALQNGDRFYYLSRTNGMDMLGGLENN-SFASMI 719

A5PER4 GGLAEAPMAFGGFLGSTFNAVFEAQMEALQDNDRFYYLSRTQGLNFLNELENN-AFSKMV 839

Q11K84 GGLAEKKMPFGGMLGSTFNAVFEAQLEMLQDLDRFYYLTRTQGLNLLNELENN-AFSKLV 735

B7KW13-A GGLAEKQMPFGGMLGSTFNAIFEAQMENLQDADRFYYLSRVQGQNFLNELEQN-SFSKIM 834

A9W3A5-A GGLAEKQMPFGGMLGSTFNAIFEAQMENLQDADRFYYLSRVQGQNFLNELEQN-SFSKIM 834

C7CGY0-A GGLAEKQMPFGGMLGSTFNAIFEAQMENLQDADRFYYLSRVQGQNFLNELEQN-SFSKIM 834

Q13AU2-A GGLAEKKMPFGGMLGSTFNAIFELQLENLQDGDRFYYLTRTQGQNFLNMLEQN-SFAKLI 826

Q07SX1-A GGLAEKKMPFGGMLGSTFNAIFELQLENLQDGDRFYYLTRTQGQNFLNMLEQN-SFAKMI 826

Q1YMS2-A GGLAEQILLFGGMLGSTFAAIFEAQLEALQDGDRFYYLSRTQGLNLLNELENN-AFSKLI 794

Q0G341-A GGLAERIMPFGGMLGSTFSAIFEAQMEALQFGDRFYYLTRTQGQNLLNELEEN-AFAKII 691

A6E280-A GGLAERIMPFGGMLGSTFTAIFEAQMEALQDGDRFYYLTRTQGQNFLNELEEN-SFSKML 739

B7L1G6 GGLAEKKMAFGGMLGSTFSFVFQMTMENLQDADRFYYLSRTQGLNLLNELENN-TFAELV 834

B7KRS2 GGLAEKKMAFGGMLGSTFSFVFQMTMENLQDADRFYYLSRTQGLNLLNELENN-TFAELV 834

Q88JT6-A GGLAEKKMPFGGMLGSSFNFVFETQLEALQNGDRFYYLSRTAGMNFGTELENN-SFAKLI 797

A5W572-A GGLAEKKMPFGGMLGSSFNFVFETQLEALQNGDRFYYLSRTAGMNFGTELENN-SFAKLI 797

B0KJL7-A GGLAEKKMPFGGMLGSSFNFVFETQLEALQNGDRFYYLSRTAGMNFGTELENN-SFAKLI 797

Q88JT6-B GGLAEAKAPFGGMLGSTFNFVFENQMEKLQDGDRFYYLERTAGLSMNAELESN-SFAKLI 839

A5W572-B GGLAEAKAPFGGMLGSTFNFVFENQMEKLQDGDRFYYLERTAGLSMNAELESN-SFAKLI 839

B0KJL7-B GGLAEEKTPFGGMLGSTFNFVFENQMEKLQDGDRFYYLERTSGLSMNAELESN-SFAKLI 839

B5IMQ8 GGLAEAPEAGVTMLGTTFAFVFEQQLQALQNADRLYYLRRLNG-NLLTQIENN-TFAAMA 721

P05164 GGVSEP-LKRKGRVGPLLACIIGTQFRKLRDGDRFWWENEGVFSMQQRQALAQISLPRII 540

Q1YMS2-B ARNTGLTGLPE-----------------EIFRAN---------------------DENDD 666

Q0G341-B ------------------------------------------------------------

A6E280-B ARNTSLTDIDN-----------------NLFDANGIDDDDDNATEDDNATE----DDNAT 676

B7KW13-B ARNTGLTVLPE-----------------HIFELSDEDGPGTEPGD----------DDDDD 674

C7CGY0-B ARNTGLTVLPE-----------------HIFELSDEDGPGTEPGD----------DDDDG 674

A9W3A5-B ARNTGLTVLPE-----------------HIFELSDEDGPGTEPGD----------DDDDG 674

Q13AU2-B TRNTGMTGIPE-----------------HMFQTDPID--QNENEG----------EGEGE 661

Q07SX1-B ARNTGMTGLPE-----------------HMFRTDPIDDENNQNPD----------DNEGD 664

A3XF15-B TRNTGMTDLPQ-----------------DVFSYTGDEIVEDNGTADQQTQQPPVTDDAGN 682

B8HDW8 MRNT-NAKALK----------------ADAFATADC-KFELKNLAGTSEGFAASGNTVAD 859

A0JUB7 MRNT-DAEALK----------------ADVFGVADC-EFELGRIT------AGTGNSVAD 780

B1Y442 MRNS-TAKHLP----------------GDIFSTPTY-IFEVGTMN-------PTGRPLSD 811

C6MFN2 MRNLPDVKHLP----------------FDVFSTPTY-IFEASAQT-------VSG-TIID 797

A6FV45 VRNTDIKDGG-------------AHIPANIFSSMDV-ILEVDQAVQ-------SMPDPVS 760

A6FKA5 VRNTDIKDGG-------------AHIPANIFSSMDV-ILEVDQAVQ-------SMPDPVS 759

A3XF15-A MRNTDIADGG-------------AHIPANIFSSMEY-ILEVDQSVQ-------AMADPVS 758

A5PER4 MANTDMTDPGPDGIRGTEDDILNYHSHVDAFTMADF-LLHVDPTKQ-------IGPDPQH 891

Q11K84 MANTDMTLPGPDGVRGTEDDIVNYHVGVDSFAKHDL-VLHVDPDKQ-------IGDDPTH 787

B7KW13-A LANSSLSLPGPDGIRGTADDIVPRHIGVDAFADYDF-ELEVNAANQLDQNGAAPGRDPTG 893

A9W3A5-A LANSSLSLPGPDGIRGTADDIVPRHIGVDAFADYDF-ELEVNAANQLDQNGAAPGRDPTG 893

C7CGY0-A LANSSLSLPGPDGIRGTADDIVPRHIGVDAFADYDF-ELEVNAANQLDQNGAAPGRDPTG 893

Q13AU2-A MANTDLAQPGPDGIRGTADDIVPRHIGVDSFANYDY-VLEVDESNQADYNGAAPGKDPQG 885

Q07SX1-A MANTDLAQPGPDGIRGTADDIVPRHIGVDSFADYDY-VLEVDEANQEDYNGAAAGKDPHG 885

Q1YMS2-A IANTDLSDPGPDGIRGTGDDVIARHIGVDAFGQYDY-VLEVNKSNQ-------LIEDPTG 846

Q0G341-A MANTNLTLPGPDGIKGTEDDVTPHHIGIDVFADYDF-VLEVNKANQ-------LIEDPEG 743

A6E280-A LANTSLADPGADGIRGTEDDVVRHHIGVDSFARYDF-VLEVNQANQ-------LIDDPVG 791

B7L1G6 MRNTDLGDAHSTALPG------------NLFSAFQMPTLELDISKQ-------LGADPVS 875

B7KRS2 MRNTDLGDAHSTALPG------------NLFSAFQMPTLELDISKQ-------LGADPVS 875

Q88JT6-A MANSDVTHLSN-----------------TVFLTPTF-TLEVNQANQFTGLGADGKADPTG 839

A5W572-A MANSDVTHLSN-----------------TVFLTPTF-TLEVNQANQFTGLGADGKADPTG 839

B0KJL7-A MLNSDVTHLSN-----------------TVFLTPTF-TLEVNQANQFTGLGADGKADPTG 839

Q88JT6-B MANSSATHLPG-----------------LVFSDPGF-YLELDQTKQYN--EGLGSADPLG 879

A5W572-B MANSSATHLPG-----------------LVFSDPGF-YLELDQTKQYN--EGLGSADPLG 879

B0KJL7-B MANTSAAHLPG-----------------LVFSDPGL-YLELDQSKQYN--DGLGHADPLG 879

B5IMQ8 ERALGGAITSLPG---------------HIFSLPTY-TLEVDAATQHTNLGLDGKADPVG 765

P05164 CDNTGITTVSKN----------------NIFMSNSY------------------------ 560

Q1YMS2-B TADNDDGVGDDTSDED---GD------SDTVGEDNNDDTVVDDDDALAP----------- 706

Q0G341-B ------------------------------------------------------------

A6E280-B EDDDDTAGGDDDNATD---GD------DDTAGGDEDNATDGDDDTADGDEDTASDDQDDT 727

B7KW13-B DGVTDPVGGDPDEDED--GPT------DPVGGGGDAGGDEDEDDGVTDPVGGGDDPGDDE 726

C7CGY0-B D--TDPVGGDPDEDED--GPT------DPVGGGGNAGGDEDEDDGVTDPVGGGDDPGDDE 724

A9W3A5-B D--TDPVGGDPDEDEDEDGPT------DPVGGGGDAGGDED--DGVTDPVGGGDDPGDDE 724

Q13AU2-B EDGTPVGNGDPPTDDD-----------------DDDDDDD--DDDDGDGTAGGD---DDD 699

Q07SX1-B DDGTPVGNGDDETDDE-----------------DDDDTASGGDDEDDDETAGGD---DDD 704

A3XF15-B ANQADTGQDDTGQDDN---GQ------GQTAQGDDTQGEMTVDNSTQTAQGGASADQEPV 733

B8HDW8 D--AGT-ECSETALLLR--------------MPDGTIKYRASNSVDPV--GIN------- 893

A0JUB7 D--PAS-ACDESALLMR--------------MSDGTIRYRVSNTVDRP--GLN------- 814

B1Y442 D--PSTPDVNESLLLTR--------------MADGTIRYAGAEHILMG--GTDGADR-LR 852

C6MFN2 D--PDTTTYDERDLLIRDTS-----------LGPDTIRYTGEDHVVMG--GTESNDR-LR 841

A6FV45 TDVDPFLAAMGTTMVERATATVDAPLIDGVREYDNFLKFNGGEHAVLG--GTDQRDI-LN 817

A6FKA5 TDVDPFLAAMGTTMVERATATVDAPLIDGVREYDNFLKFNGGEHAVLG--GTDQRDI-LN 816

A3XF15-A TELDPFLAAMGTTLVERETGTGDAPLVDGAREYDNLLKFNGGEHVVLG--GTDQRDI-LV 815

A5PER4 D--DEVLNSIGQTLVQRDDL-------ATAEVEVNYIKFIGADHTTIN--GTAGDDT-II 939

Q11K84 E--DPALNAVGLTKVQRDDL-------TTTGPDENYIRFLGGEHVVIG--GTEGDDT-II 835

B7KW13-A N--DPVLEAMGLGKVVRDDP-------GTAA-DEGASGFHASVNALVRRFGADGSPTGAL 943

A9W3A5-A N--DPVLEAMGLGKVVRDDP-------GTAA-DEGASGFHASVNALVRRYGADGSPTGAL 943

C7CGY0-A N--DPVLEAMGLGKVVRDDP-------GTAA-DEGASGFNASVNALVRRYGADGSPTGAL 943

Q13AU2-A A--DPFLEALGLGKVIRNDP-------GTAGPDENYIRFSGGEHIVVG--GTDGDDT-II 933

Q07SX1-A A--DPFMEALGLGKVIRDDP-------GTAGPDANYIRFSGGEHIVVG--GTSGNDT-II 933

Q1YMS2-A V--DPVLEALGLGKVIRDDP-------RTPQDESAVSGYVASINALVKQYDASGTPTGIL 897

Q0G341-A N--DPILEALGRKKVLRDDL-------TTSEVETNYIKFTGGEHIVVG--GTNDDDT-II 791

A6E280-A N--DPVLEGLGMGKVVRDDP-------TTSEVETNYIRVTGGEHLAVG--GTNGNDT-II 839

B7L1G6 D--DPFLG--GFSKLIERVD-------ANGDGIAESIRVNSNEHFTIG--GTEGNDI-IV 921

B7KRS2 D--DPFLG--GFSKLIERVD-------ANGDGIAESIRVNSNEHFTIG--GTEGNDI-IV 921

Q88JT6-A G--IEINGVEIVPLVIRDNPD-------TVGPDSNFLHYTGEDHVVLG--GTAGNDI-II 887

A5W572-A G--IEINGVEIVPLVIRDNPD-------TVGPDGNFLHYTGEDHVVLG--GTAGNDI-II 887

B0KJL7-A G--IEINGVEIVPLVIRDNPD-------TVGPDSNYLHYTGEDHVVLG--GTSGNDI-II 887

Q88JT6-B -----ENGEQVV---FRDSPL-------TAGPDTHYIRYAGAEHIVLG--GTNGDDI-LV 921

A5W572-B -----ENGEQVV---FRDSPL-------TAGPDTHYIRYAGAEHIVLG--GTNGDDI-LV 921

B0KJL7-B -----ENGEQVV---FRDSPL-------TAGPDSNYIRYAGAEHIVLG--GTNGDDI-LV 921

B5IMQ8 G--AVSSNLQEVYRTSQGDPN-HSTISQIGFNVANVLRYNGLGHVVLG--GGAEADL-LQ 819

P05164 --------------------------------PRDFVNCSTLPALNLASWREAS------ 582

Q1YMS2-B ----------------------VPPVVSGLAVTGTAAADVMMGGAE----ADVLSGGDGD 740

Q0G341-B ------------------------------------------------------------

A6E280-B AGDNDTEGDEDDDEPEDNDSNVTPPLAASGNVIGTAAADALFGGAE----GDNILALAGR 783

B7KW13-B DDGQGDGDGTTDPVGGGDGDEDGPGTGPGTNPPVNHAPGVIAGGAN----GDVL------ 776

C7CGY0-B DDGQGDGDGTTDPVGGSDGDEDGPGTGPGTTPPVNHAPGVIAGGAN----GDVLNGTAGA 780

A9W3A5-B DDGQGDGDGTTDPVGGGDGDEDGPGTGPGTNPPVNHVPGVIAGGAN----GDVL------ 774

Q13AU2-B D--------DDTPPANGEGDGTTPP-VGGVIRTGTPQPDVLVGGAG----DDNIVAFADD 746

Q07SX1-B DEITGGDDDDDDETVGGDDDVTPPPSTPGVIRTGTPQPDVLVGSAG----DDNIVAFADA 760

A3XF15-B SNDDDTIDGGDSSDADTETPASDPSTEQSPN--GSDEGLVKSGTDL----GDALVGAAGD 787

B8HDW8 ---GQAVYNGT------DRADRVHGGVDNDTFWGGKGNDVIEGGD----GADTVLGGEDN 940

A0JUB7 ---AQSTFNGT------ALGDRIWGGIDNDTFWGNDGQDIIEGND----GADTVLGGDGN 861

B1Y442 AGAGDDTLHGD------AGNDRLEGGAGNDFFFAGEGDDILTDSF----GDDNMKGQGGN 902

C6MFN2 AGAGDDTLWGD------GGNDRLEGGAGNDSINGGDGNDRITDAS----GDDNIKGGDGH 891

A6FV45 GGLGDDALWGG------AGDDLLIGDAGVNTLRGGAGNDILKDGD----D---------- 857

A6FKA5 GGLGDDALWGG------AGDDLLIGDAGVNTLRGGAGNDILKDGD----DVSFLHGEDGD 866

A3XF15-A GGLGDDALWGG------AGDDLLIGDSGVNTFRGGDGNDIIKDGD----DIS-------- 857

A5PER4 AGGGDDGIWGG------AGNDRIEGGHGVDLIIAGAGNDIVTDSG---DSDDFIKGDEGD 990

Q11K84 SDFGDDALWGG------GGNDRIEGGAGVDLIIGGSGDDIITDSG---DTGDFIKGEDGD 886

B7KW13-A VDGSEDGVGGAGSPVTWADLKANAAKLGIALTQADMLDAPVLRIG---A----------- 989

A9W3A5-A VDGSEDGGGDAGSPVTWADLKANAAKLGIALTQADMLDAPVLRI---------------- 987

C7CGY0-A VDGSEDGVGGAGSPVTWADLEANAAKLGIALTQADMLDAPVLRIG---ADGRLAFAPGSS 1000

Q13AU2-A TDFGDDGIWGD------AGDDRIESGAGVDLVNGGAGNDIITDSG---DTG--------- 975

Q07SX1-A TDFGDDGIWGD------DGDDRIESGAGVDLVNGGAGNDIITDSG---DTGDFIKGDEGD 984

Q1YMS2-A IDGSENVIPGIT----VGDLRENAEHLGIILTDEDLANAPVLKLN---PDGTLRFN---- 946

Q0G341-A TDDGDDAIWGD------AGDDYIESGFGVDLVNGGYGNDIILDAG---DEGDFLKGDEGD 842

A6E280-A TSDGDDGIWGD------DGDDFIESGFGVDLVNGGGGNDIILDSG---DEGDFLKGEGGD 890

B7L1G6 SGGGDDAIWGK------AGDDRIEAGYGVDKVFGGAGDDIITNAGTDIGEADFLHGNEGN 975

B7KRS2 SGGGDDAIWGK------AGDDRIEAGYGVDKVFGGAGDDIITNAGTDIGEADFLHGNEGN 975

Q88JT6-A SSEGDDTLYGD------AGDDLLEGGAGNDAVLGGAGDDIITDSF----GDNRLEG---- 933

A5W572-A SSEGDDTLYGD------AGDDLLEGGAGNDAVLGGAGDDIITDSF----GDNRLEG---- 933

B0KJL7-A SGDGDDTVYGD------AGDDVLEGGAGNDAVLGGAGDDIITDSF----GDNRLEGNAGN 937

Q88JT6-B SSEGDDTVWGD------AGNDRIEGGDGNDQLRGGAGDDIISDMG----GDDNIQGGDGN 971

A5W572-B SSEGDDTVWGD------AGNDRIEGGDGNDQLRGGAGDDIISDMG----GDDNIQGGDGN 971

B0KJL7-B SSEGDDTVWGD------AGNDRIEGGDGNDQLRGGAGDDIISDMG----GDDNIQGGDGN 971

B5IMQ8 AGSGNDTVYGR------GGNDVIFSGQGDDQVWGGEGNDFISDNF----GLNFLRGEAGH 869

P05164 ------------------------------------------------------------

Q1YMS2-B DIILGGFGDDTLMGGSGSDLIKGDAGRDMIFGGAGDDVVL--------------- 780

Q0G341-B -------------------------------------------------------

A6E280-B DMIFAGDGDDNVLAGSGRDMIFGDGGNDRLFGEGGDDFIEGG------------- 825

B7KW13-B -------------------------------------------------------

C7CGY0-B DTILGLDGDDNILAGGGADVVRAGAGNDFVDAGEGRDV----------------- 818

A9W3A5-B -------------------------------------------------------

Q13AU2-B DVIAADAGADAISAGDGNDFVTAGAGRD--------------------------- 774

Q07SX1-B DVIIADAG----------------------------------------------- 768

A3XF15-B DILSGHEGNDMLVG----------------------------------------- 801

B8HDW8 DVVTDLAGDDILKG----------------------------------------- 954

A0JUB7 DRITDSHGDDVLKG----------------------------------------- 875

B1Y442 DVISNSGGFDLLFGGDGKDFILGGQGDAESFGGHGDDFIHAGTG----------- 946

C6MFN2 DFINGGAGINLILAGSGHDFVVTGNDISEVFAGPGNDFIFGNA------------ 934

A6FV45 -------------------------------------------------------

A6FKA5 DVISAGGGIGELMFGGKGND----------------------------------- 886

A3XF15-A -------------------------------------------------------

A5PER4 DVIANSNG----------------------------------------------- 998

Q11K84 DVIANANGLDVLMGGD--------------------------------------- 902

B7KW13-A -------------------------------------------------------

A9W3A5-A -------------------------------------------------------

C7CGY0-A VPEAVAVTNGSFEGLALVAGQEGVILDGNGN------------------------ 1031

Q13AU2-A -------------------------------------------------------

Q07SX1-A DVIANSNGIDILMGG---------------------------------------- 999

Q1YMS2-A -------------------------------------------------------

Q0G341-A DVMATANGLDVLMGGEGKDAIFLGADASEVFGGEGD------------------- 878

A6E280-A DVMASAN------------------------------------------------ 897

B7L1G6 DVIHGGSGLSLLFGNQGNDFIVTGPDGKEAFAGTG-------------------- 1010

B7KRS2 DVIHGGSGLSLLFGNQGNDFIVTGPDGKEAFAGTG-------------------- 1010

Q88JT6-A -------------------------------------------------------

A5W572-A -------------------------------------------------------

B0KJL7-A DVIVAGS------------------------------------------------ 944

Q88JT6-B DVLHGGNGVNLIIGGFGNDFIVTGEDASEAIGGQGNDFILGSKA----------- 1015

A5W572-B DVLHGGNGINLIIGGFGNDFIVTGEDASEAIGGQGNDFILGSKA----------- 1015

B0KJL7-B DVLHGGNGVNLIIGGFGNDFIVTGEDASEAIGGQGNDFILGSKANEQDMGNEGDD 1026

B5IMQ8 DIVLSGSG----------------------------------------------- 877

P05164 -------------------------------------------------------
