# Supplementary material for: Identification of a Novel Calcium Binding Motif Based on the Detection of Sequence Insertions in the Animal Peroxidase Domain of Bacterial Proteins
Source: PLoS One. 2012 Jul 13;7(7):e40698. doi: 10.1371/journal.pone.0040698 (PMC3396595; doi:10.1371/journal.pone.0040698)

**Lignin peroxidase: pdb 1B80** GaDGsimifDD


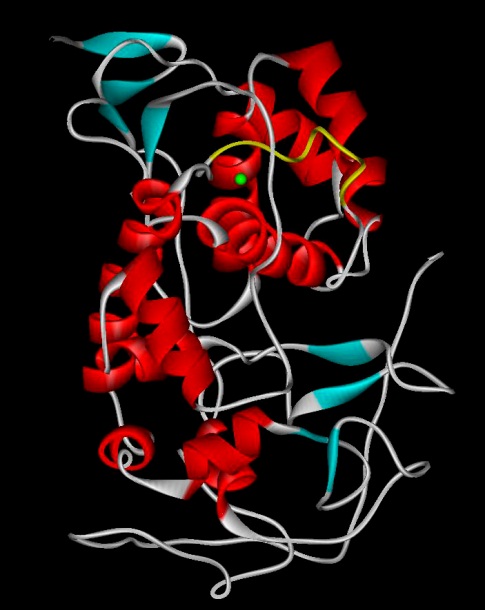

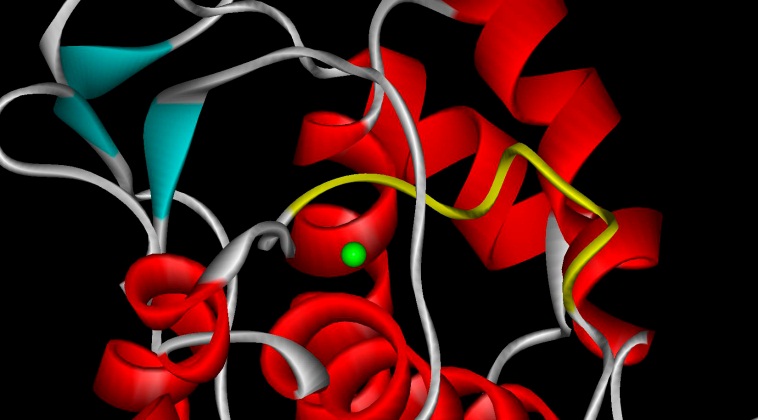


**Acetate kinase: pdb 1G99** GlDGigikiDD


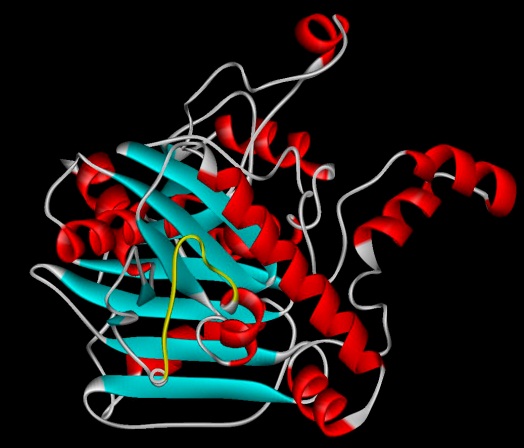

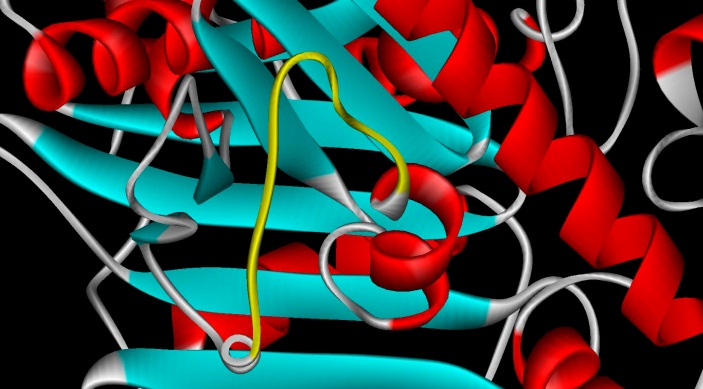


**Amine dehydrogenase: pdb 1JJU GpDGqpGTgDD**


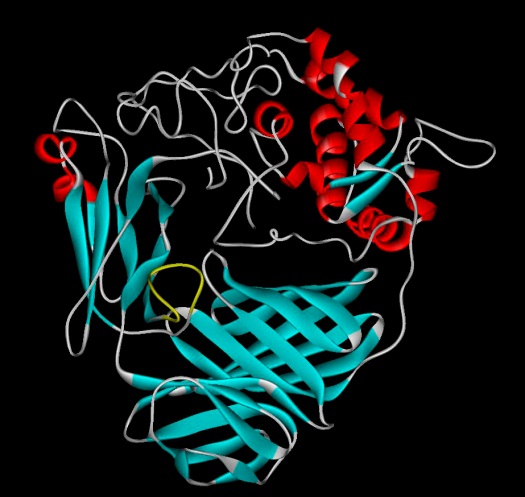

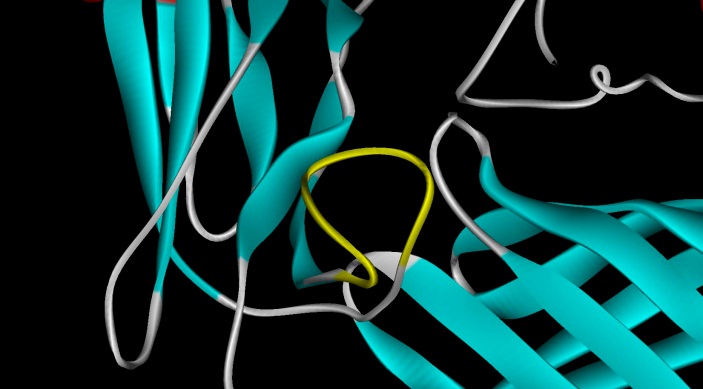


**Protein of unknown function: pdb 2I6E** GyDGvlrigDD


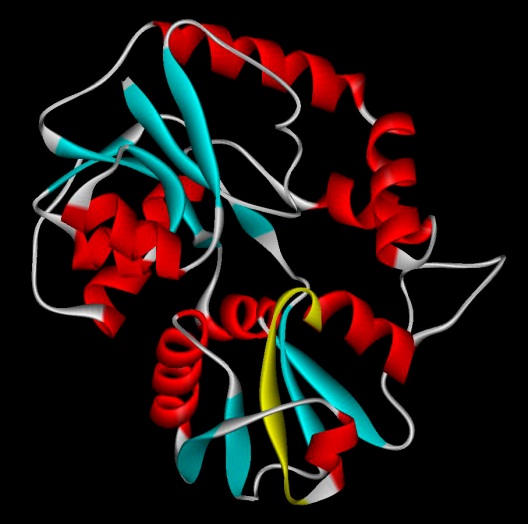

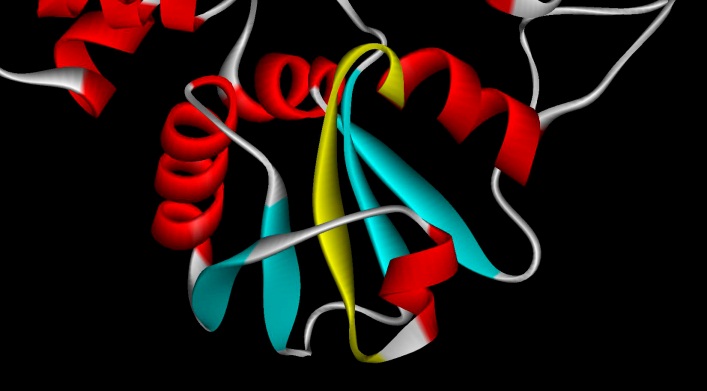


**Pseudopilin: pdb 1T92** GpDGvpesnDD


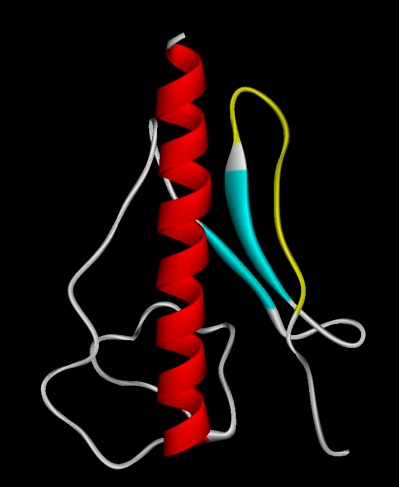


**Pseudopilin: pdb 3G20 GpDGvpNTeDD**


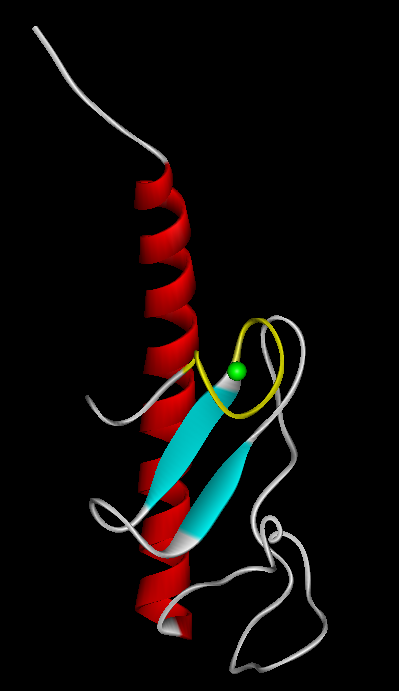

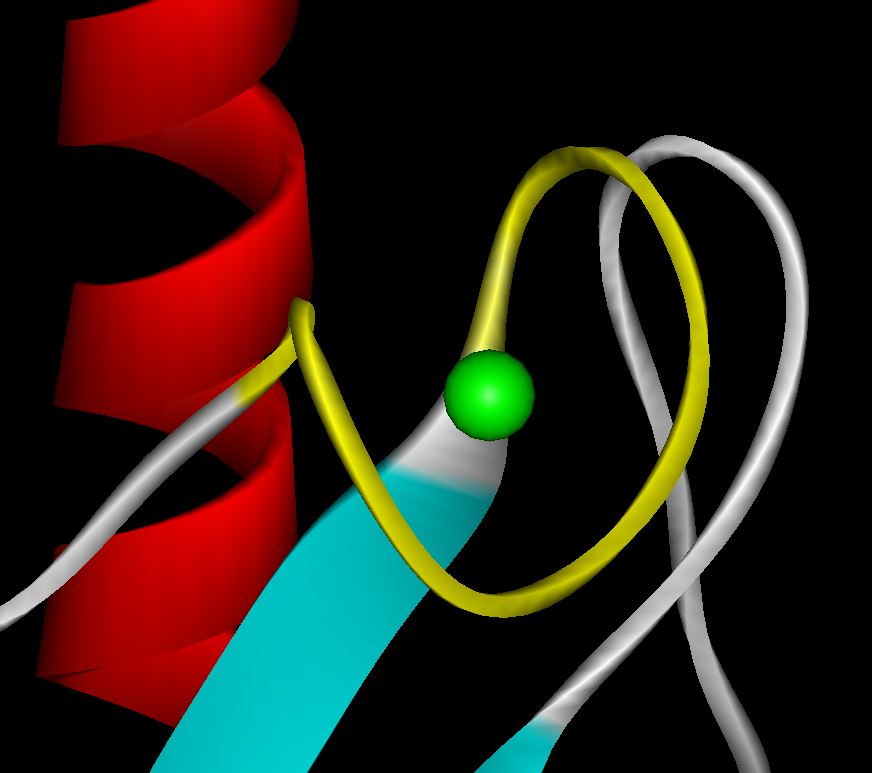

Supplement: Figure S3 — Structure of the fragment that contains the G-x-D-G-x(5)-D-D consensus motif in 6 different three dimensional structures (shown in gold). Bound calcium is shown in green. Motif sequence used in the initial search of PDB was G-x-D-G-x(5)-D-D (see the text in the main body of this article). Amino acids that correspond to the consensus defined are shown. pdb 1JJU and pdb 3G20 match PERCAL (G-x-D-G-x-x-[GN]-[TN]-x-D-D) and their motifs are highlighted in yellow. The figure was produced using the program WebLabViewer (http://www.marcsaric.de/index.php/WebLab_Viewer_Lite). (DOCX) [file pone.0040698.s003.docx]
