# Supplementary figures and images for: Identification of a Novel Calcium Binding Motif Based on the Detection of Sequence Insertions in the Animal Peroxidase Domain of Bacterial Proteins
Source: PLoS One. 2012 Jul 13;7(7):e40698. doi: 10.1371/journal.pone.0040698 (PMC3396595; doi:10.1371/journal.pone.0040698)

I


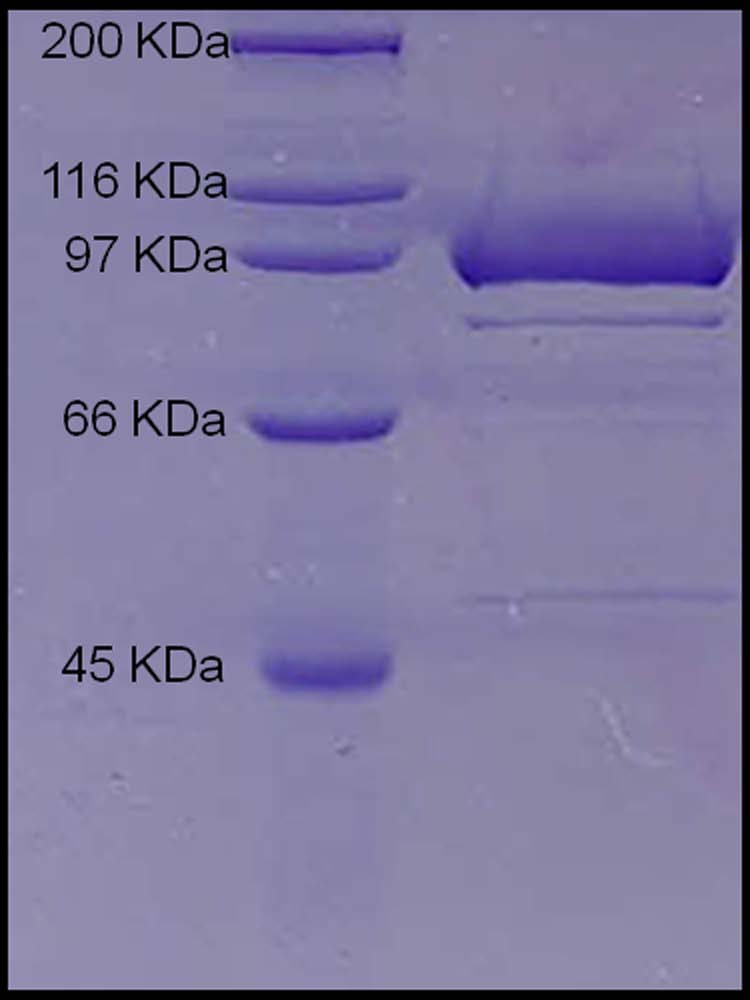


II


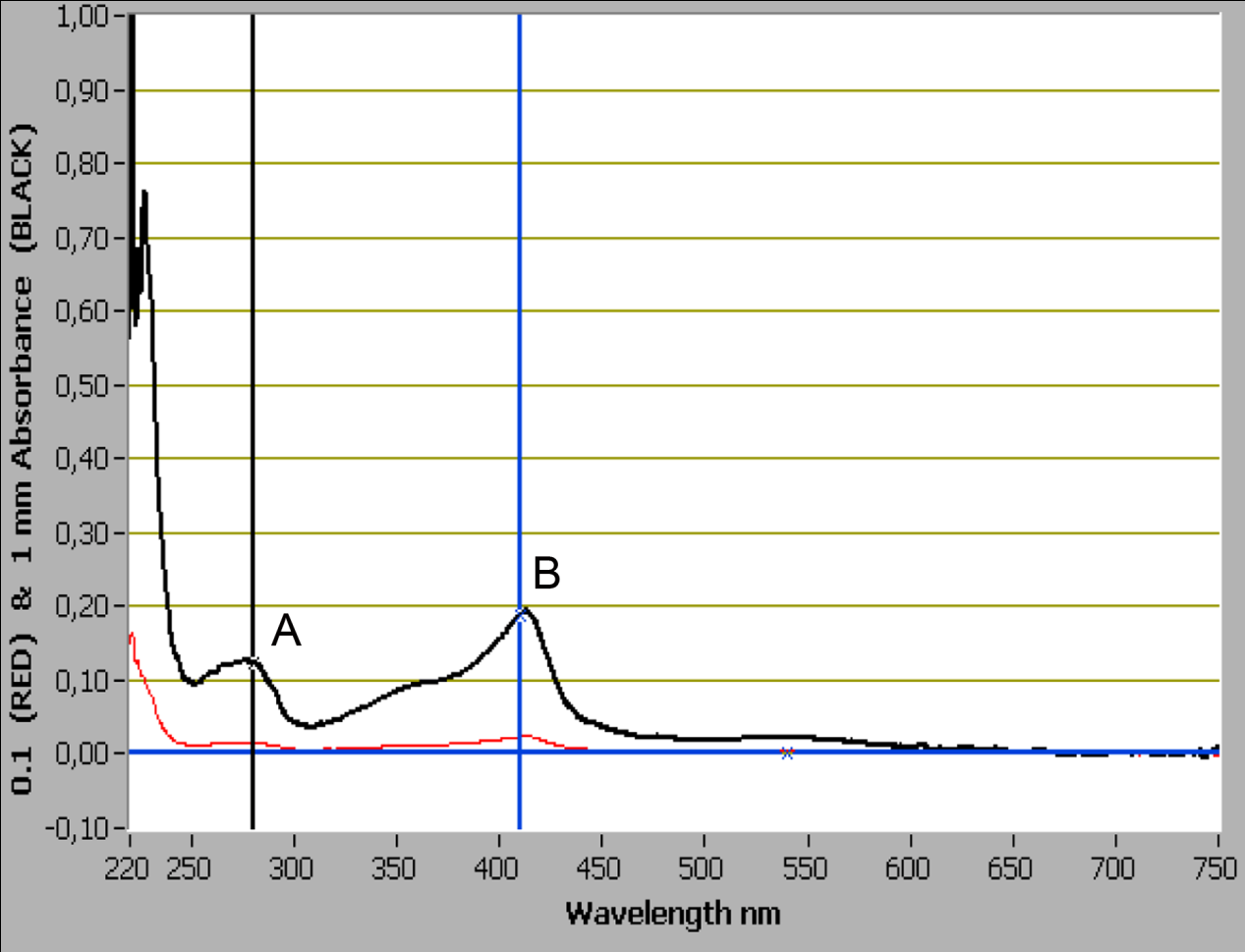

Supplement: Figure S4 — Preparation of recombinant PepA-Nter. I, SDS-PAGE gel of the purified His-tagged Nter-PepA and II) spectrum of the heme-reconstituted PepA N-terminal domain. The purified N-terminal domain was incubated with hemin and exceeding heme was removed with a Sephadex G-25 column equilibrated with 10 mM Tris-HCl, 50 mM NaCl, 10% DMSO and 10% glycerol buffer (pH = 7.5). Displayed is the optical absorption spectrum of the heme reconstituted protein where (A) is the absorbance exhibited by the protein at 280 nm and (B) is the absorbance of bound heme at 410 nm. (DOCX) [file pone.0040698.s004.docx]
