# Supplementary material for: Identification of a Novel Calcium Binding Motif Based on the Detection of Sequence Insertions in the Animal Peroxidase Domain of Bacterial Proteins
Source: PLoS One. 2012 Jul 13;7(7):e40698. doi: 10.1371/journal.pone.0040698 (PMC3396595; doi:10.1371/journal.pone.0040698)
Supplement: Text S1 — Consensus sequences of the insertions interrupting six P. putida ANP-like domains. (DOCX) [file pone.0040698.s007.docx]

Consensus sequence of the 18 more evident insertions in six *P. putida* ANP-like domains. (Insertions A, B and C as identified in Figure S1)

Insertion A

GADGVVGTADDS

GADGVVGTADDS

GADGVVGTADDG

GPDGILGNADDI

GPDGILGNADDI

GPDGILGNADDI

Insertion B

NRDL**GADGKFGTADD**GNGES

NRDL**GADGKFGTADD**GNGES

NRDL**GADGKFGTADD**GNSEN

NRDL**GADGRFGTADD**TEI

NRDL**GADGRFGTADD**TEI

NRDL**GADGKFGTADD**TEI

Insertion C

AGADGIAGTADDIAGVTVT

AGADGIAGTADDIAGVTVT

AGKDGIAGTADDIAGVTVS

PGKDGVLGTADDLKAVTVT

PGKDGVLGTADDLKAVTVT

PGKDGVLGTADDLKAVTIT

1 ----GADGVVGTADD--S---- 12

2 ----GADGVVGTADD--S---- 12

7 NRDLGADGKFGTADDGNGES-- 20

8 NRDLGADGKFGTADDGNGES-- 20

9 NRDLGADGKFGTADDGNSEN-- 20

10 NRDLGADGRFGTADD--TEI-- 18

11 NRDLGADGRFGTADD--TEI-- 18

12 NRDLGADGKFGTADD--TEI-- 18

3 ----GADGVVGTADDG------ 12

4 ----GPDGILGNADDI------ 12

5 ----GPDGILGNADDI------ 12

6 ----GPDGILGNADDI------ 12

13 ---AGADGIAGTADDIAGVTVT 19

14 ---AGADGIAGTADDIAGVTVT 19

15 ---AGKDGIAGTADDIAGVTVS 19

16 ---PGKDGVLGTADDLKAVTVT 19

17 ---PGKDGVLGTADDLKAVTVT 19

18 ---PGKDGVLGTADDLKAVTIT 19

* ** *.***

Aligment perfomed with Clustalw2


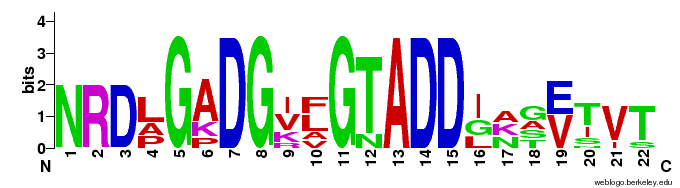


Weblogo sequence of the 18 insertions

Consensus sequences of 30 insertions G-X-D-X(6)-D-D in three *P. putida* animal heme peroxidases (Insertions 1, 2=A, 3=B, 4 and 5=C as identified in Figure S1)

Insertion 1

GIDGVFGTADD

GIDGVFGTADD

GIDGVFGTADD

GMDGQFGTTDD

GMDGQFGTTDD

GMDGQFGTTDD

Insertion 2=A

GADGVVGTADD

GADGVVGTADD

GADGVVGTADD

GPDGILGNADD

GPDGILGNADD

GPDGILGNADD

Insertion 3=B

GADGKFGTADD

GADGKFGTADD

GADGKFGTADD

GADGRFGTADD

GADGRFGTADD

GADGKFGTADD

Insertion 4

GADGIAGTADD

GADGIAGTADD

GADGIAGTADD

GLDGIAGTADD

GLDGIAGTADD

GVDGIAGTADD

Insertion 5=C

GADGIAGTADD

GADGIAGTADD

GKDGIAGTADD

GKDGVLGTADD

GKDGVLGTADD

GKDGVLGTADD


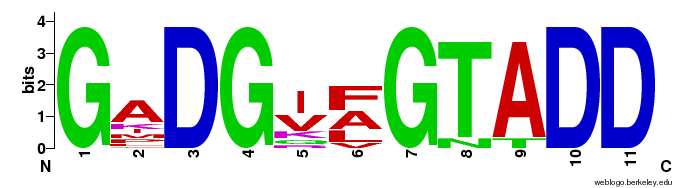


Weblogo of the 30 insertions
