# Supplementary material for: Identification of a Novel Calcium Binding Motif Based on the Detection of Sequence Insertions in the Animal Peroxidase Domain of Bacterial Proteins
Source: PLoS One. 2012 Jul 13;7(7):e40698. doi: 10.1371/journal.pone.0040698 (PMC3396595; doi:10.1371/journal.pone.0040698)
Supplement: Text S2 — Sequences matching X-X-X-G-X-D-X(6)-[DE]-[DE]-X-X-X used to generate the consensus of figure 3 . (DOC) [file pone.0040698.s008.doc]

Sequences within ANP-like domains matching X-X-X-G-X-D-X(6)-[DE]-[DE]-X-X-X in 24 bacterial heme dependent peroxidases (their Uniprot Code and location in the sequences are in Table S1)

mspGiDGvfgtaDDkpv

rtaGaDGvvgtaDDsqp

rdlGaDGkfgtaDDgng

vnnGaDGiagtaDDvtq

tlaGaDGiagtaDDiag

aspGmDGqfgttDDhdv

nlpGpDGilgnaDDire

rdlGaDGrfgtaDDtei

vmkGlDGiagtaDDqev

tlpGkDGvlgtaDDlka

hlpGeDGvlgtaDDivs

tglGpDGipntaDDivv

sdpGpDGirgtgDDvia

mspGiDGvfgtaDDkpv

rtaGaDGvvgtaDDsqp

rdlGaDGkfgtaDDgng

vnnGaDGiagtaDDvtq

tlaGaDGiagtaDDiag

aspGmDGqfgttDDhdv

nlpGpDGilgnaDDire

rdlGaDGrfgtaDDtei

vmkGlDGiagtaDDqev

tlpGkDGvlgtaDDlka

mspGiDGvfgtaDDkpv

rtaGaDGvvgtaDDgqp

rdlGaDGkfgtaDDgns

vnnGaDGiagtaDDvtt

tlaGkDGiagtaDDiag

aspGmDGqfgttDDhdv

nlpGpDGilgnaDDihe

rdlGaDGkfgtaDDtei

vmkGvDGiagtaDDqqv

tlpGkDGvlgtaDDlka

lvlGqDGlagtaDDlap

slpGpDGirgtaDDivp

liaGdDGilgnaDDlpp

iapGaDGvvgtsDDvrt

lvlGqDGlagtaDDlap

slpGpDGirgtaDDivp

rthGpDGiagtgDEvpg

iglGdDGipntaDDlva

aqpGpDGirgtaDDivp

lvlGqDGlagtaDDlap

slpGpDGirgtaDDivp

rthGpDGvagtgDEvse

vgiGaDGipntaDDiva

aqpGpDGirgtaDDivp

nqpGpDGvlgtaDDeqn

dgpGpDGvlgtaDDtsr

vgiGpDGipntdDDivm

tlpGpDGvrgteDDivn

liaGpDhdfgtaDDlnp

nqpGqDGilgtaDDlqd

dspGpDGvwgtqDDvpg

fdaGpDGipftaDDgrt

vgiGaDGipntaDDvvv

tdpGpDGirgteDDiln

nqpGpDGilgtaDDvre

nkpGnDGimgtgDDvrd

lgvGpDGipntaDDivv

tlpGpDGikgteDDvtp

veaGaDGilgtaDDgag

vgmGpDsllgtaDDvlr

veaGaDGilgtaDDgag

vgmGpDGllgtvDDvlr

lvlGaDGiagtaDDlpn

dgpGaDGvlgtaDDtqh

vgiGaDGipntaDDdvv

adpGaDGirgteDDvvr

fdaGaDGipgtaDDgpn

nqpGpDGilgtaDDire

nqpGpDGilgtaDDire

gilGdDpltvgvDEsld

niaGkDktfsvmEElen

Consensus in 74 intradomain insertions: 68, x-x-x-G-x-D-G-x(5)-D-D-x-x-x (black); 2, x-x-x-G-x-D-G-x(5)-D-E-x-x-x (blue); 4, x-x-x-G-x-D-{G}-X(5)-D/E-D/E-x-x-x (green)


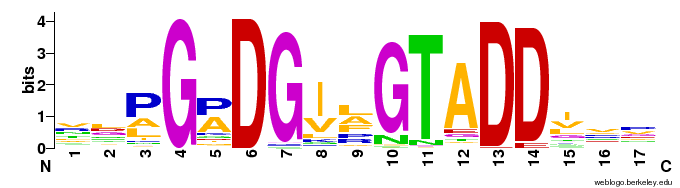


Sequence present in pseudopilin

DIDIFSPGPDGVPNTEDDIGN

Sequence of oligopeptide BACHEMP-CONS: core with the weblogo obtained above and the flanking residues corresponding to the C and N terminal ends of two beta strands surrounding the motif in pseudopilin

[H] DIDI**VLPGPDGILGTADD**IGN [OH]
