# Supplementary material for: Identification of a Novel Calcium Binding Motif Based on the Detection of Sequence Insertions in the Animal Peroxidase Domain of Bacterial Proteins
Source: PLoS One. 2012 Jul 13;7(7):e40698. doi: 10.1371/journal.pone.0040698 (PMC3396595; doi:10.1371/journal.pone.0040698)
Supplement: Text S3 — Sequences of the ANP-like domains of bacterial proteins used in this work according to Prosite profile PS50292. (DOCX) [file pone.0040698.s009.docx]

In red ANP-like domain (according to Prosite profile PS50292)

>B8HDW8

QLASPLLSHGLRTVDGSCNNLQPGQDTYGASDQVFPRLAPKAFGPAESGSFGGPPVATSYTQKSGSVFDSRPRTISNLIADQTSTNPAAVAAAGFPARSQGNTGVVPCTTDPDAEAVPPVAAAPEGCVPSHNTLDIPNVTTDVGLSPPYNSLFTLFGQFFDHGIDQTVKGGGTVYVPLKADDPLIAGPDHDFGTADDLNPHLRFMVLTRGQNQPGQDGILGTADDLQDALNTNSPWVDQSQTYASHSSHQVFLREYTNNPEGRPVSTGGLLGGPAGTAAAGGMATWADTKKQAREMLGIQLLDKDALNVPLLAADAYGKFIPGPKDGLPQFVTRSGLVEADRTANGGNGTLVPQDILYFNTPFLTDIAHNADPSPQDTDHNPATPPAAPAPDADNTASADFAAQAPGTYDDEMLGAHFIAGDGRVNENIGLTAIHQVFHSEHDRLVGDIKNVLTSDKSSRGTAALTEWRATAGADGWNGERLFQAARFIAEMEYQHLVFEEFARKIQPAVNIFEPFAFSQTDVNPAINAEFAHSVYRFGHSMLTETISRRNEDSPGPDGVWGTQDDVPGSQNDLPLLGGFLNPPAYTDGGPAGPLTSEEAAGSIVMGMSDQVGAELDEFVTDTLRSKLLGLPMDLAAINLARGRSEGIPALNVFRRQLHGATNDSQLKPYANWIDFGENIKHPASLINFIAAYGTHPSIVSATTLDAKRKAARLIVSPDALAGEVAPDDAVAFMNSTDAWANNGTASTTGLDDIDLWMGGLAERTNMFGGLLGSTFNYVFESQMTDLQNGDRLYYLARTPGMNLMAQLEGNSFAELIMRNTNAKALKADAFATADCKFELKNLAGTSEGFAASGNTVADDAGTECSETALLLRMPDGTIKYRASNSVDPVGINGQAVYNGTDRADRVHGGVDNDTFWGGKGNDVIEGGDGADTVLGGEDNDVVTDLAGDDILKG

>A0JUB7

QVASPLLPYGLRTVDGTDNNLVAGQSGYGSASREFPRLSDPEWRTSSGGQNYESVNANVTDNGPRFVSNVIVDQTATNPAAVAAAGKAHRTVNDGPTAVPCDGNGLPENCVPEGETLDIPNVTTDFGLSPPYNGMFALFGQFFDHGVDFTKKTKNYVMMPLSPDDPLYVPGGRTNFMLLNRAENQPGPDGVLGTADDEQNATNTDSPWVDQSQTYSSHSSHQVFLREYTLNENGDPVSTGELIEGEPGGMATWARIKEQARTMLGLELSDVDVADIPKLATDQYGRFLRGPNGLPQYETATGRVEGNLAAPVAPPANVERIGIAFLDDIGHNAAPFNSQTGAPLQPDDDEDVNGVNEPRPAGRYDDEMLDKHFVAGDGRVNENIGLTAIHQVFHSEHNRLVGYMEELLTSQNLDLNEWKLPNGQWNGERLFQAARYVTEMEYQHIVFEDFARKIQPGINGFNVFTQSDTGIDPAIQAEFAHATYRFGHSMLTETVDRKLNDGTDIGMPLLDAFLNPPAYYESTAGTLNPKQAAGAIAMGMTDQVGAELDEFVTDTLRNNVLGLPLDLASLNLARGRDTGIPSLNNFRTQLYASTGESSLKPYTSWVDFGQNLKHPDSVVNFMAAYGTHETITAAASITDKRAAAQRLFDMDTADPATPADSYDFVNSAGAWASQPSGLNNVDLWVGGLAERQNLFGGLLGSTFNYIFERQMTDLQDGDRLYYLSRTSGLNLRTQLEGNSLAELIMRNTDAEALKADVFGVADCEFELGRITAGTGNSVADDPASACDESALLMRMSDGTIRYRVSNTVDRPGLNAQSTFNGTALGDRIWGGIDNDTFWGNDGQDIIEGNDGADTVLGGDGNDRITDSHGDDVLKG

>B5IMQ8

ADSSTSNPAAVAAAATQNSLLEAPVPMPGQNVNAFGVPLGDDGQELGIENFGGGDTIEIRNVAPLPINAPYNSWLTLFGQGFDHGLDFINKGGNGNVFIPLDPSDSLFDAGADGIPGTADDGPNFMVVTRATRDFANGGATQNVTTPWIDMNQAYGSHASYQVFLRDYTVNSTFGSDGDPGFSPISGLQLNQEFGAVISTGNMLASTRTGSGLPTWADIKANAESMLGFVLTDDFVLSSPLLATDDYGRFIPGASGLPQVVVETAPGVFTLVEGNLTTPVDLSAIPGGGTVVRTGHAFLLDIAHPANPTFNPDGTVDPTSYDPALLNEHFIAGDGRVNENQGLTAFHTIFHNEHDRLVADYKRIALEAAASGDLSFINAWLRPGRQLASGDPVPGVDSDAWDGERLFQAARFMVEMQYQHGVFEEFGRFIDPNIALDSGFNVTLNPAITAEFAHAVYRFGHSMLTESVDRFGIDPVTGDWSNNSESLIAAFLNPVGFLQSLDAVGTTQDLTPEEAAAEVLRGMTRQQGNDIDEFVTPALRNNLVGLPLDLATINIARGNELGLPKLNAARAEFFSLTGGNPSLTPYSNWNDFRSHLKHDASIYNFVAAYGTHPTITSATTVEAKRAAAMDLVDYVHPDGPAFMNAQGGFAGQRAGLDNVHLWIGGLAEAPEAGVTMLGTTFAFVFEQQLQALQNADRLYYLRRLNGNLLTQIENNTFAAMAERALGGAITSLPGHIFSLPTYTLEVDAATQHTNLGLDGKADPVGGAVSSNLQEVYRTSQGDPNHSTISQIGFNVANVLRYNGLGHVVLGGGAEADLLQAGSGNDTVYGRGGNDVIFSGQGDDQVWGGEGNDFISDNFGLNFLRGEAGHDIVLSGSG

>A5PER4

LGNFHLPYGLRTVDGSYNNLVEGRERWGASDEIMPRMFTPNYRDDQDGDMMSFGPGMTITNTDYGAAGDVADVDPRIISNLIVDQTAGNPAAISAALTQTGYEGDATAAIQALHSAWADHENGLMTYEEFATLALDTYGLEMQGDTLVIPNTAPDEGLSSPFNAWMTYFGQFFDHGLDLIPKADNGTVYVPLQPDDPLFDAGPDGIPFTADDGRTNFMVMTRAQIDENGETMNTTTPFVDQNQTYGSHASKQVFMREYELVNGRPEATGHLLEGVNGGLATWADVKAQALDVLGIVLSDADVLALPLIRSDPYGNFIPDENGYPQVVVGIGADGIPNTADDVVVSGSPTNPVHLLTGMAGEIPPEGVPEGMAVIPVRTAHAFLDDIAHNAVPVFDEGNLAADADTDTGNAVATGTRGENLEYDNELLDRHFITGDGRGNENIALTAVHHVFHSEHNRQIDSNKIEILKSGDLAFINEWLDVDITAPEVAGLAALTDAQLAAYGESLDWDGERLFQMAKFSTEMQYQHLVFEEFGRRIQPNIDPFVFNTTTDINPAIFAEFAHVVYRFGHSMLTEDVNRMFLNDAGEPVFYDELGNETPVTDLEGWGNNTGLIEAFLNPVDYDLDGNISAEQAAGAIFRGLNREQGNHIDEFVTDALRNNLLGLPLDLAAINIARGRDTGMPTLNEARAELYDATGSTFLTPYGSWAEFAEGLKNPLSVINFIAAYGTHQTIIDAGDNVDARRAAATDLVLGGGAVSEADRLAFLNGPAAETGVNDIDMWIGGLAEAPMAFGGFLGSTFNAVFEAQMEALQDNDRFYYLSRTQGLNFLNELENNAFSKMVMANTDMTDPGPDGIRGTEDDILNYHSHVDAFTMADFLLHVDPTKQIGPDPQHDDEVLNSIGQTLVQRDDLATAEVEVNYIKFIGADHTTINGTAGDDTIIAGGGDDGIWGGAGNDRIEGGHGVDLIIAGAGNDIVTDSGDSDDFIKGDEGDDVIANSNG

>B1Y442

LVTSPLLPYGLRTVDGSHNNIVPGQEEFGAADRPMPNALQQFFRTAEMGDVDGPGPGGLVQTTYANATGGLVFDSQPRVISNLISDQTITNQAAVDVATRFADGEPVVISDEGTYFIQNVAPDEGLSAPFNSWFTIFGQFFDHGLDLIGKGAGTVFIPLKPDDPLIAGDDGILGNADDLPPQLQFMALSRAAASNIAPGADGVVGTSDDVRTFSNSTTPWIDQNQTYTSHASHQVFLREYTLVGGRPESTGRLLDGAVEGSIGNWAEVKAQALMLGIRLTDADIGNVPVLLTDEYGKFIPGSNGYAQLVMANGSIVQGTAAGILTTGSAKTGHAFLDDIAHHAVPGFVDTNRDGIQNNGELSQTPDTDTGDVNLDGVVNEADLTADDRIAGTYDNELLDRHFITGDGRGNENIALTAVHSVFHSEHNRLAEQIKELAVSSNDVTFLNQWLDTPVAAVPTTQAGVDALNWNGERLFQAARFGTEMQYQHFVFEEFARKVQPSIDVFSGYNSTVDPAIMSEFANVVYRFGHSMLTETVDRIDPLTGESNPIGLIEAFLNPVEFGAGTTTTAEAVGNIVTGMTRQTGNHIDEFITEALRNNLVGLPLDLAALNIARGRDTGIPGLNAARAMFFEDTGLSSLQPYDSWSDFGLGIKNPESLVNFIAAYGTDASITGATSLADKRAAAQAILDAAELGDAAALAFLEGPAATTGVNDIDFWIGGLAEVTQPFGGMLGTTFDYVFSTTLLQLQNNDRLYYLARTAGLNVLTQLEEGSLAELVMRNSTAKHLPGDIFSTPTYIFEVGTMNPTGRPLSDDPSTPDVNESLLLTRMADGTIRYAGAEHILMGGTDGADRLRAGAGDDTLHGDAGNDRLEGGAGNDFFFAGEGDDILTDSFGDDNMKGQGGNDVISNSGGFDLLFGGDGKDFILGGQGDAESFGGHGDDFIHAGTG

>Q11K84

LEIAEGQAALFTPELVAAQQGLLDTAAATYGLEFEMDGKTIKLPNVAPDEGLSAPFNGWMTFFGQFFDHGLDLISKGGNGTIYVPLEPDDPLYVPGGHANFMVLTRATQFDGPGPDGVLGTADDTSREQINTTTPFVDQNQTYTSHPSHQVFLREYAMVDGKPLATGHMLDGANGGLPTWADIKAQAKNLLGIELTDRDVFNIPLIRTDLYGEFIRDENGFPQVIVGIGPDGIPNTDDDIVMSGTPDAPLKLNPADGGTEPVRTSHAFLDDIAHNAVPVLDTQGFLRPDGVDLPVDPAGNAVQFDPLTGHNLEYDNELLDRHFITGDGRGNENVGLTAVHHVFHSEHNRQVEAQKLEILKSKDLAFINEWLLTDLASVDDIPAGGAALANYAATLTWDGERLFQSARFATEMQYQHLVFEEFGRKVQPLIDLFIFNTITDIDPAIFSEFANVVYRFGHSMLTEDIGRMFLNDAGEPVTYSRDDEGNLIETVVTDLTAWGDDIGLIEAFLNPIEFDQNGQISHEQAAGAIFRGMTLVHGNEIDEFVVDALRNNLLGLPLDLAAINIARGRDTGMPTLNQARTQLYEASNSTFVKPYANWTDFAANLKTPASIINFIAAYGTHDLILAAEDDVAARRAAATLIVLGGQDAPADRLEFLNGTGAWSGVETGLNLVDLWIGGLAEKKMPFGGMLGSTFNAVFEAQLEMLQDLDRFYYLTRTQGLNLLNELENNAFSKLVMANTDMTLPGPDGVRGTEDDIVNYHVGVDSFAKHDLVLHVDPDKQIGDDPTHEDPALNAVGLTKVQRDDLTTTGPDENYIRFLGGEHVVIGGTEGDDTIISDFGDDALWGGGGNDRIEGGAGVDLIIGGSGDDIITDSGDTGDFIKGEDGDDVIANANGLDVLMGGD

>B7L1G6

LTQAHLLPYGLRTVDGSYNNLLPGRENWGASDQSFPGLFTPTYINDADGDRYDFNPLPNVETWYSNNDYANAGVRSGSQPGPGSGTVIDADPRIISNLIVDQTLDNPAAIAAALTHAGLAGQPLMTALSEIVGAHNAAKTTPNDPAATAALDAKLAQYGVEMDGNTVFLPNVSPDEGLSSPFNGWMTIFGQFFDHGLDLVAKGGNGTVYVPLSPDDPLYVPGGQNYIPLTRVTVEAGADGILGTADDGAGPKNLTTPWVDQNQTYASTASKQVFMREYIPGPDGKPIASGHLLEGSNGGLATWADIKAQAKTVLGIELTDLNVGNIPLVAADPYGNFIPGPNGYPQLVVGMGPDSLLGTADDVLREGNPAAPVSAQGVVLTGHAFLDDIAHAAVPVIAGGVLQADGDAALGYASADGAPGPQGPRGATAYDNELLDRHFVAGDGRANENIALTAVHQVFHSEHNRVVEMTKQIALDSGDLAFLNQWLLVDVATIPISEADRAALVWDGERLFQAGRFTNEMEYQHLVFEEFGRMMQPDIDAFVFEPSADINPSIAAEFAHVVYRFGHSMLRQDIAVIGMDADGKPVQNDISLFDGFLNPIMYDSLGDAEAASGAIIRGMSRQTGSEIDEFVTDVLRNQLLGIPLDLATINLARGRDVGTPTLNVARERFFEETGDTLLKPYESWADFALSLKNPASIINFIAAYGNHASVLNTADAPRTVADLRAAATKLVLGDSALTGDAKAAFDADRLDFLNHTGAYAGDGSLGGLNTVDFWMGGLAEKKMAFGGMLGSTFSFVFQMTMENLQDADRFYYLSRTQGLNLLNELENNTFAELVMRNTDLGDAHSTALPGNLFSAFQMPTLELDISKQLGADPVSDDPFLGGFSKLIERVDANGDGIAESIRVNSNEHFTIGGTEGNDIIVSGGGDDAIWGKAGDDRIEAGYGVDKVFGGAGDDIITNAGTDIGEADFLHGNEGNDVIHGGSGLSLLFGNQGNDFIVTGPDGKEAFAGTG

>B7KRS2

LTQAHLLPYGLRTVDGSYNNLLPGRENWGASDQSFPGLFTPTYINDADGDRYDFNPLPNVETWYSNNDYANAGVRSGSQPGPGSGTVIDADPRIISNLIVDQTLDNPAAIAAALTHAGLAGQPLMTALSEIVGAHNAAKMTPDDPAAIAALGAKLVQYGVEMDGNTVFLPNVSPDEGLSSPFNGWMTIFGQFFDHGLDLVAKGGNGTVYVPLSPDDPLYVPGGQNYIPLTRVTVEAGADGILGTADDGAGPKNLTTPWVDQNQTYASTASKQVFMREYIPGPDGKPIASGHLLEGSTGGLATWADIKAQAKTVLGIELTDLNVGNIPLVAADPYGNFIPGPNGYPQLVVGMGPDGLLGTVDDVLREGNPAAPVSAQGVVLTGHAFLDDIAHAAVPVIAGGVLQADGDAALGYANVDGTPGPQGPRGATAYDNELLDRHFVAGDGRANENIALTAVHQVFHSEHNRVVEMTKQIALDSGDLAFLNKWLLVDVEAMPTTEAERAALVWDGERLFQAGRFTNEMEYQHLVFEEFGRMMQPDIDAFVFEPSADINPSIAAEFAHVVYRFGHSMLRQDIAVIGMDADGKPVQNDISLFDGFLNPVMYDSLGDAEAASGAIIRGMSRQTGSEIDEFVTDVLRNQLLGIPLDLATINLARGRDVGTPTLNVARERFFEETGDTLLKPYESWADFALSLKNPASIINFIAAYGNHASVLNTADAPRTVADLRAAATKLVLGDSALTGDAKAAFDADRLDFLNHTGAYAGDGSLGGLNTVDFWMGGLAEKKMAFGGMLGSTFSFVFQMTMENLQDADRFYYLSRTQGLNLLNELENNTFAELVMRNTDLGDAHSTALPGNLFSAFQMPTLELDISKQLGADPVSDDPFLGGFSKLIERVDANGDGIAESIRVNSNEHFTIGGTEGNDIIVSGGGDDAIWGKAGDDRIEAGYGVDKVFGGAGDDIITNAGTDIGEADFLHGNEGNDVIHGGSGLSLLFGNQGNDFIVTGPDGKEAFAGTG

>C6MFN2

DPLLPFGLRNVDGTYNNLEDGQSLFGSADQVFPRGLSIFLRPGEQITFDPDGPGPQTVGQSTHYNQTKGAVFDSQPRTVSNLISDQTATNPAAIAVANGTPGSELVNGTRADGSAFQTYFIPNVTPDAGLSAPYNSWFTFFGQFFDHGLDLVNKGQSGTVFVPLLDDDPLVTGPDGILGDDPLTVGVDESLDDLPEGLRFMALTRATNKPGNDGIMGTGDDVRDHVNQTTPFVDQNQTYTSHPSHQVFLREYELNGAGRPVSTGNLLEDSTTGDGLATWADIKAQAQNMLGINLTDADLTNLPLLATDTYGKFIPGSNGFVQIVTSSGLVQGNPAANGGNGIDIPGNAVRTGHAFLDDIAHTAVPKFASGLLNPDPDPDTGNTPAPGTYDDELLDAHFITGDGRGNENIGLTTVHTVFHAEHNRKVQEIKQLITASGDTPFMNQWKLPNGNWDGDKLFQAARFATEMQYQHLVFEEFARKVQPNVDVFIGYDSSIDPAIMAEFAHVVYRFGHSMLTEKVERIDAQGDSNDIGLIQAFLNPVEFGADYANNSEAAAAVVRGMTKQVGNEVDEFVTEALRNNLLGLPLDLPTINITRGRDTGMPGLNDARRMFFDDVGHPSLAPYESWNDFKLDLKNPESLTNFIAAYGNHPSINSVTTMEDKRAAADLLVKGGPGAPADRANFMNSTGAYTTANSGLDSVDFWIGGMAEKQAPFGGLLGSTFNYVFETQMENLQNGDRFYYLARTAGMDFLTQLEENSFAEMIMRNLPDVKHLPFDVFSTPTYIFEASAQTVSGTIIDDPDTTTYDERDLLIRDTSLGPDTIRYTGEDHVVMGGTESNDRLRAGAGDDTLWGDGGNDRLEGGAGNDSINGGDGNDRITDASGDDNIKGGDGHDFINGGAGINLILAGSGHDFVVTGNDISEVFAGPGNDFIFGNA

>A6FV45

AAEGVEATKQVAVDTATSDLNALTAPGAAEAALAAAQATAAEAQTTLDTLLTTHNITMDGNNVLLPDVTPDEGLSAPYNSWMTLFGQFFDHGLDLVGKGGSGTVYIPLQPDDPLYDANSPTNFMVLTRATNQPGPDGILGTADDIREHFNKTTPWVDQNQTYTSHPSHQVFLREYDIDANGNPVSNGYLLHGQTGGMATWGDVKAQAATKLGIQLNDSDVLDGPLLATDPYGNFIPGANGLPQLVVPNPAYVEGGTEPLNILIEGDLANPVDASQALRNGHAFLEDISHTAFPKGMIDHDRNPMTPEIEVLPDADTDTGNAIMPNIFGMNETYDNELLDRHFIAGDGRGNENFGLTAVHHVFHTEHNRQAAEMKQTILDSGELAFINEWLATPITEADLATATVDTVEWDGGRLFQAAKFTTEMQYQHLAFEEFGRTVQPQIAAFGVNGSAEIDGAVMAEFAHVVYRFGHSMLTENVHTMDPNGVNTSNGLIEAFLNPVAFDLDQTLTSDQAAGAVARGMSRETGANIDEFITSALRDNLVGLPLDLAALNIARGRETGVPSLNAAREQFYSATGSEFLKPYEGWSEFAANLKNPASIINFIASYGTHETIVNATTVVEKRAAATDLVLGGDNAPADRLDFVNGTGAWADAETGINDVEFWIGGLAEDIMPFGGMLGSSFGFVFQQQMEALQNGDRFYYLARTAGMNMIAELENNSFASMIVRNTDIKDGGAHIPANIFSSMDVILEVDQAVQSMPDPVSTDVDPFLAAMGTTMVERATATVDAPLIDGVREYDNFLKFNGGEHAVLGGTDQRDILNGGLGDDALWGGAGDDLLIGDAGVNTLRGGAGNDILKDGDD

>A6FKA5

AAEGVEATKQVAVDTATSDLNALTAPGAAEAALAAAQATAAEAQTTLDTLLTTHNITMDGNNVLLPDVTPDEGLSAPYNSWMTLFGQFFDHGLDLVGKGGSGTVYIPLQPDDPLYDANSPTNFMVLTRATNQPGPDGILGTADDIREHFNKTTPWVDQNQTYTSHPSHQVFLREYDIDANGNPVSNGYLLHGQTGGMATWGDVKAQAATKLGIQLNDSDVLDGPLLATDPYGNFIPGANGLPQLVVPNPAYVEGGTEPLNILIEGDLANPVDASQALRNGHAFLEDISHTAFPKGMIDHDRNPMTPEIEVLPDADTDTGNAIMPNIFGMNETYDNELLDRHFIAGDGRGNENFGLTAVHHVFHTEHNRQAAEMKQTILDSGELAFINEWLATPITEADLATATVDTVEWDGGRLFQAAKFTTEMQYQHLAFEEFGRTVQPQIAAFGVNGSAEIDCAVMADSPMWCTASPLDADRDVHTMDPNGVNTSNGLIEAFLNPVAFDLDQTLTSDQAAGAVARGMSRETGANIDEFITSALRDNLVGLPLDLAALNIARGRETGVPSLNAAREQFYSATGSEFLKPYEGWSEFAANLKNPASIINFIASYGTHETIVNATTVVEKRAAATDLVLGGDNAPADRLDFVNGTGAWADAETGINDVEFWIGGLAEDIMPFGGMLGSSFGFVFQQQMEALQNGDRFYYLARTAGMNMIAELENNSFASMIVRNTDIKDGGAHIPANIFSSMDVILEVDQAVQSMPDPVSTDVDPFLAAMGTTMVERATATVDAPLIDGVREYDNFLKFNGGEHAVLGGTDQRDILNGGLGDDALWGGAGDDLLIGDAGVNTLRGGAGNDILKDGDDVSFLHGEDGDDVISAGGGIGELMFGGKGND

>Q88JT6-A

LPNSQVPFGLRTVDGSYNNLVTGQSEFGAADNSFLRLLEASYRANYVGTGNVVDSQPRTISNLIVDQTANNPAAVEANGGAAPVMSPGIDGVFGTADDKPVFFIPNVSPDAGLTAGFNAWMTFFGQFFDHGLDLVSKSSTDIVFIPLRPDDPLFVAGSPTNFMVLSRAVRTAGADGVVGTADDSQPNTTSPFVDQSQTYSSHPSHQVFLREYTVNAAGDPVATGRLITNRDLGADGKFGTADDGNGESGGMATWAVVKAQARDLLGINLTDADVHSVPLLATDAYGNFLRGPNGMPQVVMRVNNGADGIAGTADDVTQLVEGDRNAPISLANAVSTGHGFLDDIAHNAAPVVVGGALQADADTLVGNAQPVGQGGNNLTYDNELLDAHYIAGDGRVNENIGLTAVHHVFHSEHNRLVQQTKDTLLAAGDLAFLNEWLIDDVTAIPTTPADIAALVWDGERLFQAAKFGTEMQYQHLVFEEFARTIQPQIDEFLAPNGYDTSINPAILAEFAHVVYRFGHSMLTETVDRYDPAFNPVSADPANPDQQLGLIAAFLNPLAFAGSGATADEAAGAIIRGVTRQVGNEIDEFVTEALRNNLLGLPLDLPALNIARGRDTGIPSLNEARREFYAATGDSQLKPYMSWVDFADHLKHPASLINFIAAYGTHSSITGATTEAAKRAAAVALVLGGDGAPLDRLDFLNGTGAYANVTLAGADGIAGTADDIAGVTVTGVDAIDFWVGGLAEKKMPFGGMLGSSFNFVFETQLEALQNGDRFYYLSRTAGMNFGTELENNSFAKLIMANSDVTHLSNTVFLTPTFTLEVNQANQFTGLGADGKADPTGGIEINGVEIVPLVIRDNPDTVGPDSNFLHYTGEDHVVLGGTAGNDIIISSEGDDTLYGDAGDDLLEGGAGNDAVLGGAGDDIITDSFGDNRLEG

>Q88JT6-B

IPNVRAAMGLRAVDGSNNNLMNLNGNNNTQYGAADNVFPRVTDPVFNPAEGAPAGFFGPGSPAIPGSSYQQTSGPVFDSQPRTISNLIVDQTSNNPAAYATAYDPGADGVLNFGAAGNDDVLKDGVRIVASPGMDGQFGTTDDHDVYLFENTAADAGLSAPFNAWMTFFGQFFDHGLDLVTKGGSGTIYIPLQPDDPLYVEGGFTNFMVVTRATNLPGPDGILGNADDIREHTNTTTPFVDQNQTYSSHPSHQVFLRAYVMTDDGPVATGRLITNRDLGADGRFGTADDTEIGGMATWKVVKAQARDLLGINLTDADVDNVPLLATDAYGNFIKGPNGYPMVVMKGLDGIAGTADDQEVEGNPLAPLDLTNAVRTGHQFLADIAHNAVPVFSGGVLAPDADNVAGNAVPVNPQTGANLAYDNELLDAHYIAGDGRVNENIGLTAVHAIFHSEHNRLVAQTMDTVLDSGDLAFLNEWLLNPVSALPVTPAEIGALVWNGERLFQAAKFGTEMQYQHLVFEEFARTVQPRVDLFFAPTQVYDVDLDASIVAEFAHTVYRFGHSMLTETVDRFDIDFNVIQDPASANPDQQLGLIAAFLNPLAYAASGVTPEDATSAIVRGVTRQAGNEIDEFVTEALRNNLLGLPLDLPAINIARGRDVGIPSLNAVRREIYGQTGDTQLKPYTSWVDLVQHLKHPESLINFIAAYGTHSTITNATTLLEKRAAAMALVFGGDGAPADRMDFLNSSGAWANVTLPGKDGVLGTADDLKAVTVTGVDAIDLWIGGLAEAKAPFGGMLGSTFNFVFENQMEKLQDGDRFYYLERTAGLSMNAELESNSFAKLIMANSSATHLPGLVFSDPGFYLELDQTKQYNEGLGSADPLGENGEQVVFRDSPLTAGPDTHYIRYAGAEHIVLGGTNGDDILVSSEGDDTVWGDAGNDRIEGGDGNDQLRGGAGDDIISDMGGDDNIQGGDGNDVLHGGNGVNLIIGGFGNDFIVTGEDASEAIGGQGNDFILGSKA

>A5W572-A

LPNSQVPFGLRTVDGSYNNLVTGQSEFGAADNSFLRLLEASYRANYVGTGNVVDSQPRTISNLIVDQTANNPAAVEANGGAAPVMSPGIDGVFGTADDKPVFFIPNVSPDAGLTAGFNAWMTFFGQFFDHGLDLVSKSSTDIVFIPLRPDDPLFVAGSPTNFMVLSRAVRTAGADGVVGTADDSQPNTTSPFVDQSQTYSSHPSHQVFLREYTVNAAGEPVATGRLITNRDLGADGKFGTADDGNGESGGMATWAVVKAQARDLLGINLTDADVHSVPLLATDAYGNFLRGPNGMPQVVVRVNNGADGIAGTADDVTQLVEGDRNAPISLANAVSTGHGFLDDIAHNAAPVVVGGVLQADADTLVGNAQPVGQGGNNLTYDNELLDAHYIAGDGRVNENIGLTAVHHVFHSEHNRLVQQTKDTLLAAGDLAFLNEWLIDDVTAIPTAPADIAALVWDGERLFQAAKFGTEMQYQHLVFEEFARTIQPQIDEFLAPNGYDTSINPAILAEFAHVVYRFGHSMLTETVDRYDPAFNPVSADPANPDQQLGLIAAFLNPLAFAGSGATADEAAGAIIRGVTRQVGNEIDEFVTEALRNNLLGLPLDLPALNIARGRDTGIPSLNEARREFYAATGDSQLKPYISWVDFADHLKHPASLINFIAAYGTHSSITGATTEAAKRAAAVALVLGGDGAPLDRLDFLNGTGAYANVTLAGADGIAGTADDIAGVTVTGVDAIDFWVGGLAEKKMPFGGMLGSSFNFVFETQLEALQNGDRFYYLSRTAGMNFGTELENNSFAKLIMANSDVTHLSNTVFLTPTFTLEVNQANQFTGLGADGKADPTGGIEINGVEIVPLVIRDNPDTVGPDGNFLHYTGEDHVVLGGTAGNDIIISSEGDDTLYGDAGDDLLEGGAGNDAVLGGAGDDIITDSFGDNRLEG

>A5W572-B

IPNVRAAMGLRAVDGSNNNLMNLNGNNNTQYGAADNVFPRVTDPVFNPAEGAPAGFFGPGSPAIPGSSYQQTSGPVFDSQPRTISNLIVDQTSNNPAAYATAYDPGADGVLNFGAAGNDDVLKDGVRIVASPGMDGQFGTTDDHDVYLFENTAADAGLSAPFNAWMTFFGQFFDHGLDLVTKGGSGTIYIPLQPDDPLYVEGGFTNFMVVTRATNLPGPDGILGNADDIREHTNTTTPFVDQNQTYSSHPSHQVFLRAYVMTDDGPVATGRLITNRDLGADGRFGTADDTEIGGMATWKVVKAQARDLLGINLTDADVDNVPLLATDAYGNFIKGPNGYPMVVMKGLDGIAGTADDQEVEGNPLAPLDLTNAVRTGHQFLADIAHNAVPVFSGGVLAPDADNVAGNAVPVNPQTGANLAYDNELLDAHYIAGDGRVNENIGLTAVHAIFHSEHNRLVAQTMDTVLDSGDLAFLNEWLLNPVSALPVTPAEIDALVWNGERLFQAAKFGTEMQYQHLVFEEFARTVQPRVDLFFAPTQVYDVDLDASIVAEFAHTVYRFGHSMLTETVDRFDIDFNVIQDPASANPDQQLGLIAAFLNPLAYAASGVTPEDATSAIVRGVTRQAGNEIDEFVTEALRNNLLGLPLDLPAINIARGRDVGIPSLNAVRREIYGQTGDTQLKPYSSWVDLVQHLKHPESLINFIAAYGTHSTITAATTLLEKRAAAMALVFGGDGAPADRMDFLNSSGAWANVTLPGKDGVLGTADDLKAVTVTGVDAIDLWIGGLAEAKAPFGGMLGSTFNFVFENQMEKLQDGDRFYYLERTAGLSMNAELESNSFAKLIMANSSATHLPGLVFSDPGFYLELDQTKQYNEGLGSADPLGENGEQVVFRDSPLTAGPDTHYIRYAGAEHIVLGGTNGDDILVSSEGDDTVWGDAGNDRIEGGDGNDQLRGGAGDDIISDMGGDDNIQGGDGNDVLHGGNGINLIIGGFGNDFIVTGEDASEAIGGQGNDFILGSKA

>B0KJL7-A

LPNSQVPFGLRTVDGSYNNLVAGQSEFGAADNSFLRLLDASYRANYVGTGNVVDSQPRTISNLIVDQTANNPAAVEANGGAAPVMSPGIDGVFGTADDKPVFFIPNVSPDVGLTAGFNAWMTFFGQFFDHGLDLVTKSSTDIVFIPLRPDDPLYNASSPTNFMVLSRAVRTAGADGVVGTADDGQPNTTSPFVDQSQTYSSHPSHQVFLREYMLDAAGDPVATGRLITNRDLGADGKFGTADDGNSENGGMATWAVVKAQARDLLGINLTDADVHSVPLLATDAYGNFLRGPNGMPQVVMRVNNGADGIAGTADDVTTLVEGNRAAPISLANAVSTGHGFLDDIAHNAEPVKVGGVLQADADSAVGNVQPVGPGGNNLTYDNELLDAHYIAGDGRVNENIGLTAVHHVFHSEHNRLVQQTKDTLLAAGDLAFLNEWLIDDVIAIPTTPAGIAALVWDGERLFQAAKFGTEMQYQHLVFEEFARTIQPQIDEFLAPNGYDTSINPAILAEFAHVVYRFGHSMLTETVDRFDPSFNPVSGDPANPDQQLGLIAAFLNPLAFAGSGATADEAAGAIIRGVTRQLGNEIDEFVTEALRNNLLGLPLDLPALNIARGRDTGIPSLNEARREFYGATGDSQLKAYISWADFADHLKHPASLINFIAAYGTHSSITGATTEAAKRAAAVALVLGGAGAPADRLDFLNSTGAWANVTLAGKDGIAGTADDIAGVTVSGVDAIDFWVGGLAEKKMPFGGMLGSSFNFVFETQLEALQNGDRFYYLSRTAGMNFGTELENNSFAKLIMLNSDVTHLSNTVFLTPTFTLEVNQANQFTGLGADGKADPTGGIEINGVEIVPLVIRDNPDTVGPDSNYLHYTGEDHVVLGGTSGNDIIISGDGDDTVYGDAGDDVLEGGAGNDAVLGGAGDDIITDSFGDNRLEGNAGNDVIVAGS

>B0KJL7-B

IPNIRAPLGLRAVDGSNNNLMNLNGHNNTQFGAADNVFPRLTDPVFNPAEGAPAGFFGPGSPAIPGSSYQQTSGPVFDSQPRTISNLIVDQTSNNPAAYATAYDPGADGVLNFGAPGNDDVLKDGVRIVASPGMDGQFGTTDDHDVYLFENTAADAGLSAPFNAWMTFFGQFFDHGLDLVTKGGSGTIYIPLQPDDPLYVEGGFTNFMVVTRATNLPGPDGILGNADDIHEHTNTTTPFVDQNQTYSSHPSHQVFLRAYVMTDDGPVATGRLITNRDLGADGKFGTADDTEIGGMATWKVVKAQARDLLGINLTDADVDNVPLLATDAYGNFIKGPNGYPMVVMKGVDGIAGTADDQQVEGNPLAPISLTNAVRTGHQFLADIAHNAVPVFSGGVLAPDADNAVGNAVPVNPQTGANLAYDNELLDAHYIAGDGRVNENIGLTAVHAIFHAEHNRLVAQTMDTVLDSHDLAFLNEWLLNPVTALPVTPAEIDALVWNGERLFQAAKFGTEMQYQHLVFEEFARTVQPRVDLFFAPTQVYDVDLDASIVAEFAHTVYRFGHSMLTETVDRFDIDFNVIKDPASANPDQQLGLIAAFLNPLAYAASGVTPEDATSAIVRGVTRQGGNEIDEFVTEALRNNLLGLPLDLPAINIARGRDVGIPSLNAVRRDVYGQTGDTQLKPYTSWVDLVQHLKHPESLINFIAAYGTHSSITGATTLLEKRAAAMALVFGGEGAPADRLDFLNSSGAWANVTLPGKDGVLGTADDLKAVTITGVDAIDLWIGGLAEEKTPFGGMLGSTFNFVFENQMEKLQDGDRFYYLERTSGLSMNAELESNSFAKLIMANTSAAHLPGLVFSDPGLYLELDQSKQYNDGLGHADPLGENGEQVVFRDSPLTAGPDSNYIRYAGAEHIVLGGTNGDDILVSSEGDDTVWGDAGNDRIEGGDGNDQLRGGAGDDIISDMGGDDNIQGGDGNDVLHGGNGVNLIIGGFGNDFIVTGEDASEAIGGQGNDFILGSKANEQDMGNEGDD

>Q1YMS2-A

NDLVPDPHVPWGLRTVDGTYNNLVDGREQWGAADTVMPRYLDGSFVTDTNSGAFFGVTNNNYAAPGSVVDTDPRIISNLIVDMSVDNPAAVLAFLNNELAVETFKELHGGLEPVAPGTVVNSATQLAVTDADLALIPNIAPDEGISAPFNGWTTFFGQFFDHGLDLITKGTNGTVYIPLQPDDPLYVPGGFTNFMVLTRAAKAEHLPGEDGVLGTADDIVSHTNTTTPFVDQNQTYTSHASHQVFLREYKFNADGEPVSTGRLLDGLEGGLATWGQIKAEAAAKLGIALDDQDALNIPLLRTDPYGEFIRGDNGLPMIVTGLGPDGIPNTADDIVVQGNLTTPVNTMAIGAIRIGHAFLDDIAHNAAPVINGGVLQPDADVLTGNTVASQQGQNTEYDNELLDRHYITGDGRGNENIALTAVHHVFHSEHNRLVDATRMEVLKSGDLAFINEWLATDIATLEGIPADGLPLLNFANTLDWDGERVFQAARFGTEMQYQHLVFEEFARKIQPAIDPFVFNSSTDIDPSIFSEFANVVYRFGHSMLTETVARTNIHDGSADNIGLIQAFLNPVEFTKNSTVSADEATASIVLGMTSEHGNAIDEFITSALRNNLLGLPLDLAAINIARGRDTGMPTLNETREQLYQATGSSFLKPYDSWVDFAANLKNPMSVVNFIAAYGTHETIVAAGNNLQERRNAAMALVFNTEGAPADRLAFLNSTGGETAESVGLNDIDLWVGGLAEQILLFGGMLGSTFAAIFEAQLEALQDGDRFYYLSRTQGLNLLNELENNAFSKLIIANTDLSDPGPDGIRGTGDDVIARHIGVDAFGQYDYVLEVNKSNQLIEDPTGVDPVLEALGLGKVIRDDPRTPQDESAVSGYVASINALVKQYDASGTPTGILIDGSENVIPGITVGDLRENAEHLGIILTDEDLANAPVLKLNPDGTLRFN

>Q1YMS2-B

DDDTAGAGGVRELLGRNNNESHPEYGAADEVFIRLTEARYGEYDGTTNNRAINPIFAGLDARTISNVLGHQEADLSPAASGANTFFMAFGQYFDHGLDFLPKNSANGVLAIGGPGTSRAPGVDNPADLTRGEVYTIDENGVPQHLNKASPFVDQNQAYGSNALVGQFLRESDGDQGVGMRLLSGATDPSTPDFNLLPTLRELIAHHWENDTIFVDPSLPGGSVSFRDYFTDYPISDNATGSIFDEATGAYDPDVVASMVSNFMGGGYPLLLDTNPYINLLDHYVAGDGRANENFALTSMHTIWARNHNFHVEMLLEAGFEGTEEEVFQAAKMINEAEYQRVVFTEFADMLIGGIRGEGDHGFNDYNPNADARISHEFASAVYRVGHSLVGQTMTVIGPDGQPRQVELFDAFLNPTSELGAFKPGLPDGYVPQPGYAQLGAGAILAGVATQSAEEVDFNIVDAIRNDLVRINADLFAFNVARGWDVGLGTLNQVRADLKASGDPYIQEAVGFAGNLDPYASWADFQARNGLSDTIMDQMKVAYPDLILSTPEEIAAFIAVNPDIELTDGANGTKIVKGIDRVDLWVGGLAEKHVLGGMVGQTFWVVLHEQFDRLQEGDRFYYLERFDNFDFYDNFIDGQEFSDIIARNTGLTGLPEEIFRANDENDDTADNDDGVGDDTSDEDGDSDTVGEDNNDDTVVDDDDALAPVPPVVSGLAVTGTAAADVMMGGAEADVLSGGDGDDIILGGFGDDTLMGGSGSDLIKGDAGRDMIFGGAGDDVVL

>Q0G341-A

QLAAHVGSELRVEISHTGGGQALIDNVELSASSGNRIEITDEDLATLPNIAPDDGISAPFNAWMTFFGQFFDHGLDLITKGGNGTVFIPLQADDPLVVSGQVPPHMQFMVLTRSTPTEGPDGSMTEGKNVTTPFVDQNQTYTSHASHQVFVREYEMVDGRPISTGHLLDGANGGLATWAEVKVQAAEKLGIALADGDAVSIPLILTDAYGEFVRGPNGFPQVVLGVGPDGIPNTADDIVVEGDPENPINTFTVQSEDGSLTGAVRIGHAFLDDIAHAANPVDSQTGLLKAEGTYDSELLGRHFITGDGRGNENIALTSVHHVFHSEHNRQVEDQKKTILETGDLEMLNEWLAVDVSEVPTDPAVIATLSWDGERLFQAARFATEMQYQHLVFEEFGRKINPNIDPFVFNAVTDINPAIFAEFANVVYRFGHSMLTDNMPRVFVDETTGEVSTDDMGLIQAFLNPDVFKRDGNDNEISADEAAAAIVRGMTTERGSAIDEYVVSSLRSNLLGLPLDLPALNIARGRETGMPTFNDARAELYGQTNSVWLKPYESWADLAQNLKTPMTVVNLIAAYGLHETVTGATTLADKRAAAFDLVFGSESLNDTDRLDFMLSRGEWNAANNGLNEIDLWVGGLAERIMPFGGMLGSTFSAIFEAQMEALQFGDRFYYLTRTQGQNLLNELEENAFAKIIMANTNLTLPGPDGIKGTEDDVTPHHIGIDVFADYDFVLEVNKANQLIEDPEGNDPILEALGRKKVLRDDLTTSEVETNYIKFTGGEHIVVGGTNDDDTIITDDGDDAIWGDAGDDYIESGFGVDLVNGGYGNDIILDAGDEGDFLKGDEGDDVMATANGLDVLMGGEGKDAIFLGADASEVFGGEGD

>Q0G341-B

DDDTEGAAGVRELLGRNNNENNPEFGSADEVFIRLTEARYGEYDATINNRAVNPIFAGLDPREISNILGVQEADLAPAKSGANTFFMAFGQYFDHGLDFLPKDSLNGVIEIGGPGSARAPGVDNPADLTRGKVHVIDENGIPQHLNKASPFVDQNQAYGSNELVGQFLRESDGAQGFGMRLLAGADDPSNPEFRLLPTLRELIEHHWEANTIFRDPSLPNGAISFREYFTDFPISEGVTGNLFDEATGAYDPDVVNHLVSDFMGGGYPLLLDTNPFINLLDHYIAGDGRANENFALTSMHTVWARNHNFHVETLMEAGFEGTSEEFFQAAKMLNEAEYQRVVFDEFADFLIGGIRGSGSHGHDEYNPDVDARISHEFAAAVYRVGHSLVGQTMTVIGPDGQPREVALFDAFLNPTNEAGAFTGPLPPGYVPQPGYAQLGVGAILSGTAIQPAEEVDFNIVDAIRTTSFGSMPTCSPSTSLAAGTSGWAR

>A6E280-A

TTVISSVTLPEPADGQWADLTLNSGPISAGLAGQTLRVEIQQTGGSQVLVDNVALSTSNGNEIEIDNIDLATIPNIAPDDGISAPFNAWMTFFGQFFDHGLDLITKGDNGTVFIPLQADDPLVLGADGIAGTADDLPNHLRFMALTRSTPVDGPGADGVLGTADDTQHEGQNTTTPFVDQNQTYTSHASHQVFLREYAFDTNGNPVSTGKLLDGANGGIPTWAEVKAQARDLLGIELTDGDVLNIPLLRTDDYGEFIRGPNGLPQIVVGIGADGIPNTADDDVVEGNLAAPVNTFTAGAIRIGHAFLDDIAHAANPFDSQSGMLKTADDDTAVGLSDSVSTAGTYDNELLDRHFVTGDGRGNENIGLTAVHHVFHSEHNRQVVAQKKTILESGDIDFINEWLLVDLAAGDPIPTDPTALTWDGERLFQAGRFATEMQYQHLVFEEFGRKIHPNIDPFVFNAVTDINPSIFAEFANVVYRFGHSMLTENMPRVLVNELTGEVTTDNMGLIAAFLNPVAYDNDGAMSADAAAAAVILGMTTEQGSQIDEFIVPALRSNLLGLPLDLAAINIARGRDTGIPSFNDARAELFQQTNSVWLKPYENWVELAANLKTPMTIVNLLAAYGTHSTILAANTLEEKRDAAFDLVFGGGGVSDADRFDFLLGRNGWTSDTNGLNTIDLWVGGLAERIMPFGGMLGSTFTAIFEAQMEALQDGDRFYYLTRTQGQNFLNELEENSFSKMLLANTSLADPGADGIRGTEDDVVRHHIGVDSFARYDFVLEVNQANQLIDDPVGNDPVLEGLGMGKVVRDDPTTSEVETNYIRVTGGEHLAVGGTNGNDTIITSDGDDGIWGDDGDDFIESGFGVDLVNGGGGNDIILDSGDEGDFLKGEGGDDVMASAN

>A6E280-B

DDDTEEATGVRTLSGEGNNEANPAYGAAGEPFIRLTEARYGDPDENGNRQINPIFDGLDPRAISNILGPHDDTTAPNAMNASALFMAFGQYFDHGLDFIAKNPAFGTIEIGGPGAERSPTSDNPADLTRAEVAGYDEDGVPQHTNMTSPFVDQNQAYGSHELVGQFLRESDGAHGFGMRLLGGEADPSDPAFTLLPTLRDLILHHWEAETYFEDPGLPGGAARLQDVFPDLVDEITGEIDPDMVQALASDFLGSGQPLLLDANPFIDLLDHRMAGDGRANENFALTSVHTVWARNHNFHVENMLAQGFEGSDEEIFQAAKMLNESDYQRVVFQEFADKLLGGLRNADGDREDHGWDGYNPDVDARISHEFAAAAYRFGHSLVGENLQVQGPNGELIQVPLYDAFLNPSNDPSVFNGPLPQGYVPAPGYAQYGVAAIIGGTAVQAAEEVDLKIVEAIRSDLVRINADLFSFNVARGWDVGLGTMNQVRTQLAASTDPYVSQAVDMAGDLSPYSSWADFQARNDVSDEDMARLMEAYPDLVLETPAQIAAFVAVNPDVVLEDGANGAKIVKGIDRVDLWTGGLAEKHVNGGMVGQTFWVVLHEQLDRLQEGDRFYYIDRFDNFDFYQEFGEDTTFASIVARNTSLTDIDNNLFDANGIDDDDDNATEDDNATEDDNATEDDDDTAGGDDDNATDGDDDTAGGDEDNATDGDDDTADGDEDTASDDQDDTAGDNDTEGDEDDDEPEDNDSNVTPPLAASGNVIGTAAADALFGGAEGDNILALAGRDMIFAGDGDDNVLAGSGRDMIFGDGGNDRLFGEGGDDFIEGG

>B7KW13-A

AIADPHVPYGLRTVDGTYNNLVPGRETWGSSGQPMPQLFEPTYLNDADGDTMALGPGAPVITNTNYGLPGSVADADPRIISNLVVDATLDNPAAIAAALRIAGSENVIADQRAITAAHEALKAAQAANPAGDHAVLQSNLDALLEQTGVTVTNGSIDVLNVSPDEGLSKPFNAWMTFFGQFFDHGLDLISKGGNGTVYVPLAADDPLVLGQDGLAGTADDLAPHLRFMTLTRAAQVEGSQRNVTTPFVDQNQTYTSNASHQVFLREYALVDGRPVATGRLLGGADGGLATWADVKFQARTILGIELTDADVSAVPQLLVDAYGEFVRSANGLPQVMVGVGPGGQAVYASGSLAEPLKLSAIQLPVGTVLVGPNGAQNVIEAGETVAAARTFNAFLDDIAHNAVPVAVNGVLRPDADALTGNAVQMNPQTGRNLEYDNELLDRHFVTGDGRGNENIGLTAVHHIFHSEHNRQIDAHKLTILQSGDLAFINDWLATDIAALPGNFAQMTPLGQLAYANTLSWDGERLFQAARFATEMQYQHLVFEEFARKIQPLVDPFVFNPVTEIDPSIFAEFANTVYRFGHSMLTENMPRLGPDGQALDADLGLIDAFLNPLAFDNDGGLSHDESAAAIMRGMTIERGSEIDEFVVGALRNNLLGLPLDLAAINIARGRDTGTPTLNEARAQLYAATGSTFLTPYTSWVEMAANLKNPLSVVNFIAAYGTHGTVVAATTLAAKRDAAMALVFGGDGAPTDRLDYLNSRGSWAGRETGFGAVDLWIGGLAEKQMPFGGMLGSTFNAIFEAQMENLQDADRFYYLSRVQGQNFLNELEQNSFSKIMLANSSLSLPGPDGIRGTADDIVPRHIGVDAFADYDFELEVNAANQLDQNGAAPGRDPTGNDPVLEAMGLGKVVRDDPGTAADEGASGFHASVNALVRRFGADGSPTGALVDGSEDGVGGAGSPVTWADLKANAAKLGIALTQADMLDAPVLRIGA

>B7KW13-B

PDNAGDDDVPTGYRELSGHGNNLDHPTWGSADQAFIRLTQARYGEADANGNRAINPIFDGLDARTISNILGTQEAGLPKAGNDANIFFMAMGQYIDHGLDFLPKGGNGSIVIGAPGGGAPGSNNPADLTRGTVMAVDANGVPQHKNQTSPYIDQNQAYGSNALVGQFLRESDGAQGVGMRLLAGAPDPSNPAFNLLPTLRELVNHHWQADTIFAGPDGPISFRTYYTNFALSEGVTGTLFNTETGAFDPQVLTKLVGNFMGSGHPLLLDTNPFISVLDHFVAGDGRANENFALTSIHTVWARNHNYHVEKLLESGFEGTPEQVFQAAKMVNEAEYQRVVFDEYLETLIGGLRSDGTHGFEAYDPNVDVAISHEFAAAVFRFGHSLIGQTLNVKGADGETVPVSLFDAFLNPSNDPSVFTAPLPPGYVPQPGYAQYGVGGIIGGTIEQAAEDVDFNIVDAVRNDLVRIRADLFAFNVARGWDVGLGTLNQVRADLAASTNPYIRDAVGFAGGDLSPYASWEDFQARNGLSDAVIAQFRQAYPDLVLAAADIAAFRAINGDIAIAMQADGTGVVKGIDRLDLWVGGLAEKHINNGVVGQTFWVVLHEQFDRLQDGDRFYYLERFDNFDFYENLVDGQGFSDIVARNTGLTVLPEHIFELSDEDGPGTEPGDDDDDDDGVTDPVGGDPDEDEDGPTDPVGGGGDAGGDEDEDDGVTDPVGGGDDPGDDEDDGQGDGDGTTDPVGGGDGDEDGPGTGPGTNPPVNHAPGVIAGGANGDVL

>C7CGY0-A

AIADPHVPYGLRTVDGTYNNLVPGRETWGSSGQPMPQLFEPTYLNDADGDTMALGPGAPVITNTNYGLPGSVADADPRIISNLVVDATLDNPAAIAAALRIAGSENVIADQRAITAAHEALKAAQAANPAGDHAVLQSNLDALLEQTGVTVTNGSIDVLNVSPDEGLSKPFNAWMTFFGQFFDHGLDLISKGGNGTVYVPLAADDPLVLGQDGLAGTADDLAPHLRFMTLTRAAQVEGSQRNVTTPFVDQNQTYTSNASHQVFLREYALVDGRPVATGRLLGGADGGLATWADVKFQARTILGIELTDADVSAVPQLLVDAYGEFVRSANGLPQVMVGVGPGGQAVYASGSLAEPLKLSAIQLPVGTVLVGPNGAQNVIEAGETVAAARTFNAFLDDIAHNAVPVAVNGVLRPDADALTGNAVQMNPQTGRNLEYDNELLDRHFVTGDGRGNENIGLTAVHHIFHSEHNRQIDAHKLTILQSGDLAFINDWLATDIAALPGNFAQMTALGQLAYANTLSWDGERLFQAARFATEMQYQHLVFEEFARKIQPLVDPFVFNPVTEIDPSIFAEFANTVYRFGHSMLTENMPRLGPDGQALDAGLGLIDAFLNPLAFDNDGGLSHDESAAAIMRGMTIERGSEIDEFVVGALRNNLLGLPLDLAAINIARGRDTGTPTLNEARAQLYAATGSTFLTPYTSWVEMAANLKNPLSVVNFIAAYGTHGTVVAATTLAAKRDAAMALVFGGEGAPTDRLDYLNSRGSWAGRETGFGAVDLWIGGLAEKQMPFGGMLGSTFNAIFEAQMENLQDADRFYYLSRVQGQNFLNELEQNSFSKIMLANSSLSLPGPDGIRGTADDIVPRHIGVDAFADYDFELEVNAANQLDQNGAAPGRDPTGNDPVLEAMGLGKVVRDDPGTAADEGASGFNASVNALVRRYGADGSPTGALVDGSEDGVGGAGSPVTWADLEANAAKLGIALTQADMLDAPVLRIGADGRLAFAPGSSVPEAVAVTNGSFEGLALVAGQEGVILDGNGN

>C7CGY0-B

PDNAGDDDVPTGYRELSGHGNNLDHPTWGSADQAFIRLTQARYGETDANGNRAINPIFDGLDARTISNILGTQEAGLPKAGNDANIFFMAMGQYIDHGLDFLPKGGNGSIVIGAPGGGAPGSNNPADLTRGTVMAVDANGVPQHKNQTSPYIDQNQAYGSNALVGQFLRESDGAQGVGMRLLAGAPDPSNPAFNLLPTLRELVNHHWQADTIFAGPDGPISFRTYYTNFALSEGVTGTLFNTETGAFDPQVLTKLVGNFMGSGHPLLLDTNPFISVLDHFVAGDGRANENFALTSIHTVWARNHNYHVEKLLESGFEGTPEQVFQAAKMVNEAEYQRVVFDEYLETLIGGLRSDGTHGFEAYDPSVDVAISHEFAAAVFRFGHSLIGQTLNVKGADGETVPVSLFDAFLNPSNDPSVFTAPLPPGYVPQPGYAQYGVGGIIGGTIEQAAEDVDFNIVDAVRNDLVRIRADLFAFNVARGWDVGLGTLNQVRADLAASTNPYIRDAVGFAGGDLSPYASWEDFQARNGLSDAVIAQFRQAYPDLVLAAADIAAFRAINGDIAIAMQADGTGVVKGIDRLDLWVGGLAEKHINNGVVGQTFWVVLHEQFDRLQDGDRFYYLERFDNFDFYENVVDGQGFSDIVARNTGLTVLPEHIFELSDEDGPGTEPGDDDDDGDTDPVGGDPDEDEDGPTDPVGGGGNAGGDEDEDDGVTDPVGGGDDPGDDEDDGQGDGDGTTDPVGGSDGDEDGPGTGPGTTPPVNHAPGVIAGGANGDVLNGTAGADTILGLDGDDNILAGGGADVVRAGAGNDFVDAGEGRDV

>A9W3A5-A

AIADPHVPYGLRTVDGTYNNLVPGRETWGSSGQPMPQLFEPTYLNDADGDTMALGPGAPVITNTNYGLPGSVADADPRIISNLVVDATLDNPAAIAAALRIAGSENVIADQRAITAAHEALKAAQAASPAGDHAVLQSNLDALLEQTGVTVTNGSIDVLNVSPDEGLSKPFNAWMTFFGQFFDHGLDLISKGGNGTVYVPLAADDPLVLGQDGLAGTADDLAPHLRFMTLTRAAQVEGSQRNVTTPFVDQNQTYTSNASHQVFLREYALVDGRPVATGRLLGGADGGLATWADVKFQARTILGIELTDADVSAVPQLLVDAYGEFVRSVNGLPQVMVGVGPGGQAVYASGSLAEPLKLSAIQLPVGTVLVGPNGAQNVIEAGETVAAARTFNAFLDDIAHNAVPVAVNGVLRPDADALTGNAVQMNPQTGRNLEYDNELLDRHFVTGDGRGNENIGLTAVHHIFHSEHNRQIDAHKLTILQSGDLAFINDWLATDIAALPGNFAQMTALGQLAYANTLSWDGERLFQAARFATEMQYQHLVFEEFARKIQPLVDPFVFNPVTEIDPSIFAEFANTVYRFGHSMLTENMPRLGPDGQALDADLGLIDAFLNPLAFDNDGGLSHDESAAAIMRGMTIERGSEIDEFVVGALRNNLLGLPLDLAAINIARGRDTGTPTLNEARAQLYAATGSTFLTPYTSWVEMAANLKNPLSVVNFIAAYGTHGTVVAATTLAAKRDAAMALVFGGEGAPTDRLDYLNSRGSWAGRETGFGAVDLWIGGLAEKQMPFGGMLGSTFNAIFEAQMENLQDADRFYYLSRVQGQNFLNELEQNSFSKIMLANSSLSLPGPDGIRGTADDIVPRHIGVDAFADYDFELEVNAANQLDQNGAAPGRDPTGNDPVLEAMGLGKVVRDDPGTAADEGASGFHASVNALVRRYGADGSPTGALVDGSEDGGGDAGSPVTWADLKANAAKLGIALTQADMLDAPVLRI

>A9W3A5-B

PDNAGDDDVPTGYRELSGHGNNLDHPTWGSADQAFIRLTQARYGEADANGNRAINPIFDGLDARTISNILGTQEAGLPKAGNDANIFFMAMGQYIDHGLDFLPKGGNGSIVIGAPGGGAPGSNNPADLTRGTVMAVDANGVPQHKNQTSPYIDQNQAYGSNALVGQFLRESDGAQGVGMRLLAGAPDPSNPAFNLLPTLRELVNHHWQADTIFAGPDGPISFRTYYTNFALSEGVTGTLFNTETGAFDPQVLTKLVGNFMGSGHPLLLDTNPFISVLDHFVAGDGRANENFALTSIHTVWARNHNYHVEKLLESGFEGTPEQVFQAAKMVNEAEYQRVVFDEYLETLIGGLRSDGTHGFEAYDPSVDVAISHEFAAAVFRFGHSLIGQTLNVKGADGETVPVSLFDAFLNPSNDPSVFTAPLPPGYVPQPGYAQYGVGGIIGGTIEQAAEDVDFNIVDAVRNDLVRIRADLFAFNVARGWDVGLGTLNQVRADLAASTNPYIRDAVGFAGGDLSPYASWEDFQARNSLSDAVIAQFRQAYPDLVLAAADIAAFQAINGDIAIAMQADGTGVVKGIDRLDLWVGGLAEKHINNGVVGQTFWVVLHEQFDRLQDGDRFYYLERFDNFDFYENVIDGQGFSDIVARNTGLTVLPEHIFELSDEDGPGTEPGDDDDDGDTDPVGGDPDEDEDEDGPTDPVGGGGDAGGDEDDGVTDPVGGGDDPGDDEDDGQGDGDGTTDPVGGGDGDEDGPGTGPGTNPPVNHVPGVIAGGANGDVL

>Q13AU2-A

AVADPKVPVGLRTVDGQDNNIVPGREQWGAADQSMPRLLTASYTTGAGSIDLNGPAPGGVVTGGNYAAPGTIVDTAPRTVSNLIVDMSLNNPAAIIAALTFAGSEDVLADQSEITAAFLALKAARDADPLGDHAALQLELDTILEQKGVTVTNGSIDVPNVAPDEGLSAPFNAWMTFFGQFFDHGLDLISKGGAGTVYVPLAADDPLRTHGPDGIAGTGDEVPGQMAFMALTRATPAADGSQVNTTTPFVDQNQTYTSHASHQVFLREYKMVGGVPMATGKLLGGAEGGLATWADVKAQAQNMLGIVLSDLDVLNVPLLRTDAYGEFIRDANGFAQVIIGLGDDGIPNTADDLVASGTNLAPLNLATLNNGAGPVRTSHAFLDDIAHNAAPVVVDGVLTPDTDSATGNDVAINPLTGQRLEYDDELLDRHYITGDGRGNENIGLTAVHHIFHSEHNRQVDSQKLTILNSGDIAFINEWLATDIGALDPGFGTMTALQQLDYANSLNWDGERLFQGARFATEMQYQHLVFEEFARKIQPAIDPFVFNSVTDINPAIFSEFANTVYRFGHSMLTEAMPRLDANGNPMDSELGLVESFLNPVLFDNDGAISHDAGAAAIVRGMTIERGNEIDEFVVDALRNNLLGLPLDLAAINIARGRDTGMPSLNETRTQLYAASGSTFLKPYDHWVDLATNLKNPASIVNFVAAYGTHATIVGATTLEAKRMAAMELVFGVDQNGDATVAADRTAFLTGTGAWAGVETGLNRIDLWIGGLAEKKMPFGGMLGSTFNAIFELQLENLQDGDRFYYLTRTQGQNFLNMLEQNSFAKLIMANTDLAQPGPDGIRGTADDIVPRHIGVDSFANYDYVLEVDESNQADYNGAAPGKDPQGADPFLEALGLGKVIRNDPGTAGPDENYIRFSGGEHIVVGGTDGDDTIITDFGDDGIWGDAGDDRIESGAGVDLVNGGAGNDIITDSGDTG

>Q13AU2-B

GDDDDGATGVRDLSGHNNNVANPNWGSADQPFIRITNPHYGEADANGNLAINPVFDGLDPRTISNVLGSQEAGLPSAGNDANIFFMAMGQYIDHGLDFLGKGGNGTIQIGALGGGAPGSGNPADLTRGSVASYDANGVPQHINRTSPYVDQNQAYGSNDLVGQFLRAGDDNGGLGAHLFAGAPDPSNPEFALLPTLRELITEHWQNNTVFHSSSLQGGSVAFRDYFAGLVGQNGVINQALLPSMISNFMGTNHALLLDANPFINVLDHYVSGDGRTNENFALTSIHTIWARNHNHHVEGLAAAGFQGTAEELFQAAKMINEAEYQRVVFDEYLETLLGGLRSQGTHGFEEYDPNANAGISHEFAGAVFRFGHSLIGQTMTVLDANGNPTQVNLFDAFLNPSNDPSAFPNPLPPGYTPQPGYAQHGVNAIIGGTVSQPAEDVDFNIVDAVRNDLVRINADLFAFNVARGWDLGLGTLNQVRRDLAASTNPYVAESVGFAGSNLTPYSSWEDFQQRNDLNNAVIAQFKQAYPDLTLAAADIAAFREVNPDIAIAMQNDGTGIVSGIDRLDLWVGGLAEKHINGGLVGETFWVVLSEQFERLQDGDRFYYISRFDNFDFYENFIDGQQFADIVTRNTGMTGIPEHMFQTDPIDQNENEGEGEGEEDGTPVGNGDPPTDDDDDDDDDDDDDDGDGTAGGDDDDDDDTPPANGEGDGTTPPVGGVIRTGTPQPDVLVGGAGDDNIVAFADDDVIAADAGADAISAGDGNDFVTAGAGRD

>Q07SX1-A

AIADPKVPAGLRTVNGEDNNIVPGREEWGAADQSMPRLLTSSYTTGAGTLDLNGPAPGGAVTGGNYAGPGTIVDTAPRTVSNLIVDMSLNNPAAIIAALTFAGSGDVLGDQGEITAAFLALKAARDADPLGDHATLQQALDDVLEQKGVTVTNGSIEVPNVAPDEGLSAPFNAWMTFFGQFFDHGLDLISKGGAGTIYVPLAADDPLRTHGPDGVAGTGDEVSEQMAFMALTRATPASDGSQTNTTTPFVDQNQTYTSHASHQVFLREYSMASGVPMATGKLLGGADGGLATWADVKAQARDALGIELSDLDVFNVPLLRTDPYGEFIRDANGFPQVIVGIGADGIPNTADDIVASGTNLAPFDLASLNGGLGPVRTSHAFLDDIAHNAAPVVVGGVLAPDADSATGNDVAINPLTGQRLEYDNELLDRHYITGDGRGNENIGLTAVHHIFHSEHNRQVDSQKLTILRSGDTAFINEWLATDIGGLPSGFASLSGLDQLAYANSLNWDGERLFQAARFATEMQYQHLVFEEFARKIQPAIDPFVFNSVTDINPAIFSEFANTVYRFGHSMLTEGMPRLDGAGNSMDSDLGLVEAFLNPVLFDNDGAISHDAGAAAIVRGMTIERGNEIDEFVVDALRNNLLGLPLDLAAINIARGRDTGMPSLNDARTQLYAASGSTFLKPYDHWVDFAANLKNPASIVNFVAAYGTHSTIAGATTLEAKRLAAMELVFGVDQDGDATVAADRTAFLTGTGAWAGVETGLNRIDLWIGGLAEKKMPFGGMLGSTFNAIFELQLENLQDGDRFYYLTRTQGQNFLNMLEQNSFAKMIMANTDLAQPGPDGIRGTADDIVPRHIGVDSFADYDYVLEVDEANQEDYNGAAAGKDPHGADPFMEALGLGKVIRDDPGTAGPDANYIRFSGGEHIVVGGTSGNDTIITDFGDDGIWGDDGDDRIESGAGVDLVNGGAGNDIITDSGDTGDFIKGDEGDDVIANSNGIDILMGG

>Q07SX1-B

PANGDDDGGPTGVRELSGHNNNQANPNWGAADQPFIRITNAHYGDEDANGNRAINPVFDGLDPRTISNILGTQEANLPHAGNDANIFFMAMGQYIDHGLDFLGKGGNGSIQIGAAGGGAPGSDNPADLTRGSVAGYENGVPQHVNRTSPYVDQNQAYGSNDLVGQFLREGDGNGGVGAHLFAGGPDPSNPQFSLLPTLRELIEEHWSNNTVFHSESLPDGAVAFRDYFPGLVQGGVINTAMLPGMISNFMGTSHALVLDANPFINVLDHYVAGDGRANENFALTSIHTIWARNHNHHVEGLEAAGFQGTAEELFQAAKMINEAEYQRVVFDEYLETLLGGLRSQGTHGFEEYDPGADAGISHEFAAAVFRFGHSLIGQTMTVLDADGNPTQVNLFDAFLNPSNDPSVFPSPLPPGYTPQPGYAQHGVNAIIGGTVSQPAEDVDFNIVDAVRNDLVRINADLFAFNVARGWDVGLGTLNQVRQDLAASTNPYVSEAVGFAGGDLSPYTSWEDFQQRNGLSQAVIEQFKQAYPDLQLAAADVAAFQAINPDIDIAMNDDGTGIVKGIDRVDLWVGGLAEQHINGGLVGQTFWVVLSEQFERLQDGDRFYYISRFDNFDFYENFIDGQEFADIIARNTGMTGLPEHMFRTDPIDDENNQNPDDNEGDDDGTPVGNGDDETDDEDDDDTASGGDDEDDDETAGGDDDDDEITGGDDDDDDETVGGDDDVTPPPSTPGVIRTGTPQPDVLVGSAGDDNIVAFADADVIIADAG

>A3XF15-A

AAQDIETTKQSAFDTATTNLDALNAPGAADAALAAAQAIATEAQTTLDTLLSTHAITMDGNNVMLPDVTPDEGLSAPYNSWMTLFGQFFDHGLDLVGKGGSGTVYIPLQPDDPLYDATSPTNFMVLTRATNQPGPDGILGTADDVREHFNKTTPWVDQNQTYTSHPSHQVFLREYDLDANGSPVSNGYLLHGQSGGMSTWGDVKAQAAAKLGIQLNDSDVLDGPLLATDAYGNFIPGANGLPQLVVANPAYVEGGTEPLNILIEGDLANPVDASQAVRNGHAFLEDIAHNAVPGTYVVDRFTGETATKQADADTDTGNAIIPNQFGQNETYDNELLDRHFIAGDGRGNENFGLTAVHHVFHSEHNRQTTEMKQTILDSGELAFINEWLATPINEDELSTAAIDTLTWDGGRLFQAAKFTTEMQYQHLAFEEFGRTVQPQIAAFMVNASAEVDASIMAEFAHVVYRFGHSMLTENVQTMDPNGVNTSTGLIEAFLNPVAFDLDQTLTSDQAAGAVARGMSRETGANIDEFITSALRDNLVGLPLDLAALNITRGRDTGVPSLNAAREQFYAATGSEFLKPYEGWSDYAANLKNPASIINFIAAYGTHDTIANATTVVQKRAAATDLVLGGDTAPADRLDFVNGTGAWATIETGINAVEYWIGGLAEAIMPFGGMLGSSFGFAFQQQMEALQNGDRFYYLSRTNGMDMLGGLENNSFASMIMRNTDIADGGAHIPANIFSSMEYILEVDQSVQAMADPVSTELDPFLAAMGTTLVERETGTGDAPLVDGAREYDNLLKFNGGEHVVLGGTDQRDILVGGLGDDALWGGAGDDLLIGDSGVNTFRGGDGNDIIKDGDDIS

>A3XF15-B

NEPADLIVGTRDLEGLTNNLLNPEISGGATLPFSRVTEARYAGIGEDGAGIVNPVFDDLDARAISNALGAQDADAAKAASANMFMMSFGQYFDHGLTFIPKGGHDPITIGGADMGRPSGDNPADLTRATATINPETGEIEHTNITSPVVDQNQVYGSSNLVGQLLRESGSNGGFGAHVLMGQEDPSASGFQLMATLRELLDHHTQAGTVFTDTDKGDVTLEGYYPDLFNEDGTYNAATIKDLSDDFMGEGWPLLIDTNPFMNLLDHFVGGDGRANENVGLTSMHTVWARNHNYHVDQLLASGYDADTPEELFQAARILNIGEYQQVVFNDFADSLLGGLQGSGTHGHDKYDPTTDARISHEFAAAAYRFGHSQIGQSMTLKDVDADGNPFTVEVPLFDIFLNPTNDPDAFTADFGTLEQYGYKPQSGYAQYGVDNILGGLVEQPSEEVDLQVVDAVRNDLVRVSADLFAFNVARGRDVGLGTLNQVKADLAASDNRYISEAIDLSDMSMTPYTDWEDFQARNGLSDEMIAKFQTAYPALVLTVDTEQYDAFVEANPDIALIDNGDGTMTVKGIDRVDLWVGGLAEQHIQDGVVGHTFWVLIHEQLDRLQEGDRFYYVDQIGDLPVYNNFISNLTFGDIVTRNTGMTDLPQDVFSYTGDEIVEDNGTADQQTQQPPVTDDAGNANQADTGQDDTGQDDNGQGQTAQGDDTQGEMTVDNSTQTAQGGASADQEPVSNDDDTIDGGDSSDADTETPASDPSTEQSPNGSDEGLVKSGTDLGDALVGAAGDDILSGHEGNDMLVG

>P05164 (human myeloperoxidase)

GVTCPEQDKYRTITGMCNNRRSPTLGASNRAFVRWLPAEYEDGFSLPYGWTPGVKRNGFPVALARAVSNEIVRFPTDQLTPDQERSLMFMQWGQLLDHDLDFTPEPAARASFVTGVNCETSCVQQPPCFPLKIPPNDPRIKNQADCIPFFRSCPACPGSNITIRNQINALTSFVDASMVYGSEEPLARNLRNMSNQLGLLAVNQRFQDNGRALLPFDNLHDDPCLLTNRSARIPCFLAGDTRSSEMPELTSMHTLLLREHNRLATELKSLNPRWDGERLYQEARKIVGAMVQIITYRDYLPLVLGPTAMRKYLPTYRSYNDSVDPRIANVFTNAFRYGHTLIQPFMFRLDNRYQPMEPNPRVPLSRVFFASWRVVLEGGIDPILRGLMATPAKLNRQNQIAVDEIRERLFEQVMRIGLDLPALNMQRSRDHGLPGYNAWRRFCGLPQPETVGQLGTVLRNLKLARKLMEQYGTPNNIDIWMGGVSEPLKRKGRVGPLLACIIGTQFRKLRDGDRFWWENEGVFSMQQRQALAQISLPRIICDNTGITTVSKNNIFMSNSYPRDFVNCSTLPALNLASWREAS
